# Ethnoveterinary plants of Pakistan: a review

**DOI:** 10.1186/s13002-020-00369-1

**Published:** 2020-05-15

**Authors:** Muhammad Abdul Aziz, Amir Hasan Khan, Andrea Pieroni

**Affiliations:** 1 University of Gastronomic Sciences, Piazza Vittorio Emanuele II 9, I-12042 Pollenzo, Bra, Cuneo, Italy; 2 Department of Botany, Shaheed Benazir Bhutto University, Sheringal, Pakistan

**Keywords:** Medicinal plants, Ethnoveterinary, Pakistan, Pathans

## Abstract

**Background:**

Ethnoveterinary medicine is crucial in many rural areas of the world since people living in remote and marginal areas rely significantly on traditional herbal therapies to treat their domestic animals. In Pakistan, communities residing in remote areas, and especially those still attached to pastoralist traditions, have considerable ethnoveterinary herbal knowledge and they sometimes use this knowledge for treating their animals. The main aim of the study was to review the literature about ethnoveterinary herbals being used in Pakistan in order to articulate potential applications in modern veterinary medicine. Moreover, the review aimed to analyze possible cross-cultural and cross regional differences.

**Methods:**

We considered the ethnobotanical data of Pakistan published in different scientific journals from 2004 to 2018. A total of 35 studies were found on ethnoveterinary herbal medicines in the country. Due to the low number of field studies, we considered all peer-reviewed articles on ethnoveterinary herbal practices in the current review. All the ethnobotanical information included in these studies derived from interviews which were conducted with shepherds/animals breeders as well as healers.

**Results:**

Data from the reviewed studies showed that 474 plant species corresponding to 2386 remedies have been used for treating domestic animals in Pakistan. The majority of these plants belong to Poaceae (41 species) followed by the Asteraceae (32 species) and Fabaceae (29 species) botanical families, thus indicating a possible prevalence of horticultural-driven gathering patterns. Digestive problems were the most commonly treated diseases (25%; 606 remedies used), revealing the preference that locals have for treating mainly minor animal ailments with herbs. The least known veterinary plants recorded in Pakistan were *Abutilon theophrasti*, *Agrostis gigantea*, *Allardia tomentosa*, *Aristida adscensionis*, *Bothriochloa bladhii*, *Buddleja asiatica*, *Cocculus hirsutus*, *Cochlospermum religiosum*, *Cynanchum viminale*, *Dactylis glomerata*, *Debregeasia saeneb*, *Dichanthium annulatum*, *Dracocephalum nuristanicum*, *Flueggea leucopyrus*, *Launaea nudicaulis*, *Litsea monopetala*, *Sibbaldianthe bifurca*, *Spiraea altaica*, and *Thalictrum foetidum*. More importantly, cross-cultural comparative analysis of Pathan and non-Pathan ethnic communities showed that 28% of the veterinary plants were mentioned by both communities. Cross-regional comparison demonstrated that only 10% of the plant species were used in both mountain and plain areas. Reviewed data confirm therefore that both ecological and cultural factors play a crucial role in shaping traditional plant uses.

**Conclusion:**

The herbal ethnoveterinary heritage of Pakistan is remarkable, possibly because of the pastoral origins of most of its peoples. The integration of the analyzed complex bio-cultural heritage into daily veterinary practices should be urgently fostered by governmental and non-governmental institutions dealing with rural development policies in order to promote the use of local biodiversity for improving animal well-being and possibly the quality of animal food products as well.

## Introduction

Ethnoveterinary knowledge (EVK) is a complex body of elements, encompassing concepts, beliefs, practices, skills, and experiences, which are passed vertically or horizontally across generations (mainly orally or via observation of practical skills), concerning animal well-being. This complex body of both knowledge and practices has been and is still fundamental in many rural areas of the globe for assuring the health of livestock and thus the survival of pastoral or agro-pastoral communities [1].

EVK includes many kinds of knowledge and practical skills: ecological knowledge of pastures; ethnoclimatological knowledge of weather forecasting; knowledge of harvesting and/or cultivating and providing animals specific fodder plants that are considered good for their growth and well-being, as well as for increasing the quality of animal-based food products (i.e., dairy products, meat, eggs, honey, and other bee products); recruitment and use of herbal remedies and other natural treatments when animals are ill; ways of managing whole animal breeding systems; and so forth [2].

Ethnoveterinary field studies specifically concerning traditional *herbal remedies* for treating animal diseases are crucial in many rural areas of the world for several reasons: (a) to propose effective and cheaper treatments alternative and complementary to the use of pharmaceuticals, and especially to decrease the abuse of antibiotics in animal breeding that is in turn detrimental to the quality of animal food products; (b) to foster the sustainable use of local medicinal plant resources in animal care and then to contribute to rural development policies; (c) to promote local bio-cultural heritage; and (d) to investigate the link between human and veterinary plant uses in order to possibly assess the origin of herbal practices [1, 3–9].

Ethnoveterinary studies are also vital for envisioning new equilibria between ecosystem “health” and animal and human health systems, to respect and honor non-Western, traditional, orally transmitted, herbal practices devoted to animals, and especially to encourage trans-disciplinary applied research in the field of animal health care (i.e., biomedical potential of ethnoveterinary practices and ingredients, socio-economic and cost effectiveness of herbal animal treatments, sustainable and sovereign rural development policies and strategies based on animal breeding) [10].

Livestock is considered a subsector of agriculture in Pakistan. In the country, the sector contributes 56.3% of the value of agriculture and nearly 11% to the agricultural gross domestic product (AGDP). In this sector, milk is the single most important commodity and the country is ranked fourth in milk production worldwide after China, India, and the USA [11]. This sector plays an important role in poverty reduction strategies, and it may be developed very quickly. It requires macroeconomic preferences for the economy of Pakistan and the vigorous development of rural economic growth [12]. According to a governmental economic survey of Pakistan [13], the national herd includes 29.6 million cows, 27.3 million buffalo, 53.8 million goats, 26.5 million sheep, and 0.9 million camels. Yet, over the past three decades, the livestock sector which in Pakistan employs more than 35 million people has only experienced an average growth of 2.9%, which is insufficient production for the country, due to poor economic policies [14, 15]. Livestock produce meat, milk, eggs, manure, fibers, hides and horns, and demand for these products is rapidly growing due to population growth, urbanization in developing countries, and increased revenue [16]. The livestock sector is often maligned, but it still plays a vital role in the country’s economy by providing draught power, valuable organic animal proteins, and other by-products. Manure and draught power provided by the animals enhance the supply of organic matter to improve land fertility and aid productivity, respectively. More than 10 million animals are engaged in agricultural activities and events [17].

There are many factors inhibiting the growth of the livestock sector in Pakistan including policy issues, rapid deterioration of rangelands, unhygienic eating practices, poor marketing systems, inadequacy of extension services, and insufficient resources. There are several fatal animal diseases in Pakistan including foot and mouth disease (FMD), hemorrhagic septicemia (HS), bovine viral diarrhea (BVD), and black quarter (BQ). Farmers do not regularly vaccinate their animals against these fatal diseases which lowers dairy production. Many cows/buffalos, for example, seem to suffer from mastitis, greatly contributing to the loss of milk production. The consequences of livestock diseases are generally seen as direct impacts only, but in reality they can be quite complex. The diseases affect the productivity of animals and deprive farmers of their possible daily earnings. These fatal diseases lead to morbidity resulting in short- or long-term product loss [18].

Pakistan has been also home to a number of field veterinary ethnobotanical studies conducted in various areas of the country over the last two decades. These studies have sometimes been carried out as part of broader ethnobotanical surveys. The main purpose of these published studies we reviewed was to investigate and document ethnoveterinary herbal knowledge without any consideration of possible cross-cultural, spatial, and/or temporal variations. Since these surveys were conducted in restricted areas and were published in various literature sources, no in-depth reviews have so far analyzed the overall folk uses of plants for animal diseases in Pakistan, and this review wanted to fill this gap. Suroowan et al. [19] recently reviewed the ethnoveterinary plants of South Asia, but the review considered only a few Pakistani studies and no in-depth analysis and interpretation of the data were carried out.

Hundreds of medicinal plants have been used for centuries in folk veterinary systems in all areas of Pakistan. Ethnobotany in Pakistan has partially addressed these veterinary plant remedies, since most of the studies have focused on medicinal plants for humans and only sporadically on wild food plants. Local shepherds and herbalists living in mountainous and marginal areas were and still are particularly knowledgeable in managing animal care via herbal practices [19]. The information presented in the current review can provide baseline data for implementing culturally appropriate rural developments programs and for fostering the actual use of veterinary Complementary and Alternative Medicine.

The main objectives of this study were:

- To analyze all the field studies reporting ethnoveterinary plant uses in Pakistan
- To assess cross-cultural and cross-regional variations in the folk utilization of veterinary plants in Pakistan.

## Methods

### Selection of the ethnoveterinary herbal literature

For this review, all published articles reporting medicinal plants used in traditional veterinary practices in Pakistan were considered. The literature was thoroughly searched using online databases and platforms such as Scopus, PubMed, and Web of Science, ResearchGate and Academia. In searching the databases some key words were used (ethnoveterinary; ethnobotany; Pakistan) to elicit data on the ethnoveterinary herbal practices for the review. Although extensive literature exists on Pakistani ethnobotany, we only targeted those research articles that were published exclusively on ethnoveterinary herbal practices in the country. In total, 35 peer reviewed articles published in international journals, in English, focusing on ethnoveterinary herbal practices were found, dating from 2004 to May 2018 and from all over the country. It is worth mentioning here that a number of reviewed studies missed some crucial ethnobotanical information in terms of plant folk names, voucher specimen numbers, informants and study site selection criteria, and details on remedy preparations, administrations, and animals treated.

### Statistical analysis

Phillips and Gentry [20] adopted the following formula in order to analyze the cultural importance of botanical species:

$$ \mathrm{U}\mathrm{V} is=\sum \mathrm{U} is/\mathrm{N} is $$

where U*is* represents the number of uses mentioned by all informants for a given species *is* (use reports for species *s*) and N*is* is the total number of informants that reported species *s*.

In this review study, species Use Values (UV*s*) and family Use Values (UV*f*) were employed by modifying the equation for UV*is*, as proposed by Tardío and Pardo-de-Santayana [21]:

$$ \mathrm{U}\mathrm{V}s=\sum \mathrm{U}s/\mathrm{N}s $$

where U*s* represents the number of uses mentioned by all pseudoinformants for a given species *s* (use reports for species *s*) and N*s* is the total number of pseudoinformants that reported species *s*;

$$ \mathrm{U}\mathrm{V}f=\sum \mathrm{U}f/\mathrm{N}f $$

where U*f* represents the number of uses mentioned by all pseudoinformants for a given family *f* (use reports for the family *f*) and N*f* is the total number of pseudoinformants that reported family *f*.

In the statistical analysis, we adopted the concept of “pseudoinformant” as described by Tardío and Pardo-de-Santayana [22]. The term “pseudoinformant” in this review referred to each individual considered ethnoveterinary study rather than the original informants that reported plants in each of the conducted field studies.

### Phytopharmacological review

A comprehensive literature survey was carried out to review the pharmacological evidence for the least known medicinal plants reported in the reviewed ethnoveterinary studies and used in the country. In PubMed we searched out every medicinal plant species for their phytopharmacological profiles. Based on their lowest UV*s*, a total of 30 medicinal plants were selected and investigated for their respective pharmacological and/or phytochemical potential. The assumption we made here was that the most commonly used ethnoveterinary plants in Pakistan have already been well studied bioscientifically. These 30 species have thus far been very rarely investigated.

### Cross-cultural and cross-regional comparative analysis

Data obtained from the selected articles were categorized into three major groups: (a) veterinary medicinal plants used by Pathan communities in mountainous regions; (b) veterinary medicinal plants used by non-Pathan communities in mountainous regions; and (c) veterinary medicinal plant used in plain areas. The adopted categorization was made in order to divide the analyzed ethnoveterinary literature into three equal groups, which had to be able to show variations in geographical and cultural variables. Due to an insufficient number of studies on ethnoveterinary practices in Pakistan, it was only possible to consider data coming from all non-Pathan communities grouped together.

To compile the data systematically, an MS Excel spreadsheet was used in which the botanical names of plants, botanical families, parts used, mode of preparation and administration, and disease treated were recorded. The scientific names of the reported taxa were updated using The Plant List database [23]. Data was arranged in tabulated form. To deal with possible gaps in the selected ethnoveterinary studies, a separate table (Table 1, [24–57]) was created, presenting information about the local names of plants, voucher specimen numbers, description of the study area, language, and typology of the study participants in the selected studies. For cross-cultural and cross-regional analysis of reported medicinal taxa and their uses, the extracted data was tabulated, sorted, and organized in MS Excel, and then the results were displayed using Venn diagrams.

**Table 1:** Ethnoveterinary studies of Pakistan considered in the current review

Reference	Number of plant species	Collection of botanical vouchers	Reported local names	Reported methods of preparation	Areas/Regions	Languages	Characteristics of the study participants	Methodological framework (data collection techniques and data analysis)
Abbasi et al. [24]	89	Yes	Yes	Yes	Haripur, Abbottabad, Mansehra	Hindko	Farmers, shepherds, housewives, and herbalists	Participatory rural appraisal (PRA) approach was adopted. Information was collected through semi-structured interviews. Cultural importance index (CI) was used to analyze the data.
Ahmad et al. [25]	22	Yes	Yes	Yes	Thakht-e-Sulaiman Hills	Pashto	Male informants	Snowball sampling and detailed unstructured interviews were utilized as well as group discussions. Informant consensus factor (ICF) and fidelity level (FL) were calculated and applied to the collected data.
Ahmed & Murtaza [26]	24	No	Yes	Yes	District Muzaffarabad	Hindku	Males and females (local healers and shepherds)	Data was collected through semi-structured interviews. Informant consensus factor (ICF) and fidelity level (FL) were used to analyze the data.
Ali et al. [27]	51	Yes	Yes	Yes	Central Karakoram National Park	Balti	Traditional healers and livestock holders (men and women)	Participatory rural appraisal (PRA) approach was adopted. Data was gathered through semi-structured questionnaires and interviews. Informant consensus factor (ICF) was used to analyze the data.
Aziz et al [28]	94	Yes	Yes	Yes	South Waziristan, Bajaur	Pashto	Local peoples (men and women)	Semi-structured interviews were used to collect the data, which was analyzed through informant consensus factor (ICF).
Badar et al. [29]	46	No	Yes	Yes	District Jhang	Punjabi	Traditional healers	Rapid and participatory rural appraisal techniques were used for collection of information i.e. interviews and focus group discussions were utilized to gather the data. Data was not subjected to applied statistics.
Deeba et al. [30]	39	No	Yes	Yes	Faisalabad	Not mentioned	Elders and traditional healers	Rapid rural appraisal (RRA) and participatory rural appraisal (PRA) techniques were used for selection of key respondents. Data was gathered through unstructured interviews. Gathered data was not subjected to applied statistics.
Dilshad et al. [31]	66	No	Yes	Yes	District Sargodha	Punjabi	Traditional veterinary healers	Information was collected using rapid and participatory rural appraisal techniques through interviews and focus group discussions. Data was not subjected to applied statistics.
Dilshad et al. [32]	25	No	Yes	Yes	District Sargodha	Not mentioned	Traditional healers	Information was collected using a well-structured questionnaire, open ended interviews and guided dialogue techniques. Data was not subjected to analysis using any ethnobotanical index.
Farooq et al. [33]	18	Yes	Yes	Yes	Cholistan Desert	Saraiki	Traditional healers and herdsmen (males)	Rapid rural appraisal approach was adopted. Data was gathered using a well-structured questionnaire and through open-ended interviews and guided dialogue techniques. Data was not subjected to applied statistics.
Harun et al. [34]	53	Yes	Yes	Yes	Kasur, Faisalabad, Vehari, Sargodha, Gujrat, Narowal	Punjabi	Males and females, shepherds, and ruminant caretakers	Group discussions and individual ethnobotanical semi-structured interviewing techniques were used for data collection. Data was analyzed through relative frequency of citation (RFC), pair wise comparison method (PC), cluster analysis and descriptive statistics.
Hussain et al. [35]	41	Yes	Yes	Yes	District Sahiwal	Punjabi and Saraiki	Traditional veterinary healers	Participatory rural appraisal approach for data collection using a well-structured questionnaire. Information was collected through interviews and focus group discussions. No ethnobotanical indices were used to analyze the data.
Islam et al. [36]	30	No	Yes	No	Mansehra	Hindko, Gurjar, Pashto	Local people	Data was gathered through questionnaires and interviews but lacked useful information on the type of interview and questionnaire. Data was not subjected to analysis using any ethnobotanical index.
Khan & Hanif [37]	54	Yes	Yes	Yes	District Bhimber, Azad Kashmir	Not mentioned	Healers and male informants, shepherds, farmers, and herbal sellers	Data was gathered through interviews but lacked useful information on the type of interview. Data was not subjected to analysis using any ethnobotanical index.
Khan et al. [38]	83	No	Yes	Yes	District Peshawar	Pashto	Local healers	Data was gathered through a questionnaire and interviews but lacked useful information on the type of interview and questionnaire. Data was not subjected to analysis using any ethnobotanical index.
Khan et al. [39]	35	no	Yes	Yes	Cholistan Desert	Not mentioned	Local pastoralists, veterinary practitioners, and quacks	Data was collected through open-ended interviews and guided dialogue techniques. Data was not subjected to analysis using any ethnobotanical index.
Khan et al. [40]	19	Yes	Yes	Yes	Poonch Valley, Azad Kashmir	Not mentioned	Local men and women	Data was gathered through interviews but lacked useful information on the type of interview. Data was not subjected to analysis using any ethnobotanical index.
Khan et al. [41]	13	No	Yes	No	Deosai Plateau	Shina	Local experts (both men and women)	Data and related information were collected through semi-structured questionnaires. Data was analyzed using use value (UV) and relative citation frequency (RFCs).
Khattak et al. [42]	46	No	Yes	Yes	Karak District	Pashto	Elders (male and females)	Data was gathered through semi-structured questionnaires. The data obtained were quantitatively analyzed using use value (UV).
Khuroo et al. [43]	24	No	Yes	Yes	Kashmir Himalaya	Not mentioned	Traditional healers	Data was gathered through interviews but lacked useful information on the type of interview. Data was not subjected to analysis using any ethnobotanical index.
Mirani et al. [44]	22	No	Yes	Yes	Tharparkar	Not mentioned	Farmers	Data was collected through semi-structured open-ended interviews, observations, focus group discussions through participatory rural appraisal (PRA). Data was not subjected to analysis using any ethnobotanical index.
Mirani et al. [123]	35	No	Yes	Yes	Tharparkar	Not mentioned	Cattle farmers	Data was collected through semi-structured open-ended interviews, observations, focus group discussions through participatory rural appraisal (PRA). Data was not subjected to analysis using any ethnobotanical index.
Muhammad et al. [45]	22	No	Yes	Yes	Faisalabad	Not mentioned	Owners of pneumatic-cart pulling camels	Data was collected through a questionnaire but lacked useful information on its nature. Data was not subjected to analysis using any ethnobotanical index.
Mussarat et al. [46]	43	Yes	Yes	Yes	Indus River	Saraiki	Community members (male and female)	Semi-structured questionnaires were used for data collection. Informant consensus and fidelity level as well as direct matrix ranking were used to analyze the data.
Raza et al. [47]	64	Yes	Yes	Yes	Cholistan Desert	Saraiki	Livestock farmers and livestock healers	Structured questionnaire was used to collect data. Data was not subjected to analysis using any ethnobotanical index.
Raziq et al. [48]	8	No	Yes	Yes	Sulaiman Mountain	Not mentioned	Camel healers and healers	Data was collected through interviews but lacked useful information on its nature. Data was not subjected to analysis using any ethnobotanical index.
Shah et al. [49]	54	Yes	Yes	Yes	District Abbottabad	Not mentioned	Traditional healers, women, and herdsmen	Data was collected through interviews but lacked useful information on its nature. Data was not subjected to analysis using any ethnobotanical index.
Sher et al. [50]	29	No	Yes	Yes	District Swat	Pashto	Males	Semi-structured questionnaire was used to collect the data. Data was not analyzed using any ethnobotanical index.
Sindhu et al. [51]	35	No	Yes	Yes	District Mansehra	Not mentioned	Veterinarians, local healers, and farmers	Data was collected through interviews but lacked useful information on its nature. Data was not subjected to analysis using any ethnobotanical index.
Sindhu et al. [52]	35	No	Yes	Yes	District Jhang	Urdu	Veterinarians and local communities	Data was collected through interviews but lacked useful information on its nature. Data was not subjected to analysis using any ethnobotanical index.
Tariq et al. [53]	41	Yes	Yes	Yes	Kohat	Hindko	Local farmers and nomadic people	Semi-structured questionnaires were used for data collection. Informant consensus and fidelity level were used to analyze the data.
Tariq et al. [54]	24	No	Yes	Yes	Hangu region	Pashto	Farmers and migrants (Afghan refugees)	Data was collected through semi-structured questionnaires and analyzed through informant consensus and fidelity level.
ul Islam et al. [55]	28	No	Yes	Yes	Malakand Valley	Not mentioned	Local communities men and women	Data was collected through semi-structured questionnaires and analyzed through direct matrix ranking (DMR).
Ullah et al. [56]	60	No	Yes	No	District Charsadda	Pashto	Local peoples including farmers	The methodological framework is ambiguous with no clear indication of used questionnaires or interviews. Data was not subjected to analysis using any ethnobotanical index.
Yousafzai et al. [57]	49	No	Yes	Yes	Marghazar Valley, District Swat	Not mentioned	Males and females	The methodological framework is ambiguous with no clear indication of used questionnaires or interviews. Data was not subjected to analysis using any ethnobotanical index.

The dichotomy between Pathan and non-Pathan peoples has been an important trajectory in the cultural history of Pakistan. Pashto speaking people are referred to as *Pathans* in Pakistan, while they are mainly known also as *Pashtuns* or *Afghans* in the international literature. Pashto has been classified as an Eastern Iranian language which, according to MacKenzie [58], derived from the Aryan family of languages that “divided into its distinct Indian and Iranian branches more than three millennia ago.” In Pakistan, Pashto is spoken in the North Western districts. It is also spoken in Northeastern Baluchistan, and in Punjab it is still spoken in border areas of Mianwali and Attock. Additionally, the whole tribal area between Pakistan and Afghanistan is Pashto-speaking. Pashto is also one of the two most used official languages of Afghanistan. In Afghanistan, the Pashto-speaking area is in the East, South, and Southwest [59]. According to Gankovsky, the fundamentals of the original Pathan culture have evolved from the second millennium AD onwards [60]. The traditional life style of Pathans is encapsulated in *Pakhtunwali*, an orally transmitted customary code that includes vengeance (*Badal*), hospitality (*Melmastia*), and forgiveness (*Nanawati*), which has been remarkably described by the Norwegian anthropologist Fredrick Barth (1928-2016) [61].

## Results and discussion

### Herbal veterinary remedies of Pakistan

In Pakistan, hundreds of medicinal plants are used for treating livestock in remote areas where access to modern drugs is limited and people have sufficient knowledge about traditional therapies. The selected studies for the review indicated that a diversity of medicinal plants have remained popular among rural population across the country. Pakistani lay people, shepherds, farmers, nomadic grazers, and traditional healers used these medicinal plants to treat animal diseases and the selected studies recorded this traditional knowledge (Table 1). The current review reports 474 ethnoveterinary plants from different communities around the country that were used to treat domestic animals (Additional file 1: Table S2). Geographical distribution of the reported studies reveal that among the 35 studies, 14 studies were carried out in Khyber Pakhtunkhwa, 11 in Punjab, and four in Azad Kashmir, while Gilgit-Baltistan, Sindh, and Baluchistan were the least investigated areas in this regard with only three, two, and one studies, respectively (Table 1).

Researchers obtaining access to all regions of the country equally is a big hurdle to carrying out field studies. More specifically, the lack of ethnoveterinary literature from Baluchistan may be due to its restricted access to field researchers in the region. Living in the largest province of Pakistan, the people of Baluchistan are relatively more dependent on natural resources and keeping domestic animals in order to generate revenue in hard times [62]. More importantly, the remote communities of the province keep herds of domestic animals, and therefore the lack of literature does not mean that people residing in the region are not dependent on traditional therapies but rather that the difficult conditions which involve several factors including poor government policy prevented scientists from carrying out studies [63]. With regard to Sindh, the lack of reliable data on ethnoveterinary herbal remedies may involve some particular factors, obviously different from those of Baluchistan. Sindh region also plays an important role in agriculture and livestock production of the country. Sindh is comprised of plain areas and many big cities, and people living in remote and marginal areas keep animals to earn their livelihood, which can be seen in different spots on several occasions [64]. In general, people living in rural areas of inner Sindh are economically underprivileged and very few ethnoveterinary herbal studies have been conducted there.

**Fig. 1:**
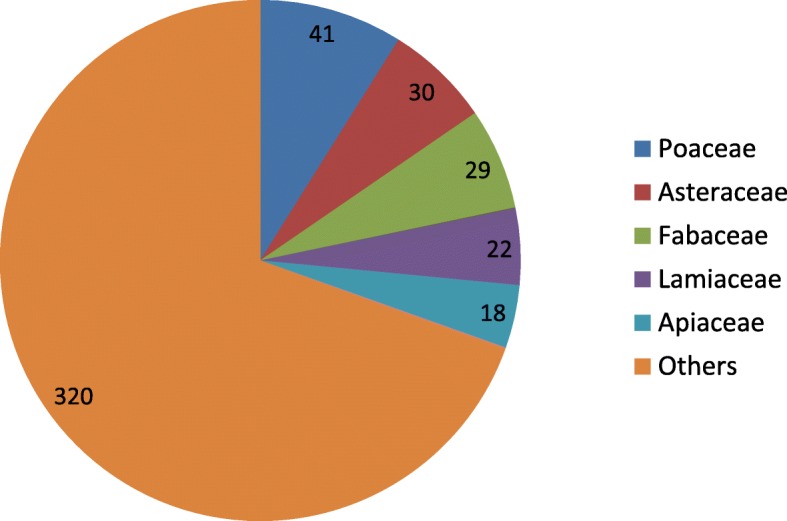
Overview of the most important botanical families used in the ethnoveterinary practices of Pakistan (number of recorded plant species)

**Fig. 2:**
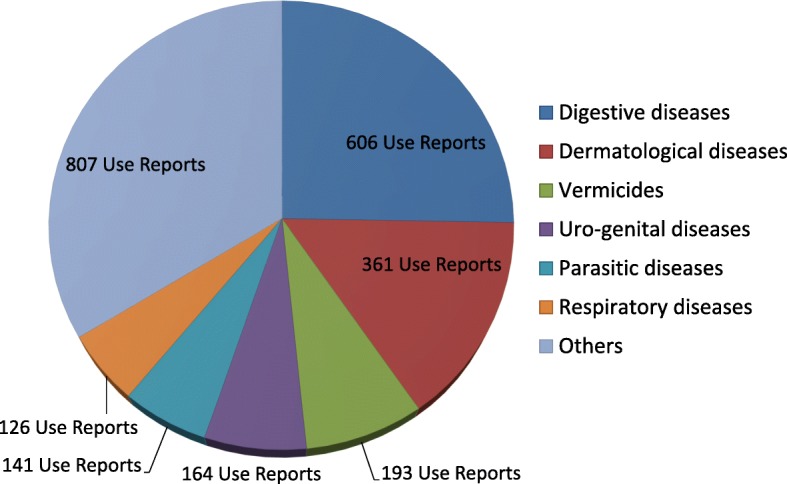
Most reported animal diseases/use categories in the Pakistani herbal ethnoveterinary practices

In this review, we recorded 2386 veterinary remedies (Additional file 1: Table S2, Fig. 1). Most remedies were prepared in the form of powder (Table 2). Frequently treated diseases included digestive and skin problems with 606 and 361 use reports, respectively (Fig. 2). The dominance of digestive problems as the main target of local herbal ethnoveterinary practices may be due to the lack of clean drinking water, unhygienic fodder consumption, and, most importantly, the fact that locals prefer to use herbs for minor animal complaints. It has been documented that poor fodder quality has significant negative impacts on animal health. As most parasitic diseases are concerned with the gastrointestinal tract, researchers have argued that nutrition status, pasture management, climatic conditions, animal immunity, and host preference are the major factors involved in the prevalence of different parasitic infections [65]. Grace et al. [66] reported that massive under-reporting, lack of veterinary surveillance activities, and few field-diagnostic facilities have been the possible causes of hindrance in in-depth establishing the true status of animal health in Pakistan.

**Table 2:** Preparations of the ethnoveterinary plant remedies of Pakistan

Preparation	Number of remedies
Ash	15
Juice	64
Concoction	9
Decoction	443
Extract	47
Fodder	388
Gum	3
Infusion	50
Latex	8
Oil	157
Paste	65
Poultice	13
Powder	730
Resin	6
Smoke	18

Descriptive statistics indicated that highest number of use reports was for *Brassica rapa* (86 use reports), *Foeniculum vulgare* (51), *Trachyspermum ammi* (50), *Allium cepa* (43), *Citrullus colocynthi*s (43), and *Melia azedarach* (35) (Additional file 1: Table S2). Some plants were recorded quite frequently, including *Brassica rapa* (23), *Foeniculum vulgare* (20 articles reported), *Allium cepa* (19), *Allium sativum* (18), *Melia azedarach* (17), *Citrullus colocynthis* (16), and *Ricinus communis* (15). The wide acceptance of these particular medicinal plants may not only be due to their efficient activity but also involve a few other factors like their large availability in markets and their possible long history of use in the traditional medicine practiced by healers, making their use more feasable than the use of plants which are difficult to harvest [67]. Moreover, the use of medicinal plants in a  given area is also shaped by the familiarity of local communities with their landscape, type of vegetation, seasonality, and ease of availability of herbal material [68].

The predominant botanical families were Poaceae (41 species; 144 use reports per family), Asteraceae (32; 107), and Fabaceae (29; 127) (Table 3, Fig. 1). These families have frequently been reported in a wide range of ethnobotanical studies [34, 69, 70]. The prevalence of the aforementioned botanical families shows that locals prefer to use plants growing in anthropogenic environments, i.e., plants that grow close to home gardens. Looking at the results obtained for each family in terms of use reports, it could be interpreted that the member plants of these families may have some specific effective pharmacologically active ingredients making them favorable in treating various ailments. The familiarity of local people with particular medicinal plants is also dependent upon their long-term perception based on continuous exposure to these natural resources. Furthermore, Pinaceae, Apiaceae, Poaceae, Brassicaceae, and Solanaceae families had the highest Use Values: 7.50, 6.35, 6.00, 5.71, and 5.31, respectively (Table 3).

**Table 3:** Use values of the botanical families reported in ethnoveterinary herbal practices in Pakistan

Botanical family	Number of recorded species	URs per family	Number of informants per family	UV_f_
Acanthaceae	2	9	7	1.29
Acoraceae	1	8	3	2.67
Agaricaceae	1	2	2	1.00
Aizoaceae	2	2	2	1.00
Amaranthaceae	12	40	13	3.08
Amaryllidaceae	4	78	24	3.25
Anacardiaceae	2	9	6	1.50
Apiaceae	18	165	26	6.35
Apocynaceae	11	55	17	3.24
Araceae	2	5	3	1.67
Araliaceae	1	2	2	1.00
Arecaceae	4	16	7	2.29
Asparagaceae	5	7	5	1.40
Asteraceae	32	107	25	4.28
Balsaminaceae	1	2	1	2.00
Berberidaceae	5	33	11	3.00
Betulaceae	2	3	2	1.50
Bignoniaceae	1	1	1	1.00
Bixaceae	1	1	1	1.00
Boraginaceae	7	21	9	2.33
Brassicaceae	7	137	24	5.71
Burseraceae	1	2	2	1.00
Buxaceae	1	1	1	1.00
Cactaceae	1	2	1	2.00
Cannabaceae	2	29	11	2.64
Capparaceae	3	26	13	2.00
Caprifoliaceae	4	8	5	1.60
Caryophyllaceae	3	3	3	1.00
Celastraceae	1	1	1	1.00
Cleomaceae	1	2	2	1.00
Commelinaceae	1	3	1	3.00
Convolvulaceae	3	24	12	2.00
Crassulaceae	2	6	3	2.00
Cucurbitaceae	7	56	18	3.11
Cupressaceae	1	1	1	1.00
Cyperaceae	2	5	3	1.67
Dioscoreaceae	1	1	1	1.00
Ebenaceae	1	1	1	1.00
Elaeagnaceae	1	2	1	2.00
Ephedraceae	1	1	1	1.00
Ericaceae	1	1	1	1.00
Euphorbiaceae	10	52	20	2.60
Fabaceae	29	127	27	4.70
Fagaceae	4	5	4	1.25
Gentianaceae	4	6	4	1.5
Geraniaceae	1	4	3	1.33
Gisekiaceae	1	1	1	1.00
Grossulariaceae	1	2	1	2.00
Hypericaceae	1	2	2	1.00
Iridaceae	1	1	1	1.00
Juglandaceae	1	4	3	1.33
Lamiaceae	22	120	25	4.80
Lauraceae	3	6	4	1.50
Liliaceae	2	1	1	1.00
Linaceae	1	10	5	2.00
Lythraceae	4	34	15	2.27
Malvaceae	15	32	12	2.67
Meliaceae	3	64	22	2.91
Menispermaceae	4	7	4	1.75
Molluginaceae	1	1	1	1.00
Moraceae	8	32	10	3.20
Musaceae	1	8	5	1.60
Myristicaceae	1	2	2	1.00
Myrtaceae	7	16	9	1.78
Nitrariaceae	1	19	9	2.11
Nyctaginaceae	3	6	4	1.50
Oleaceae	3	15	6	2.50
Orobanchaceae	1	1	1	1.00
Paeoniaceae	1	8	5	1.60
Papaveraceae	4	20	10	2.00
Pedaliaceae	2	8	5	1.60
Phyllanthaceae	4	11	3	3.67
Phytolaccaceae	1	1	1	1.00
Pinaceae	4	60	8	7.50
Piperaceae	2	18	9	2.00
Plantaginaceae	5	14	7	2.00
Platanaceae	1	2	2	1.00
Poaceae	41	144	24	6.00
Polygonaceae	14	63	17	3.7
Portulacaceae	1	1	1	1.00
Primulaceae	5	7	6	1.17
Pteridaceae	2	2	2	1.00
Putranjivaceae	1	1	1	1.00
Ranunculaceae	13	33	13	2.53
Rhamnaceae	4	24	12	2.00
Rosaceae	13	52	18	2.88
Rubiaceae	1	2	1	2.00
Rutaceae	6	42	20	2.10
Salicaceae	5	17	6	2.83
Salvadoraceae	2	5	4	1.25
Sapindaceae	2	15	6	2.50
Sapotaceae	1	2	2	1.00
Saxifragaceae	2	17	6	2.83
Scrophulariaceae	2	8	5	1.60
Simaroubaceae	2	6	4	1.50
Solanaceae	15	138	26	5.31
Tamaricaceae	1	12	8	1.50
Theaceae	1	19	8	2.38
Thymelaeaceae	4	17	6	2.83
Urticaceae	2	2	2	1.00
Verbenaceae	1	1	1	1.00
Violaceae	3	9	3	3.00
Vitaceae	1	3	3	1.00
Xanthorrhoeaceae	2	13	8	1.63
Zingiberaceae	5	65	17	3.82
Zygophyllaceae	5	19	8	2.38

The reviewed data confirmed that ethnoveterinary herbal remedies play a large role in Pakistan, especially in the most remote areas of the country; moreover, the remarkable heritage that emerges from the data reveals a robust link to very common pastoral activities and possibly also to the traditional pastoralist heriatge of most people inhabiting the rural areas [71].

### Little-known veterinary plants of Pakistan

Research on natural products is sometimes based on ethnobotanical information. One goal of ethnopharmacology is to improve our understanding of the pharmacological effects of traditionally used medicinal plants, especially for the benefts of rural remote communities that are highly marginalized and poverty stricken. In this section, we stress the need for the phytotherapeutical evaluations of those medicinal plants that have *rarely* been reported in the ethnoveterinary of Pakistan and have scarcely been investigated in previous pharmacological studies.

#### *Abutilon theophrasti*

The pharmacology of *Abutilon theophrasti* has rarely been investigated and only one potential and relevant study was found which indicated that methanolic extracts of the species have useful antifungal effects against selected fungal species [72]. Quercetin, an isolated flavonoid from the aqueous extract of *Abutilon theophrasti* seeds, has been reported to have a significant inhibitory effect on the growth of *Aspergillus niger* and *Fusarium* spp [118].

#### *Actaea spicata*

Researchers have claimed that trans-aconitic acid, isolated from ethanolic fractions of *Actaea spicata*, exhibits cytostatic action against Ehrlich’s ascites tumor [73]. Significant antioxidant activity of methanol extract and ethyl acetate fraction has been recorded for the species [74]. Similarly, its petroleum, ether, chloroform, methanol, and water extracts have exhibited antidepressant activity in experimental mice [75].

#### Aizoon canariense

Looking at the pharmacological activities of different extracts of *Aizoon canariense*, it has been mentioned that they exhibit moderate scavenging activity, as well as antibacterial, antifungal [76], and antioxidant activity [77].

#### Anagallis arvensis

Various extracts of *Anagallis arvensis* have shown strong antifungal activity [78–81]. The plant has antimicrobial, anti-inflammatory, and antioxidant effects [79], and molluscicidal activity [82]. Strong molluscicidal activity was found for its saponins, namely desglucoanagalloside B and anagalloside B [82]. An acetyl saponin isolated from the plant was found to possess marked taenicide activity [83]. Triterpene saponins exhibit oestrogenic activity [84]. Moroever, strong antifungal and antiviral activities of *Anagallis* saponins were also demonstrated [119–121].

#### Angelica glauca

Previous studies revealed that butylidene phthalide, derived from *Angelica glauca*, possesses antispasmodic activity [85]. Irshad et al. [86] reported that the essential oil (EO) of *Angelica glauca* exhibits good radical scavenging and peroxidation inhibition activities, and showed appreciable antimicrobial activity. Sharma et al. [87] reported that the EO of *Angelica glauca* exhibited broncho-relaxant activity against histamine and ovalbumin-induced broncho constriction in guinea pigs.

#### *Buddleja asiatica*

Buddleja asiaticaextracts have shown antimicrobial, antioxidant [88–90], antihepatotoxic [91], antispasmodic, and Ca^++^ antagonist activity [88].

#### Cocculus hirsutus

A variety of pharmacological activities have been exhibited by different extracts of *Cocculus hirsutus*, such as anti-diabetic activity [92], antihyperglycemic activity [93], anti-inflammatory and analgesic effects [94, 95], antimicrobial activity [96], diuretic activity, and laxative effects in different experiments [97]. The plant is well documented as a spermatogenic [92].

#### Cochlospermum religiosum

The bioactive secondary metabolite myricetin was isolated from in vivo and in vitro tissue samples of *Cochlospermum religiosum*. Myricetin is a naturally occurring flavonol found in many plants, having a wide array of biochemical properties, such as antineoplastic and anti-carcinogenic antioxidant activity, and also anti-inflammatory effects [98]. The plant is well documented for its antimicrobial potential by a number of researchers [99, 100] as well as for its immunomodulatory effects [101]. Ethanolic extract of *Cochlospermum religiosum* yielded promising results with respect to hepatoprotective activity [102]. Isorhamnetin, a flavonoid glycoside isolated from the plant, exhibited an antioxidant effect [103, 104].

#### Cynanchum viminale

One study has demonstrated the effect of the aqueous extract of *Cynanchum viminale* leaves as an analgesic, anti-inflammatory, and antipyretic in albino mice, which justify the traditional use of this plant [105].

#### Debregeasia saeneb

A study has demonstrated that *Debregeasia saeneb* exhibits potential anticancer activity [106].

#### Dichanthium annulatum

This plant has good anticancer effects [107]. Results revealed that the aerial parts of *Dichanthium annulatum* possess good antioxidant and antimicrobial activity [108].

#### Flueggea leucopyrus

The extract of this plant possesses significant antioxidant activity [109, 110] and antiproliferative properties, and it induced apoptosis in HEp-2 cells [110]. Ethanol extract of *Flueggea leucopyrus* leaves increases sexual behavior in rats, supporting its use as an aphrodisiac [111]. 

#### Litsea monopetala

Different extracts of *Litsea monopetala* exhibit anticancer properties [112] and antioxidant activity [113, 114].

#### Silene villosa

The methanolic extract of *Silene villosa* plays a protective role, while the alcoholic extract is active against CCl_4_-induced cardiac and renal toxicity in rats [115]. The alcoholic extract of the plant has also shown anti-inflammatory, wound healing, and hepatoprotective properties [116], as well as a cytotoxic effect [117].

After comprehensive bio-medical review of the recorded species, some medicinal plants reported in Table S2 were found to have not been pharmacologically in-depth investigated, including *Agrostis gigantea*, *Allardia tomentosa*, *Aristida adscensionis*, *Bothriochloa bladhii*, *Dactylis glomerata*, *Dracocephalum nuristanicum*, *Launaea nudicaulis*, *Sibbaldianthe bifurca*, *Spiraea altaica*, and *Thalictrum foetidum.* Lack of substantial research on these and other little-known veterinary plants indicates that robust effort is still needed to fill the gaps existing between the inputs arising from the ethnobotanical data and the actual body of phytopharmacological knowledge.

### Cross-cultural comparison

Ethnoveterinary herbal data was subjected to cross cultural comparison via Venn diagrams. In order to assess the effect of ethnicity in shaping ethnoveterinary practices, we compared the ethnoveterinary plants used by Pathan and non-Pathan ethnic communities across the country.

As described in previous paragraphs, Pathans populate the North-West of Pakistan and their human ecology has been particularly characterized by a very prominent pastoralist trajectory [122].

**Fig. 3:**
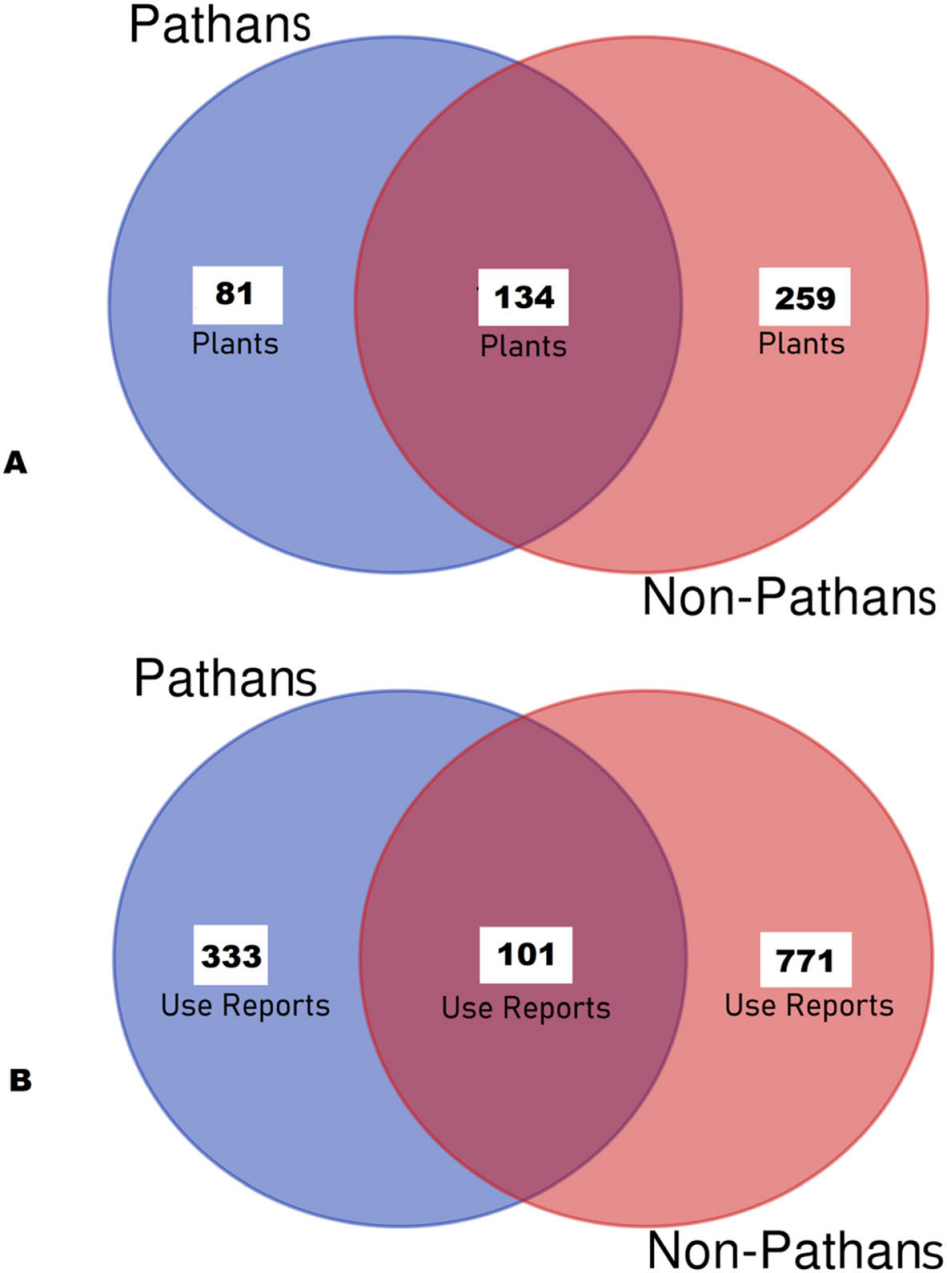
Venn diagrams showing the comparison of *ethnoveterinary plants* and *ethnoveterinary plant use reports* between Pakistani Pathans and non-Pathans

Cross-cultural comparative assessment indicated that approximately only one-third (134 plants; 28.3%) of the 474 medicinal plants were commonly used by both Pathans and non-Pathans (Fig. 3). Moreover, we considered the medicinal plants quoted by both Pathans and non-Pathans for further comparison in terms of their *use reports* and we found 101 (8%), out of 1205 total, which were shared by both groups (Fig. 3). This figure demonstrates that veterinary plants which are used by both groups are prepared and used in very different ways. The small overlap of the aforementioned use reports may suggest that detailed practices and experiences of local communities with their plants are largely divergent. The pattern of the above results might be also related to the epidemiological issues which cannot be ignored as every region has particular socio-environmental conditions which may affect the prevalence of specific animal diseases leading to certain medicinal plant uses instead of others.

### Cross-regional comparison

**Fig. 4:**
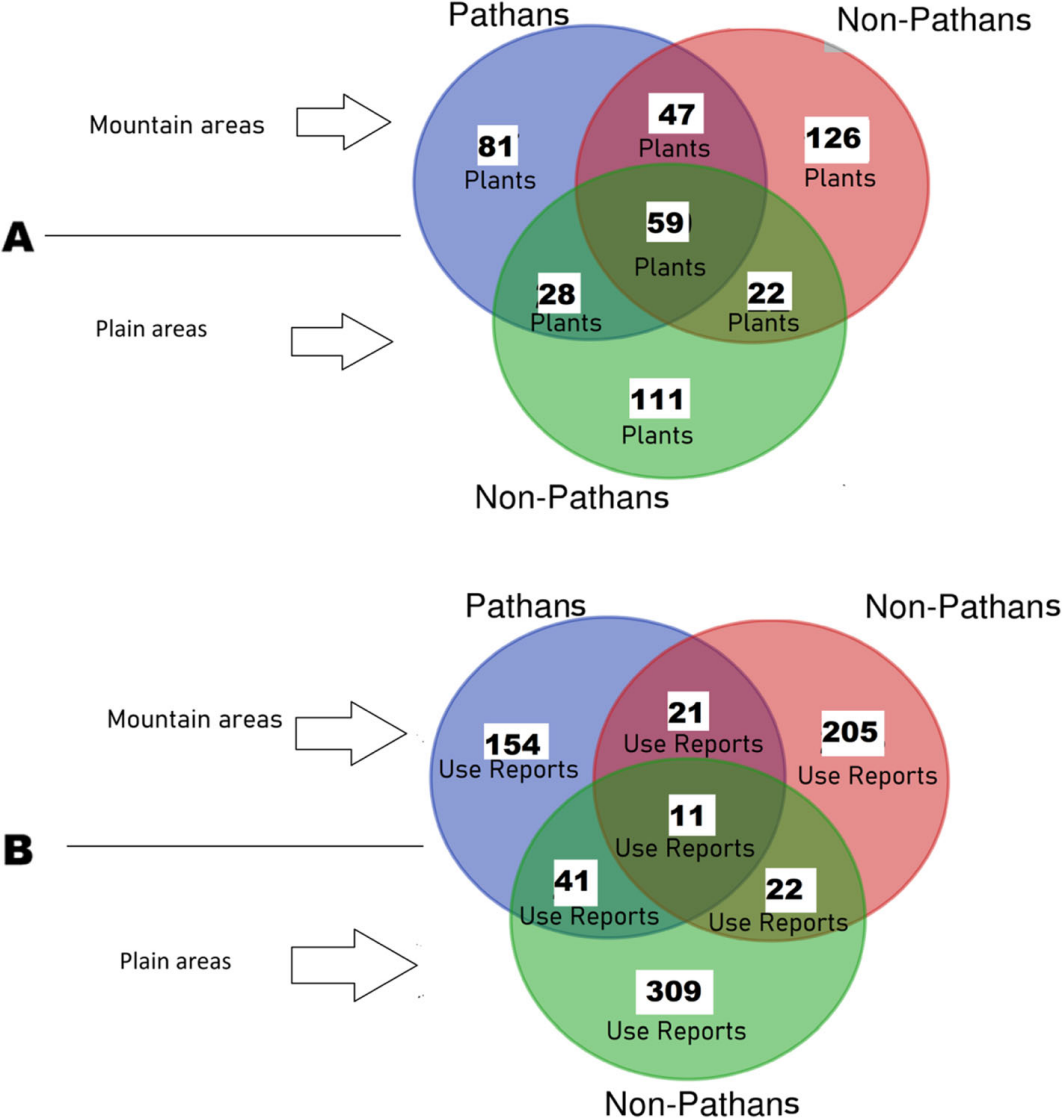
Venn diagrams showing the comparison of **a***ethnoveterinary plants* and **b***ethnoveterinary plant Use Reports* of mountain and plain areas and Pathan and non-Pathan groups in Pakistan. Venn diagram B refers only to 59 taxa used by all three clusters presented in Venn diagram A

In order to evaluate how geographical and ecological factors may have affected indigenous practices, the recorded data were subjected to regional comparison. Ethnoveterinary data was divided into three clusters, namely plants used by Pathan groups in mountain areas (a), plants used by non-Pathan groups in mountain areas (b), and plants used by non-Pathan groups in plain areas (c), and then the three clusters were represented via Venn diagrams (Fig. 4). Figure 4 shows (A) the overalap of the overall recorded veterinary plants and (B) the overlap of those Use Reports which refer only to those species that were reported by all three groups (59 taxa, 12.4% of the overall 474 medicinal plants, Fig. 4A). Only 11 out of the 763 plant Use Reports (1.4%) were shared among the three aforementioned groups. Figure 4B suggests that even those plants that were recorded among all three groups have actually within each cluster very different, probably *locally situated*, veterinary uses. Regional comparative analysis shows also that only 74 (9.7%) out of 763 plant reports are shared by local communities living in the mountain and in plain areas. These findings indicate that the divergences of actual plant utilizations are remarkable and that the difference between the plants used by Pathans and non-Pathans is somehow similar to the difference between plants used in plain and mountain areas. This pattern suggests that both geography/ecology and ethnicity/cultural customs have played a crucial role in shaping folk veterinary herbal knowledge in Pakistan.

## Conclusion

The current review revealed that in Pakistan, local communities have used and are possibly still using hundreds of medicinal plants to treat their domestic animals for generations. This remarkable cultural heritage may be linked to the pastoralist origin of many populations inhabiting the country, among which the Pathans living in the North-West of the country. Cross-cultural comparison showed that Pathan and non-Pathan ethnic communities share approximately one-third only of the medicinal plants and only 8% of the use reports (referred to the most commonly quoted plants). Cross-regional comparative analysis showed instead that only 12% of the overall quoted veterinary plants were shared between mountain and plain areas, suggesting that both ecological and cultural factors have played a role in shaping this remarkable veterinary heritage.

Furthermore, the literature review indicated that there are still some medicinal plants, as reported here, that need detailed phytochemical and pharmacological study to investigate their exact phytotherapeutical profiles. The promotion of the recorded ethnoveterinary heritage by stakeholders involved in rural development projects may be essential for improving animal well-being and presumably the quality of animal food products as well.

## Supplementary information

**Additional file 1: Table S2**. Medicinal plants used in ethnoveterinary practices in Pakistan.

## Data Availability

All the data can be found in the article.
