# Supplementary material for: Ethnoveterinary plants of Pakistan: a review
Source: J Ethnobiol Ethnomed. 2020 May 15;16:25. doi: 10.1186/s13002-020-00369-1 (PMC7227227; doi:10.1186/s13002-020-00369-1)
Supplement: Supplementary file 1 — Additional file 1: Table S2. Medicinal plants used in ethnoveterinary practices in Pakistan. [file 13002_2020_369_MOESM1_ESM.pdf]

Table S2. Medicinal plants used in ethnoveterinary practices of Pakistan.

**Plant taxon**	**Number of informants**	**Use Report**	**Use Value**	**Botanical family**	**Recorded veterinary use or cured diseases**	**Used parts**	**Preparation**	**Reference**
Abelmoschus ficulneus (L.) Wight & Arn.	1	1	1.00	Malvaceae	Scorpion bite	Whole plant	Powder	Ullah et al., 2017
Abutilon theophrasti Medik.	1	1	1.00	Malvaceae	Fever	Roots	Decoction	Khan and Hanif, 2006
Acacia modesta Wall.	1	2	2.00	Fabaceae	Tonic	Fruits	Raw	Aziz et al., 2018
					Easy delivery	Barks	Decoction	Khan and Hanif, 2006
					Expulsion of placenta	Barks	Decoction	Khan and Hanif, 2006
					Intestinal worm	Barks	Decoction	Khan et al., 2012
					Easy delivery	Barks	Decoction	Khan et al., 2012
					Placenta expulsion	Barks	Decoction	Khan et al., 2012
					Galactagogue	Fruits, Barks		Khattak et al., 2015
					Rheumatism	Fruits, Barks		Khattak et al., 2015
					Sex tonic	Fruits, Barks		Khattak et al., 2015
					Delivery	Leaves, Seeds	Decoction	Tariq et al., 2014
Acacia nilotica (L.) Delile	6	9	1.50	Fabaceae	Stomach disorders	Barks	Decoction	Abbasi et al., 2013
					Diarrhea	Leaves	Raw	Badar et al., 2017
					Worm infestation	Leaves	Raw	Badar et al., 2017

					Foot diseases	Barks	Decoction	Deeba et al., 2009
					Mouth diseases	Barks	Decoction	Deeba et al., 2009
					Genital prolapse	Barks	Powder, Decoction	Dilshad et al., 2008
					Dysentary	Fruits	Juice	Khattak et al., 2015
					Astringent	Leaves	Powder	Khattak et al., 2015
					Ulcers	Leaves	Powder	Khattak et al., 2015
					Infertility	Pods	Powder	Raza et al., 2014
Achyranthes aspera L.	3	6	2.00	Amaranthaceae	Ectoparasites	Flowers	Raw	Khan and Hanif, 2006
					Ectoparasites	Leaves	Powder	Khan and Hanif, 2006
					Lochia after parturition	Roots	Extract	Khan, 2009
					Myiasis	Leaves	Paste	Khan, 2009
					Anthelmintic	Flowers		Khan, 2009
					Placenta expulsion	Roots	Extract	Khan, 2009
					Wound	Leaves	Raw	Khan, 2009
					Anthelmintic	Whole plant	Powder	Tariq et al., 2014
					Easy delivery	Whole plant	Powder	Tariq et al., 2014
Aconitum heterophyllum Wall. ex Royle	1	5	5.00	Ranunculaceae	Abdominal pain	Roots, Flowers		Khan et al., 2015b
					Diabetes	Roots, Flowers		Khan et al., 2015b
					Digestive disorders	Roots, Flowers		Khan et al., 2015b
					Dysentery	Roots, Flowers		Khan et al., 2015b
					Fever	Roots,		Khan et al., 2015b

						Flowers		
Aconitum laeve Royle	1	5	5.00	Ranunculaceae	Delivery	Rhizomes	Extract	Khuroo et al., 2007
					Diarrhea	Rhizomes	Extract	Khuroo et al., 2007
					Internal body infection	Rhizomes	Extract	Khuroo et al., 2007
					Liver problems	Rhizomes	Extract	Khuroo et al., 2007
					Stomach ailments	Rhizomes		Khuroo et al., 2007
Aconitum napellus L.	1	1	1.00	Ranunculaceae	Wound	Flowers, Leaves	Raw	Ali et al., 2017
Aconitum violaceum Jacquem. ex Stapf	1	1	1.00	Ranunculaceae	Respiratory	Rhizomes	Powder	Shah et al., 2012
Acorus calamus L.	3	8	2.67	Acoraceae	Colic	Rhizomes	Powder	Khan et al., 2015a
					Gutoo	Rhizomes	Powder	Khan et al., 2015a
					Black quarter	Rhizomes	Powder	Khan et al., 2015a
					Increase milk	Rhizomes	Powder	Khan et al., 2015a
					Increase body temperature	Rhizomes	Powder	Ul Hassan et al., 2014
					Colic	Rhizomes	Powder	Yousfzai et al., 2010
					Common cold	Rhizomes	Powder	Yousfzai et al., 2010
					Cough	Rhizomes	Powder	Yousfzai et al., 2010
Actaea spicata L.	1	2	2.00	Ranunculaceae	Asthma	Rhizomes	Powder, Raw	Khuroo et al., 2007

					Stomach worms	Rhizomes	Powder, Raw	Khuroo et al., 2007
Adiantum incisum Forssk.	1	1	1.00	Pteridaceae	Abdominal pain	Leaves	Powder	Abbasi et al., 2013
Adiantum venustum D. Don	1	1	1.00	Pteridaceae	Chronic tumors	Whole plant	Powder	Khan and Hanif, 2006
Aerva javanica (Burm.f.) Juss. ex Schult.	4	5	1.25	Amaranthaceae	Skin infection	Roots	Decoction	Abbasi et al., 2013
					Fly infestation	Leaves	Smoke	Farooq et al., 2008
					Diarrhea	Inflorescence, Seeds		Khan, 2009
					Haematuria	Flowers, Seeds		Khan, 2009
					Laxative	Aerial parts	Decoction	Raza et al., 2014
Aesculus indica (Wall. ex Cambess.) Hook.	6	9	1.50	Sapindaceae	Abdominal pain	Fruits	Powder	Abbasi et al., 2013
					Cough	Fruits	Powder	Abbasi et al., 2013
					Fever	Fruits	Powder	Abbasi et al., 2013
					Stimulant	Fruits	Powder	Ahmed and Murtaza, 2015
					Chest diseases	Fruits		Khan et al., 2012
					Colic	Seeds	Powder	Shah et al., 2012
					Stomach problems	Seeds	Powder	Shah et al., 2012
					Colic	Fruits		Sher et al., 2004
					Colic	Fruits	Powder	Yousfzai et al., 2010
Agaricus campestris L.	2	2	1.00	Agaricaceae	Fever	Whole plant	Juice	Khan and Hanif, 2006
					Fever	Whole plant	Raw	Khan et al., 2015a
Agrostis gigantea Roth	1	1	1.00	Poaceae	Allergy	Leaves		Harun et al., 2017
Ailanthus altissima (Mill.)	3	5	1.67	Simaroubaceae	Galactagogue	Leaves	Raw	Aziz et al., 2018

Swingle					Kill the flea	Leaves, Resin		Islam et al., 2012
					Kill the ticks	Leaves, Resin		Islam et al., 2012
					Wound	Leaves, Resin		Islam et al., 2012
					Purgative	Leaves	Raw	Shah et al., 2012
Ailanthus excelsa Roxb.	1	1	1.00	Simaroubaceae	Malarial fever	Leaves	Decoction	Khan, 2009
Aizoon canariense L.	1	1	1.00	Aizoaceae	Myiasis	Aerial parts	Powder	Farooq et al., 2008
Ajuga integrifolia Buch.-Ham.	4	8	2.00	Lamiaceae	Abdominal pain	Whole plant	Powder	Abbasi et al., 2013
					Blood purification	Leaves	Decoction	Aziz et al., 2018
					Skin problems	Leaves	Decoction	Aziz et al., 2018
					Vermicides	Leaves	Decoction	Aziz et al., 2018
					Fever	Whole plant	Decoction	Khan and Hanif, 2006
					Haemorrhagic diseases	Whole plant	Juice	Yousfzai et al., 2010
					Septicaemia	Whole plant	Juice	Yousfzai et al., 2010
Ajuga parviflora Benth.	1	3	3.00	Lamiaceae	Sore	Aerial parts	Powder	Khuroo et al., 2007
					Swelling	Aerial parts	Powder	Khuroo et al., 2007
					Wound	Aerial parts	Powder	Khuroo et al., 2007
Albizia lebbeck (L.) Benth.	3	5	1.67	Fabaceae	Helminthes	Leaves	Powder, Raw	Hussain et al., 2008
					Diarrhea	Barks	Decoction	Khan and Hanif, 2006

					Eye diseases	Latex		Khattak et al., 2015
					Lice infestation	Barks	Juice	Khattak et al., 2015
					Dysentary	Barks	Decoction	Khan and Hanif, 2006
					Snake bite	Seeds	Powder	Khan and Hanif, 2006
					Eye diseases	Leaves	Decoction	Mussarat et al., 2014
Alhagi maurorum Medik.	3	4	1.33	Fabaceae	Constipation	Leaves	Decoction	Khan, 2009
					Digestive disorders	Leaves	Decoction	Raza et al., 2014
					Indigestion	Leaves	Decoction	Raza et al., 2014
					Gastrointestinal helminthiasis	Seeds	Powder	Sindhu et al., 2010
Allardia tomentosa Decne.	1	1	1.00	Asteraceae	Intestinal problems	Leaves	Powder	Ali et al., 2017
Allium cepa L.	19	46	2.42	Amaryllidaceae	Fever	Bulbs	Powder	Abbasi et al., 2013
					Galactagogue	Bulbs	Powder	Abbasi et al., 2013
					Indigestion	Bulbs	Powder	Abbasi et al., 2013
					Stomach disorders	Bulbs	Powder	Abbasi et al., 2013
					Bloat	Bulbs	Raw	Ali et al., 2017
					Tympany	Bulbs	Raw	Ali et al., 2017
					Wound	Bulbs	Decoction	Ali et al., 2017
					Galactagogue	Bulbs	Powder	Aziz et al., 2018
					Anorexia	Fruits	Powder	Badar et al., 2017
					Bleeding after birth	Bulbs	Powder	Badar et al., 2017
					Indigestion	Bulbs	Powder	Badar et al., 2017
					Anorexia	Bulbs	Powder	Deeba et al., 2009
					Cough	Bulbs	Powder	Deeba et al., 2009
					Fever	Bulbs	Powder	Deeba et al., 2009
					Helminthes	Bulbs	Powder	Hussain et al., 2008

					Flatulence	Bulbs	Soup	Khan and Hanif, 2006
					Indigestion	Bulbs	Soup	Khan and Hanif, 2006
					Colic	Bulbs	Raw	Khan et al., 2015a
					Dirrrhea	Bulbs	Raw	Khan et al., 2015a
					Mastitis	Bulbs	Raw	Khan et al., 2015a
					Vaginal prolapse	Bulbs	Raw	Khan et al., 2015a
					Stimulate estrus cycle	Bulbs	Raw	Khuroo et al., 2007
					Foot diseases	Bulbs	Raw	Mirani et al., 2014
					Mastitis	Bulbs	Raw	Mirani et al., 2014
					Mouth diseases	Bulbs	Raw	Mirani et al., 2014
					Placenta expulsion	Bulbs	Raw	Mirani et al., 2014
					Foot diseases	Bulbs	Raw	Mirani et al., 2016
					Mastitis	Bulbs	Raw	Mirani et al., 2016
					Mouth diseases	Bulbs	Raw	Mirani et al., 2016
					Placenta expulsion	Bulbs	Raw	Mirani et al., 2016
					Halitosis	Bulbs		Muhammad et al., 2005
					Indigestion	Bulbs		Muhammad et al., 2005
					Mastitis	Bulbs	Raw	Mussarat et al., 2014
					Digestive disorders	Bulbs	Raw	Raza et al., 2014
					Indigestion	Bulbs	Raw	Raza et al., 2014
					Ectoparasites	Bulbs	Powder	Shah et al., 2012
					Fever	Bulbs	Powder	Shah et al., 2012
					Wound	Tubers,	Powder	Shah et al., 2012

						Seeds		
					Mange	Bulbs	Raw	Sindhu et al., 2012
					Ectoparasites	Leaves	Powder	Tariq et al., 2016
					Abdominal pain	Bulbs		Ul Hassan et al., 2014
					Eczema	Bulbs		Ullah et al., 2017
					Foot diseases	Bulbs		Ullah et al., 2017
					Indigestion	Bulbs		Ullah et al., 2017
					Mouth diseases	Bulbs		Ullah et al., 2017
					Scabies	Bulbs		Ullah et al., 2017
Allium jacquemontii Kunth	1	1	1.00	Amaryllidaceae	Unequal mammary glands	Bulbs	Powder	Abbasi et al., 2013
Allium sativum L.	19	31	1.63	Amaryllidaceae	Bloat	Bulbs	Raw	Ali et al., 2017
					Genital prolapse	Bulbs	Powder	Aziz et al., 2018
					Anorexia	Bulbs	Powder	Deeba et al., 2009
					Mastitis	Bulbs	Decoction	Deeba et al., 2009
					Genital prolapse	Bulbs	Raw	Dilshad et al., 2008
					Mastitis	Bulbs	Powder	Dilshad et al., 2010
					Helminthes	Bulbs	Powder	Hussain et al., 2008
					Stomachic problems	Stems, Leaves		Islam et al., 2012
					Foot diseases	Bulbs	Raw	Khan et al., 2015a
					Mastitis	Bulbs	Raw	Khan et al., 2015a
					Mouth diseases	Bulbs	Raw	Khan et al., 2015a
					Off feeding	Bulbs	Raw	Khan et al., 2015a
					Placenta expulsion	Bulbs	Raw	Mirani et al., 2014
					Placenta expulsion	Bulbs	Raw	Mirani et al., 2016
					Respiratory diseases	Bulbs	Raw	Mirani et al., 2016

					Halitosis	Bulbs		Muhammad et al., 2005
					Indigestion	Bulbs		Muhammad et al., 2005
					Diarrhea	Bulbs	Decoction	Mussarat et al., 2014
					Mastitis	Bulbs	Powder	Mussarat et al., 2014
					Digestive disorders	Bulbs	Raw	Raza et al., 2014
					Indigestion	Bulbs	Raw	Raza et al., 2014
					Maggots from wound	Tubers, Seeds	Powder	Shah et al., 2012
					Gastrointestinal disorders	Bulb	Powder	Sher et al., 2004
					Gastrointestinal helminthiasis	Bulbs	Powder	Sindhu et al., 2010
					Tick infestation	Bulbs	Powder	Sindhu et al., 2010
					Helminthiasis	Bulbs	Raw	Sindhu et al., 2012
					Febrifuge	Whole plant	Decoction	Tariq et al., 2014
					Gastric ailments	Stems	Concoction	Tariq et al., 2014
					Tonic	Whole plant	Decoction	Tariq et al., 2014
					Fertility agent	Rhizome s	Paste	Ul Hassan et al., 2014
					Anorexia	Bulbs	Raw	Yousfzai et al., 2010
Alnus nitida (Spach) Endl.	1	2	2.00	Betulaceae	Foot diseases	Leaves	Powder	Khuroo et al., 2007
					Mouth diseases	Leaves	Powder	Khuroo et al., 2007
Aloe vera (L.) Burm.f.	8	12	1.50	Xanthorrhoeace ae	Digestive disorders	Leaves	Powder	Abbasi et al., 2013
					Mouth diseases	Leaves	Powder	Badar et al., 2017
					Mouth diseases	Leaves	Powder	Badar et al., 2017
					Toxaemia	Leaves	Powder	Badar et al., 2017

					Skin problems	Latex		Khattak et al., 2015
					Placenta expulsion	Leaves	Latex	Mirani et al., 2016
					Scabies	Leaves	Raw	Mussarat et al., 2014
					Stomach problems	Leaves	Raw	Mussarat et al., 2014
					Helminthiasis	Stems	Powder	Sindhu et al., 2012
					Mange	Leaves	Powder	Sindhu et al., 2012
					Gastric ailments	Roots	Powder	Tariq et al., 2014
					Gastric ailments	Leaves	Decoction	Tariq et al., 2016
Amaranthus tricolor L.	1	2	2.00	Amarnathaceae	Dysentery	Whole plant		Khan, 2009
					Indigestion	Whole plant		Khan, 2009
Amaranthus viridis L.	4	6	1.50	Amarnathaceae	Weakness	Whole plant	Raw	Abbasi et al., 2013
					Diuretic	Whole plant, Seeds	Powder	Aziz et al., 2018
					Tonic	Whole plant, Seeds	Powder	Aziz et al., 2018
					Constipation	Whole plant	Powder	Shah et al., 2012
					Purgative	Whole plant	Powder	Shah et al., 2012
					Malarial fever	Leaves, seeds	Decoction	Ahmed and Murtaza, 2015
Ammi visnaga (L.) Lam.	2	2	1.00	Apiaceae	Abdominal pain	Fruits	Decoction	Aziz et al., 2018
					Increase body temperature	Fruits	Decoction	Ul Hassan et al., 2014
Amomum subulatum Roxb.	8	21	2.63	Zingiberaceae	Anorexia	Seeds	Powder	Badar et al., 2017

					Galactagogue	Seeds	Powder	Badar et al., 2017
					Worm infestation	Seeds	Powder	Badar et al., 2017
					Anorexia	Leaves	Powder	Deeba et al., 2009
					Bloat	Leaves	Powder	Deeba et al., 2009
					Cough	Leaves	Powder	Deeba et al., 2009
					Diarrhea	Fruits	Raw	Deeba et al., 2009
					Fever	Leaves	Powder	Deeba et al., 2009
					Anestrus	Fruits	Powder	Dilshad et al., 2008
					Mastitis	Fruits		Dilshad et al., 2010
					Fever	Fruits	Powder	Khan et al., 2015a
					Mastitis	Fruits	Powder	Khan et al., 2015a
					Tympney	Fruits	Powder	Khan et al., 2015a
					Weakness	Fruits	Powder	Khan et al., 2015a
					Digestive disorders	Fruits		Muhammad et al., 2005
					Halitosis	Fruits		Muhammad et al., 2005
					Indigestion	Fruits		Muhammad et al., 2005
					Systemic disorders	Fruits		Muhammad et al., 2005
					Systemic disorders	Fruits		Muhammad et al., 2005
					Mastitis	Fruits	Powder	Mussarat et al., 2014
					Gastrointestinal helminthiasis	Fruits	Powder	Sindhu et al., 2010
Anagallis arvensis L.	2	3	1.50	Primulaceae	Mosquito bites	Whole palnt	Paste	Sher et al., 2004
					Antiparasitic	Whole palnt	decoction	Sher et al., 2004
					Cough	Whole	Powder	Tariq et al., 2016

						plant		
Anamirta cocculus (L.) Wight & Arn.	1	2	2.00	Menispermaceae	Fever	Leaves	Decoction	Khan, 2009
					Lice infestation	Fruits	Paste	Khan, 2009
Anethum graveolens L.	4	6	1.50	Apiaceae	Galactagogue	Seeds	Raw	Aziz et al., 2018
					Diarrhea	Seeds	Powder	Deeba et al., 2009
					Tympany	Seeds	Raw	Khan et al., 2015a
					Digestive disorders	Seeds		Muhammad et al., 2005
					Halitosis	Fruits		Muhammad et al., 2005
					Indigestion	Fruits		Muhammad et al., 2005
Anethum sowa Roxb. ex Fleming	1	1	1.00	Apiaceae	Galactagogue	Seeds	Raw	Abbasi et al., 2013
Angelica glauca Edgew.	2	2	1.00	Apiaceae	Cold fever	Whole plant	Decoction	Ahmed and Murtaza, 2015
					Galactagogue	Rhizomes	Powder	Khuroo et al., 2007
Apluda mutica L.	1	2	2.00	Poaceae	Antibacterial	Aerial parts		Harun et al., 2017
					Digestive disorders	Aerial parts		Harun et al., 2017
Aquilegia pubiflora Wall. ex Royle	1	1	1.00	Ranunculaceae	Anthelmintic	Aerial parts	Powder	Ahmed and Murtaza, 2015
Areca catechu L.	1	1	1.00	Arecaceae	Genital prolapse	Seeds	Powder	Dilshad et al., 2008
Argemone mexicana L.	1	3	3.00	Papaveraceae	Anthelmintic	Seeds	Oil	Khan, 2009
					Fever	Leaves	Juice	Khan, 2009
					Wound	Latex, Seeds	Latex, Oil	Khan, 2009

Arisaema flavum (Forssk.) Schott	3	4	1.33	Araceae	Mouth diseases	Leaves	Infusion	Ahmed and Murtaza, 2015
					Foot diseases	Leaves	Infusion	Ahmed and Murtaza, 2015
					Milk deficiency	Rhizome s	Decoction	Khan et al., 2012
					Myiasis	Roots	Powder	Sindhu et al., 2010
Arisaema jacquemontii Blume	3	11	3.66	Araceae	Cholera	Whole plant		Ahmed and Murtaza, 2015
					Flu	Whole plant		Ahmed and Murtaza, 2015
					Dysentery,	Whole plant		Ahmed and Murtaza, 2015
					Dyspepsia	Whole plant		Ahmed and Murtaza, 2015
					Snake bite	Whole plant		Ahmed and Murtaza, 2015
					Inflammation	Whole plant		Ahmed and Murtaza, 2015
					Cough	Rhizome	Decoction	Sher et al., 2004
					Respiratory tract infection	Rhizome	Decoction	Sher et al., 2004
					Common cold	Rhizome s	Powder	Yousfzai et al., 2010
					Cough	Rhizome s	Powder	Yousfzai et al., 2010
					Respiratory diseases	Rhizome s	Powder	Yousfzai et al., 2010
Aristida adscensionis L.	1	1	1.00	Poaceae	Itching	Aerial parts		Harun et al., 2017
Artemisia absinthium L.	2	9	4.50	Astaraceae	Anthelmintic	Whole plant	Powder	Aziz et al., 2018

					Anthelmentic	Leaves, Flowers, Stems		Islam et al., 2012
					Antiseptic	Leaves, Flowers, Stems		Islam et al., 2012
					Febrifuge	Leaves, Flowers, Stems		Islam et al., 2012
					Fever	Leaves, Flowers, Stems		Islam et al., 2012
					Gastric ailments	Leaves, Flowers, Stems		Islam et al., 2012
					Indigestion	Leaves, Flowers, Stems		Islam et al., 2012
					Stomachic	Leaves, Flowers, Stems		Islam et al., 2012
					Worm	Leaves, Flowers, Stems		Islam et al., 2012
Artemisia maritima L.	3	4	1.33	Astaraceae	Constipation	Leaves	Raw	Ali et al., 2017
					Indigestion	Leaves	Raw	Ali et al., 2017
					Wound	Aerial parts	Decoction	Raziq et al., 2010
					Worm	Whole plant	Powder	Shah et al., 2012
Artemisia santolinifolia Turcz. ex Krasch.	1	4	4.00	Astaraceae	Ectoparasites	Leaves, Stems	Paste	Ali et al., 2017
					Endoparasites	Leaves,	Paste	Ali et al., 2017

						Stems		
					Worms	Leaves, Stems	Paste	Ali et al., 2017
					Wound	Leaves, Stems	Paste	Ali et al., 2017
Artemisia scoparia Waldst. & Kitam.	1	3	3.00	Astaraceae	Blood purification	Leaves	Decoction	Aziz et al., 2018
					Gastric ailments	Leaves	Decoction	Aziz et al., 2018
					Skin irritation	Leaves	Decoction	Aziz et al., 2018
Artemisia sieversiana Ehrh.	1	1	1.00	Astaraceae	Pneumonia	Leaves	Raw	Ali et al., 2017
Artemisia vulgaris L.	1	2	2.00	Astaraceae	Anthelmintic	Whole plant	Powder	Shah et al., 2012
					Colic	Whole plant	Powder	Shah et al., 2012
Arundo donax L.	3	5	1.67	Poaceae	Worm infestation	Seeds	Raw	Badar et al., 2017
					Antiseptic	Leaves		Harun et al., 2017
					Diuretic	Leaves		Harun et al., 2017
					Diuretic	Leaves		Harun et al., 2017
					Gastrointestinal helminthiasis	Leaves	Powder	Sindhu et al., 2010
Asparagus adscendens Roxb.	1	1	1.00	Asparagaceae	Galactagogue	Leaves		Yousfzai et al., 2010
Asparagus asiaticus L.	1	1	1.00	Asparagaceae	Delivery	Roots	Concoction	Tariq et al., 2014
Asparagus filicinus Buch.-Ham. ex D.Don	1	1	1.00	Asparagaceae	Easy delivery	Seeds	Decoction	Khuroo et al., 2007
Asphodelus tenuifolius Cav.	1	1	1.00	Xanthorrhoeaceae	Constipation	Leaves	Raw	Abbasi et al., 2013
Astragalus bakaliensis Bunge	1	2	2.00	Fabaceae	Galactagogue	Whole plant	Raw	Khattak et al., 2015
					Tonic	Whole plant	Raw	Khattak et al., 2015

Astragalus zanskarensis Bunge	1	2	2.00	Fabaceae	Eye diseases	Stems, Leaves	Ash	Ali et al., 2017
					Wound	Stems, Leaves	Paste	Ali et al., 2017
Avena sativa L.	1	3	3.00	Poaceae	Cough	Seeds	Infusion	Ali et al., 2017
					Liver problems	Seeds	Infusion	Ali et al., 2017
					Pneumonia	Seeds	Infusion	Ali et al., 2017
Azadirachta indica A.Juss.	11	25	2.27	Meliaceae	Toxaemia	Leaves	Powder	Badar et al., 2017
					Worm infestation	Leaves	Powder	Badar et al., 2017
					Helminthiasis	Leaves	Decoction	Farooq et al., 2008
					Lice infestation	Leaves	Decoction	Farooq et al., 2008
					Myiasis	Leaves	Raw	Farooq et al., 2008
					Helminthes	Leaves	Extract	Hussain et al., 2008
					Fever	Leaves	Raw	Khan and Hanif, 2006
					Refrigerant	Leaves	Raw	Khan and Hanif, 2006
					Blood in milk	Fruits	Powder	Khan et al., 2015a
					Ectoparasites	Leaves	Decoction	Mirani et al., 2014
					Endoparasites	Leaves	Decoction	Mirani et al., 2014
					Myiasis	Leaves	Paste	Mirani et al., 2014
					Endoparasites	Leaves	Decoction	Mirani et al., 2016
					Endoparasites	Leaves	Decoction	Mirani et al., 2016
					Myiasis	Leaves	Powder	Mirani et al., 2016
					Uterine prolapse	Leaves	Decoction	Mirani et al., 2016
					Scabies	Leaves	Infusion	Mussarat et al., 2014
					Skin abscesses	Leaves	Infusion	Mussarat et al., 2014
					Helminthiasis	Leaves	Decoction	Raza et al., 2014

					Myiasis	Leaves	Decoction	Raza et al., 2014
					Wound	Leaves	Decoction	Raza et al., 2014
					Helminthiasis	Stems	Powder	Sindhu et al., 2012
					Mange	Leaves	Decoction	Sindhu et al., 2012
					Tick infestation	Leaves	Powder	Sindhu et al., 2012
					Wound	Leaves		Ullah et al., 2017
Baccharoides anthelmintica (L.) Moench	5	17	3.40	Asteraceae	Allergy	Seeds	Raw	Badar et al., 2017
					Genital prolapse	Seeds	Raw	Badar et al., 2017
					Inflammation	Seeds	Raw	Badar et al., 2017
					Mastitis	Seeds	Raw	Badar et al., 2017
					Toxaemia	Seeds	Raw	Badar et al., 2017
					Traumatic	Seeds	Raw	Badar et al., 2017
					Mastitis	Seeds	Raw	Deeba et al., 2009
					Mastitis	Seeds	Raw	Dilshad et al., 2010
					Digestive disorders	Seeds		Muhammad et al., 2005
					Halitosis			Muhammad et al., 2005
					Halitosis			Muhammad et al., 2005
					Indigestion			Muhammad et al., 2005
					Indigestion			Muhammad et al., 2005
					Respiratory	Seeds		Muhammad et al., 2005
					Systemic disorders	Seeds		Muhammad et al., 2005
					Mastitis	Seeds	Raw	Mussarat et al., 2014
					Stomach problems	Seeds	Raw	Mussarat et al., 2014
Balanites aegyptiaca (L.) Delile	1	4	4.00	Zygophyllaceae	Anthelmintic	Seeds		Khan, 2009

					Colic	Seeds		Khan, 2009
					Constipation	Seeds		Khan, 2009
					Purgative	Seeds		Khan, 2009
Bambusa bambos (L.) Voss	1	1	1.00	Poaceae	Helminthes	Leaves	Decoction	Hussain et al., 2008
Barleria prionitis L.	1	5	5.00	Acanthaceae	Abscess	Leaves	Paste	Khan, 2009
					Astringent	Leaves	Decoction	Khan, 2009
					Diarrhea	Leaves	Decoction	Khan, 2009
					Foot diseases	Leaves	Paste	Khan, 2009
					Mouth diseases	Leaves	Paste	Khan, 2009
Bauhinia variegata L.	1	2	2.00	Fabaceae	Constipation	Roots	Decoction	Khan and Hanif, 2006
					Diarrhea	Leaves, Flowers	Raw	Khan and Hanif, 2006
Berberis lycium Royle	10	21	2.10	Berberidaceae	Bone fracture	Barks	Decoction	Abbasi et al., 2013
					Wound	Barks	Decoction	Abbasi et al., 2013
					Mouth diseases	Roots	Decoction	Aziz et al., 2018
					Refrigerant	Roots	Decoction	Aziz et al., 2018
					Wound	Roots	Decoction	Aziz et al., 2018
					Analgesic	Barks		Islam et al., 2012
					Refrigerant	Barks		Islam et al., 2012
					Cough	Barks	Decoction	Khan et al., 2015a
					Antiseptic	Roots Barks	Powder	Shah et al., 2012
					Wound	Roots Barks	Powder	Shah et al., 2012
					Wound	Roots Barks	Powder	Shah et al., 2012
					Bone fracture	Roots, Fruits	Paste, Powder	Shedayi et al., 2014
					Diabetes	Roots,	Paste,	Shedayi et al., 2014

						Fruits	Powder	
					Pain of joint	Roots, Fruits	Paste, Powder	Shedayi et al., 2014
					Rheumatism	Roots, Fruits	Paste, Powder	Shedayi et al., 2014
					Wound	Roots, Fruits	Paste, Powder	Shedayi et al., 2014
					Digestive problmes	Roots	Powder	Sher et al., 2004
					Myiasis	Roots	Powder	Sindhu et al., 2010
					Internal injuries	Roots	Decoction	Ul Hassan et al., 2014
					Digestion	Roots Barks		Yousfzai et al., 2010
Berberis orthobotrys Bien. ex Aitch.	1	8	8.00	Berberidaceae	Bone fracture	Leaves, Fruits, Roots		Khan et al., 2015b
					Wound	Leaves, Fruits, Roots		Khan et al., 2015b
					Tumour	Leaves, Fruits, Roots		Khan et al., 2015b
					Ophthalmic diseases	Leaves, Fruits, Roots		Khan et al., 2015b
					Inflammation	Leaves, Fruits, Roots		Khan et al., 2015b
					Delivery wounds	Leaves, Fruits, Roots		Khan et al., 2015b

					Dysentery	Leaves, Fruits, Roots		Khan et al., 2015b
					Diabetic	Leaves, Fruits, Roots		Khan et al., 2015b
Berberis pseudumbellata R.Parke r	1	3	3.00	Berberidaceae	Delivery wounds	Roots, Leaves	Powder, Infusion	Ali et al., 2017
					Bone fracture	Roots, Leaves	Powder, Infusion	Ali et al., 2017
					Wound	Roots, Leaves	Powder, Infusion	Ali et al., 2017
Berberis vulgaris L.	1	1	1.00	Berberidaceae	Intestinal pain	Leaves	Raw	Ali et al., 2017
Bergenia ciliata (Haw.) Sternb.	7	9	1.28	Saxifragaceae	Hemostatic	Leaves	Raw	Abbasi et al., 2013
					Wound	Rhizome s	Powder	Abbasi et al., 2013
					Gastic problmes	Rhizome	Powder	Ahmed and Murtaza, 2015
					Wound	Leaves	Powder	Ali et al., 2017
					Antiseptic	Rhizome s	Powder	Shah et al., 2012
					Wound	Rhizome s	Powder	Shah et al., 2012
					Gastric problems	Leaves	Powder	Sher et al., 2004
					Gastrointestinal helminthiasis	Leaves	Raw	Sindhu et al., 2010
					Diarrhea	Rhizome s	Powder	Yousfzai et al., 2010
					Wound	Rhizome s	Powder	Yousfzai et al., 2010
Bergenia stracheyi (Hook.f. &	2	10	5.00	Saxifragaceae	Wound	Roots	Powder	Abbasi et al., 2013

Thomson) Engl.					Arthritis	Roots, Leaves		Khan et al., 2015b
					Backache	Roots, Leaves		Khan et al., 2015b
					Blood pressure	Roots, Leaves		Khan et al., 2015b
					Delivery wounds	Roots, Leaves		Khan et al., 2015b
					Diarrhea	Roots, Leaves		Khan et al., 2015b
					Dysentery	Roots, Leaves		Khan et al., 2015b
					Headache	Roots, Leaves		Khan et al., 2015b
					Skin diseases	Roots, Leaves		Khan et al., 2015b
					Vomiting	Roots, Leaves		Khan et al., 2015b
Beta vulgaris L.	3	3	1.00	Amaranthaceae	Digestive disorders	Whole plant	Powder	Aziz et al., 2018
					Galactagogue	Roots	Raw	Khan et al., 2015a
					Off feeding	Leaves		Ullah et al., 2017
Betula utilis D.Don	1	1	1.00	Betulaceae	Wound	Stems	Extract	Ali et al., 2017
Bistorta affinis (D.Don) Greene	2	4	2.00	Polygonaceae	Diarrhea	Leaves	Raw	Ali et al., 2017
					Arthritis	Leaves, Rhizome s		Khan et al., 2015b
					Rheumatism	Leaves, Rhizome s		Khan et al., 2015b
					Ulcers	Leaves, Rhizome		Khan et al., 2015b

						s		
Blepharis sindica Stocks ex T.Anderson	1	1	1.00	Acanthaceae	Galactagogue	Leaves	Raw	Khan, 2009
Boerhavia diffusa L.	2	3	1.50	Nyctaginaceae	Skin diseases	Whole plant	Extract	Khan and Hanif, 2006
					Stomach disorders	Whole plant	Extract	Khan and Hanif, 2006
					Urinary problems	Whole plant	Decoction	Khattak et al., 2015
Boerhavia erecta L.	1	1	1.00	Nyctaginaceae	Skin problems	Whole plant	Powder	Aziz et al., 2018
Boerhavia procumbens Banks ex Roxb.	1	2	2.00	Nyctaginaceae	Cough	Roots	Powder	Raza et al., 2014
					Respiratory diseases	Roots	Powder	Raza et al., 2014
Bombax ceiba L.	2	2	1.00	Malvaceae	Pain of leg, sciatica	Flowers	Powder	Khan and Hanif, 2006
					Laxative	Flowers	Raw	Raza et al., 2014
Bothriochloa bladhii (Retz.) S.T.Blake	1	1	1.00	Poaceae	Improves digestion	Aerial parts		Harun et al., 2017
Brachiaria ramosa (L.) Stapf	1	1	1.00	Poaceae	Antiseptic	Aerial parts		Harun et al., 2017
Brachiaria reptans (L.) C.A.Gardner & C.E.Hubb.	1	2	2.00	Poaceae	Anaemia	Whole plant	Juice	Harun et al., 2017
					Laxatives	Whole plant	Juice	Harun et al., 2017
Brassica rapa L.	23	86	3.74	Brassicaceae	Eye diseases	Seeds	Oil	Abbasi et al., 2013
					Infection	Seeds	Powder	Abbasi et al., 2013
					Skin infection	Seeds	Powder	Abbasi et al., 2013
					Stomach disorders	Seeds	Powder	Abbasi et al., 2013
					Mange	Seeds	Oil	Ali et al., 2017
					Allergy	Seeds	Powder	Aziz et al., 2018

					Appetizer	Seeds	Powder	Aziz et al., 2018
					Cough	Seeds	Powder	Aziz et al., 2018
					Skin infection	Seeds	Powder	Aziz et al., 2018
					Stomach disorders	Seeds	Powder	Aziz et al., 2018
					Tonic	Seeds	Powder	Aziz et al., 2018
					Galactagogue	Oil	Oil	Badar et al., 2017
					Haemorrhagic septicemia	Oil	Oil	Badar et al., 2017
					Bloat	Seeds	Oil	Deeba et al., 2009
					Diarrhea	Seeds	Powder	Deeba et al., 2009
					Ectoparasites	Seeds	Oil	Deeba et al., 2009
					Mastitis	Seeds	Oil	Deeba et al., 2009
					Genital prolapse	Seeds	Oil	Dilshad et al., 2008
					Genital prolapse	Flowers	Powder	Dilshad et al., 2008
					Mastitis	Seeds	Oil	Dilshad et al., 2010
					Helminthiasis	Seeds	Oil	Farooq et al., 2008
					Lice infestation	Seeds	Oil	Farooq et al., 2008
					Mange	Seeds	Oil	Farooq et al., 2008
					Mange	Seeds	Oil	Farooq et al., 2008
					Myiasis	Seeds	Oil	Farooq et al., 2008
					Myiasis	Seeds	Oil	Farooq et al., 2008
					Tick infestation	Seeds	Oil	Farooq et al., 2008
					Tick infestation	Seeds	Oil	Farooq et al., 2008
					Helminthes	Seeds	Oil	Hussain et al., 2008
					Gastric ailments	Leaves, Seeds	Oil	Islam et al., 2012
					Tonic	Leaves, Seeds	Oil	Islam et al., 2012
					Galactagogue	Seeds	Oil	Khan and Hanif,

								2006
					Blood in milk	Seeds	Oil	Khan et al., 2015a
					Diarrhea	Seeds	Oil	Khan et al., 2015a
					Foot, mouth diseases	Seeds	Oil	Khan et al., 2015a
					Off feeding	Seeds	Oil	Khan et al., 2015a
					Bloat	Seeds	Oil	Mirani et al., 2014
					Ectoparasites	Seeds	Oil	Mirani et al., 2014
					Endoparasites	Seeds	Oil	Mirani et al., 2014
					Foot diseases	Seeds	Oil	Mirani et al., 2014
					Foot diseases	Seeds	Oil	Mirani et al., 2014
					Infertility	Seeds	Oil	Mirani et al., 2014
					Myiasis	Seeds	Oil	Mirani et al., 2014
					Tympany	Seeds	Oil	Mirani et al., 2014
					Broken horns	Seeds	Oil	Mirani et al., 2016
					Diarrhea	Seeds	Oil	Mirani et al., 2016
					Dysentery	Seeds	Oil	Mirani et al., 2016
					Endoparasites	Seeds	Oil	Mirani et al., 2016
					Endoparasites	Seeds	Oil	Mirani et al., 2016
					Foot Diseases	Seeds	Oil	Mirani et al., 2016
					Galactagogue	Seeds	Oil	Mirani et al., 2016
					Infertility	Seeds	Oil	Mirani et al., 2016
					Mastitis	Seeds	Oil	Mirani et al., 2016
					Mouth diseases	Seeds	Oil	Mirani et al., 2016
					Myiasis	Seeds	Oil	Mirani et al., 2016
					Uterine prolapse	Seeds	Oil	Mirani et al., 2016
					Digestive disorders	Seeds		Muhammad et al., 2005
					Systemic disorders	Seeds		Muhammad et al., 2005

					Mastitis	Seeds	Oil	Mussarat et al., 2014
					Placenta expulsion	Seeds	Decoction, Powder	Mussarat et al., 2014
					Mange	Seeds	Oil	Raza et al., 2014
					Tick infestation	Seeds	Oil	Raza et al., 2014
					Tonic	Seeds	Oil	Shah et al., 2012
					Tonic	Seeds	Oil	Shah et al., 2012
					Gastrointestinal helminthiaisis	Seeds	Oil	Sindhu et al., 2010
					Gastrointestinal helminthiasis	Seeds	Oil	Sindhu et al., 2010
					Mange	Oil	Powder	Sindhu et al., 2010
					Pediculosis	Oil	Powder	Sindhu et al., 2010
					Pediculosis	Oil	Powder	Sindhu et al., 2010
					Pediculosis	Oil	Powder	Sindhu et al., 2010
					Fly infestation	Seeds	Oil	Sindhu et al., 2012
					Helminthiasis	Leaves	Powder	Sindhu et al., 2012
					Lice infestation	Seeds	Oil	Sindhu et al., 2012
					Mange	Seeds	Oil	Sindhu et al., 2012
					Tick infestation	Seeds	Oil	Sindhu et al., 2012
					Lice infestation	Whole plant	Paste	Tariq et al., 2014
					Flatulence	Seeds	Oil	Ul Hassan et al., 2014
					Burn	Seeds	Oil	Ullah et al., 2017
					Dog bite	Seeds	Oil	Ullah et al., 2017
					Dysentery	Seeds	Oil	Ullah et al., 2017
					Laxative	Seeds	Oil	Ullah et al., 2017
					Skin diseases	Seeds	Oil	Ullah et al., 2017
					Sore	Seeds	Oil	Ullah et al., 2017
					Stomachic	Seeds	Oil	Ullah et al., 2017

					Tonic	Seeds	Oil	Ullah et al., 2017
Bromus japonicus Thunb.	2	2	1.00	Poaceae	Constipation	Aerial parts		Harun et al., 2017
					Constipation	Shoot	Powder	Yousfzai et al., 2010
Bryophyllum pinnatum (Lam.) Oken	2	4	2.00	Crassulaceae	Wound	Leaves	Raw	Abbasi et al., 2013
					Allergy	Leaves	Decoction	Khan and Hanif, 2006
					Boils	Leaves	Decoction	Khan and Hanif, 2006
					Wound	Leaves	Decoction	Khan and Hanif, 2006
Buddleja asiatica Lour.	1	1	1.00	Scrophulariaceae	Mites	Leaves	Powder	Shah et al., 2012
Bunium persicum (Boiss.) B.Fedtsch.	2	2	1.00	Apiaceae	Mastitis	Fruits	Decoction	Khan et al., 2015a
					Febrifuge	Fruits		Yousfzai et al., 2010
Butea monosperma (Lam.) Taub.	5	7	1.40	Fabaceae	Worm infestation	Gum	Powder	Badar et al., 2017
					Genital prolapse	Gum	Powder	Dilshad et al., 2008
					Galactagogue	Leaves		Khan and Hanif, 2006
					Anthelmintic	Seeds	Powder	Khan, 2009
					Skin infection	Flowers	Paste	Khan, 2009
					Ulcers	Seeds	Powder	Shah et al., 2012
					Worms from intestine	Seeds	Powder	Shah et al., 2012
Buxus wallichiana Baill.	1	1	1.00	Buxaceae	Mange	Leaves	Powder, Decoction	Ahmad et al., 2015
Calligonum polygonoides L.	3	4	1.33	Polygonaceae	Fly infestation	Aerial parts	Smoke	Farooq et al., 2008
					Analgesic	Whole plant	Ash	Khattak et al., 2015

					Astringent	Whole plant	Ash	Khattak et al., 2015
					Skin problems	Aerial parts	Decoction	Raza et al., 2014
Calluna vulgaris (L.) Hull	1	1	1.00	Ericaceae	Genital prolapse	Seeds	Raw	Dilshad et al., 2008
Calotropis procera (Aiton) Dryand.	13	33	2.54	Apocynaceae	Analgesic	Stem	Powder	Abbasi et al., 2013
					Colic	Stem	Raw	Abbasi et al., 2013
					Eye diseases	Leaves	Raw	Abbasi et al., 2013
					Indigestion	Stem	Raw	Abbasi et al., 2013
					Inflammation	Stem	Powder	Abbasi et al., 2013
					Mouth diseases	Leaves	Raw	Abbasi et al., 2013
					Pain of joint	Leaves	Decoction	Aziz et al., 2018
					Worm infestation	Flowers	Powder	Badar et al., 2017
					Wound	Flowers	Powder	Badar et al., 2017
					Toxaemia	Flowers	Powder	Badar et al., 2017
					Delayed puberty	Leaves		Dilshad et al., 2008
					Silent estrous	Leaves		Dilshad et al., 2008
					Appetizer	Leaves, Flowers	Powder	Khan and Hanif, 2006
					Digestion	Leaves, Flowers	Powder	Khan and Hanif, 2006
					Snake bite	Latex		Khan and Hanif, 2006
					Snake bite	Latex	Latex	Khan, 2009
					Anorexia	Leaves, Flowers	Powder	Khan, 2009
					Dog bite	Roots	Powder	Khan, 2009
					Flatulence	Leaves, Flowers	Powder	Khan, 2009

					Indigestion	Leaves, Flowers	Powder	Khan, 2009
					Intestinal worm	Leaves, Flowers	Powder	Khan, 2009
					Worm	Flowers, Stems, Leaves	Powder, Decoction	Khattak et al., 2015
					Mastitis	Latex		Mirani et al., 2014
					Myiasis	Latex		Mirani et al., 2014
					Placenta expulsion	Latex		Mirani et al., 2014
					Mastitis	Latex		Mirani et al., 2016
					Placenta expulsion	Latex		Mirani et al., 2016
					Digestive diseases	Leaves, Flowers	Powder	Raza et al., 2014
					Anti-inflammatory	Milky Juice		Raza et al., 2014
					Helminthiasis	Flowers	Powder	Sindhu et al., 2012
					Intestinal worm	Fruits, Leaves	Paste, Concoction	Tariq et al., 2014
					Skin infections	Fruits, Leaves	Paste, Concoction	Tariq et al., 2014
					Dogbite	Latex	Latex	Yousfzai et al., 2010
					Snake bite	Latex	Latex	Yousfzai et al., 2010
Camellia sinensis (L.) Kuntze	8	19	2.38	Theaceae	Fever	Leaves	Decoction	Abbasi et al., 2013
					Diarrhea	Leaves	Powder	Deeba et al., 2009
					Retention of placenta	Leaves	Decoction	Dilshad et al., 2008
					Abscess	Leaves	Decoction	Khan et al., 2015a
					Black quarter	Leaves	Decoction	Khan et al., 2015a
					Colic	Leaves	Decoction	Khan et al., 2015a

					Cough	Leaves	Decoction	Khan et al., 2015a
					Subnormal	Leaves	Decoction	Khan et al., 2015a
					Temprature	Leaves	Decoction	Khan et al., 2015a
					Fever	Leaves	Decoction	Khan et al., 2015a
					Tympany	Leaves	Decoction	Khan et al., 2015a
					Bloat	Leaves	Decoction	Mirani et al., 2014
					Tympany	Leaves	Decoction	Mirani et al., 2014
					Bloat	Leaves	Decoction	Mirani et al., 2016
					Tympany	Leaves	Decoction	Mirani et al., 2016
					Systemic disorders	Seeds		Muhammad et al., 2005
					Cough	Leaves		Ullah et al., 2017
					Fever	Leaves		Ullah et al., 2017
					Indigestion	Leaves		Ullah et al., 2017
Cannabis sativa L.	11	26	2.36	Cannabaceae	Abdominal Pain	Leaves	Powder	Abbasi et al., 2013
					Appetizer	Leaves	Powder	Abbasi et al., 2013
					Indigestion	Leaves	Powder	Abbasi et al., 2013
					Indigestion	Leaves, flowers, Seeds	Powder	Abbasi et al., 2013
					Leeches	Leaves	Raw	Abbasi et al., 2013
					Lice infestation	Leaves	Raw	Abbasi et al., 2013
					Antiparasite	Whole plant	Infusion	Aziz et al., 2018
					Gastrointestinal problems	Whole plant	Infusion	Aziz et al., 2018
					Stimulant	Whole plant	Paste, Powder, Infusion	Aziz et al., 2018
					Panting	Seeds	Powder	Badar et al., 2017
					Prolapse	Seeds	Powder	Badar et al., 2017

					Genital prolapse	Leaves		Dilshad et al., 2008
					Ectoparasites	Flowers	Infusion	Khan and Hanif, 2006
					Flatulence	Flowers	Extract	Khan and Hanif, 2006
					Measles	Flowers	Infusion	Khan and Hanif, 2006
					Off feeding	Seeds	Powder	Khan et al., 2015a
					Appetizer	Leaves	Powder	Shah et al., 2012
					Pediculosis	Leaves	Powder	Sindhu et al., 2010
					Tick infestation	Leaves	Powder	Sindhu et al., 2010
					Uterine prolapse	Leaves	Powder	Sindhu et al., 2010
					Appetizer	Leaves	Powder	Tariq et al., 2014
					Ectoparasites	Leaves	Powder	Tariq et al., 2014
					Mastitis	Leaves, Seeds	Decoction	Tariq et al., 2016
					Off feeding	Seeds		Ullah et al., 2017
					Regulate body temp	Seeds		Ullah et al., 2017
Capparis decidua (Forssk.) Edgew.	11	21	1.91	Capparaceae	Bone fracture	Wood	Powder	Ahmad et al., 2015
					Antiparasite	Whole plant	Powder	Aziz et al., 2018
					Gastric ailments	Whole plant	Powder	Aziz et al., 2018
					Mastitis	Fruits	Powder	Dilshad et al., 2010
					Lice infestation	Shoot	Ash	Farooq et al., 2008
					Myiasis	Shoot	Raw	Farooq et al., 2008
					Myiasis	Shoot, Fruits	Raw	Farooq et al., 2008

					Helminthes	Aerial parts	Powder	Hussain et al., 2008
					Anorexia	Barks	Powder	Khan, 2009
					Diarrhea	Fruits		Khan, 2009
					Indigestion	Barks	Powder	Khan, 2009
					Rheumatism	Barks	Powder	Khan, 2009
					Astringent	Stems	Ash	Khattak et al., 2015
					Bone fracture	Stems		Khattak et al., 2015
					Bone fracture	Roots	Powder	Mirani et al., 2016
					Halitosis	Fruits		Muhammad et al., 2005
					Indigestion	Fruits		Muhammad et al., 2005
					Helminthiasis	Aerial parts	Raw	Raza et al., 2014
					Wound	Leaves, Twigs		Raziq et al., 2010
					Helminthiasis	Roots	Decoction	Sindhu et al., 2012
					Tick infestation	Roots	Decoction	Sindhu et al., 2012
Capparis spinosa L.	3	4	1.33	Capparaceae	Pain of joint	Seeds	Oil	Ali et al., 2017
					Genital prolapse	Seeds	Powder	Dilshad et al., 2008
					Cough	Aerial parts		Raza et al., 2014
					Analgesic	Aerial parts	Decoction	Raza et al., 2014
					Respiratory diseases	Aerial parts		Raza et al., 2014
Capsicum annuum L.	12	34	2.83	Solanaceae	Allergy	Fruits	Raw	Badar et al., 2017
					Anestrus	Fruits	Raw	Badar et al., 2017
					Indigestion	Fruits	Raw	Badar et al., 2017
					Toxaemia	Fruits	Raw	Badar et al., 2017

					Worm infestation	Leaves	Raw	Badar et al., 2017
					Anorexia	Fruits	Powder	Deeba et al., 2009
					Mastitis	Fruits	Decoction	Deeba et al., 2009
					Mastitis	Fruits	Powder	Deeba et al., 2009
					Mastitis	Fruits	Powder	Dilshad et al., 2010
					Helminthiasis	Fruits	Raw	Farooq et al., 2008
					Helminthiasis	Fruits	Raw	Farooq et al., 2008
					Helminthiasis	Fruits	Raw	Farooq et al., 2008
					Helminthes	Fruits	Powder	Hussain et al., 2008
					Bad evil	Fruits	Raw	Khan and Hanif, 2006
					Stomach disorders	Fruits	Raw	Khan and Hanif, 2006
					Wound	Roots	Powder	Khan and Hanif, 2006
					Colic	Fruits	Raw	Khan et al., 2015a
					Foot, mouth	Fruits	Powder	Khan et al., 2015a
					Mastitis	Fruits	Powder	Khan et al., 2015a
					Off feeding	Fruits	Powder	Khan et al., 2015a
					Endoparasites	Fruits	Powder	Mirani et al., 2014
					Mastitis	Fruits	Powder	Mirani et al., 2014
					Diarrhea	Fruits	Powder	Mirani et al., 2016
					Dysentery	Fruits	Powder	Mirani et al., 2016
					Endoparasites	Fruits	Powder	Mirani et al., 2016
					Mastitis	Fruits	Powder	Mirani et al., 2016
					Respiratory	Fruits	Powder	Mirani et al., 2016
					Halitosis	Fruits		Muhammad et al., 2005
					Indigestion	Fruits		Muhammad et al., 2005
					Systemic	Fruits		Muhammad et al.,

					disorders			2005
					Mastitis	Whole plant	Powder	Mussarat et al., 2014
					Helminthiasis	Flowers	Powder	Sindhu et al., 2012
					Mange	Fruits	Powder	Sindhu et al., 2012
					Off feeding	Fruits		Ullah et al., 2017
Caralluma tuberculata N.E.Br.	1	1	1.00	Apocynaceae	Retention of placenta	Whole plant	Decoction	Tariq et al., 2016
Carissa caranta L.	1	2	2.00	Apocynaceae	Foot diseases	Roots	Powder	Khan et al., 2012
					Mouth diseases	Roots	Powder	Khan et al., 2012
Carissa spinarum L.	1	3	3.00	Apocynaceae	Respiratory diseases	Leaves, Seeds	Powder	Abbasi et al., 2013
					Sores	Roots	Powder	Abbasi et al., 2013
					Wound	Roots	Powder	Abbasi et al., 2013
Carthamus oxyacantha M.Bieb.	1	2	2.00	Asteraceae	Wound	Seeds	Oil	Tariq et al., 2016
					Wound	Seeds	Powder	Tariq et al., 2016
Carum carvi L.	3	7	2.33	Apiaceae	Digestive disorders	Fruits	Decoction	Aziz et al., 2018
					Flatulence	Seeds	Raw	Khan et al., 2015a
					Foot diseases	Seeds	Raw	Khan et al., 2015a
					Tympany	Seeds	Raw	Khan et al., 2015a
					Fever	Seeds		Ullah et al., 2017
					Gastric ailments	Seeds		Ullah et al., 2017
					Skin diseases	Seeds		Ullah et al., 2017
Cassia fistula L.	4	7	1.75	Fabaceae	Fever	Fruits	Raw	Aziz et al., 2018
					Gastric ailments	Fruits	Raw	Aziz et al., 2018
					Colic	Fruits	Decoction	Khan et al., 2015a
					Diarrhea	Fruits	Decoction	Mussarat et al., 2014
					Pain in joints	Fruits	Decoction	Mussarat et al., 2014
					Pain in muscles	Fruits	Decoction	Mussarat et al., 2014

					Analgesic	Fruits		Ullah et al., 2017
Catharanthus roseus (L.) G.Don	1	1	1.00	Apocynaceae	Maggots from wound	Whole plant	Powder	Shah et al., 2012
Catunaregam spinosa (Thunb.) Tirveng.	1	2	2.00	Rubiaceae	Delayed puberty	Fruits	Raw	Dilshad et al., 2008
					Silent estrous	Fruits	Powder	Dilshad et al., 2008
Cedrus deodara (Roxb. ex D.Don) G.Don	4	14	3.50	Pinaceae	Gastric ailments	Wood	Oil	Aziz et al., 2018
					Infertility	Wood	Oil	Aziz et al., 2018
					Refrigerant	Wood	Oil	Aziz et al., 2018
					Off feeding	Wood	Oil	Khan et al., 2015a
					Worm infestation	Wood	Oil	Khan et al., 2015a
					Antifungal	Wood	Oil	Sindhu et al., 2010
					Gastrointestinal helminthiasis	Wood	Oil	Sindhu et al., 2010
					Mange	Wood	Oil	Sindhu et al., 2010
					Mosquito repellent	Wood	Oil	Sindhu et al., 2010
					Pediculosis	Wood	Oil	Sindhu et al., 2010
					Pediculosis	Wood	Oil	Sindhu et al., 2010
					Tick infestation	Wood	Oil	Sindhu et al., 2010
					Flatulence	Wood	Oil	Yousfzai et al., 2010
					Stomachic problems	Wood	Oil	Yousfzai et al., 2010
Celtis australis L.	1	1	1.00	Cannabaceae	Skin problems	Barks	Powder	Aziz et al., 2018
Centella asiatica (L.) Urb.	1	2	2.00	Apiaceae	Analgesic	Stems, Roots	Smoke	Khan and Hanif, 2006
					Skin diseases	Roots	Decoction	Khan and Hanif, 2006
Chamaecyparis obtusa (Siebold & Zucc.) Endl.	1	1	1.00	Cupressaceae	Sex tonic	Fruits	Raw	Aziz et al., 2018

Chenopodium album L.	6	8	1.33	Amaranthaceae	Wound	Leaves	Decoction	Abbasi et al., 2013
					Flatulence	Whole plant	Decoction	Aziz et al., 2018
					Wound	Leaves	Decoction	Aziz et al., 2018
					Measles	Whole plant	Decoction	Khan and Hanif, 2006
					Skin diseases	Whole plant	Decoction	Khan and Hanif, 2006
					Skin diseases	Whole plant	Decoction	Khan et al., 2012
					Wound	Whole plant	Paste	Tariq et al., 2014
					Flatulence	Whole plant	Powder	Ul Hassan et al., 2014
Chenopodium murale L.	1	1	1.00	Amaranthaceae	Anthelmentic	Roots, Shoot	Powder	Yousfzai et al., 2010
Chrysopogon zizanioides (L.) Roberty	1	2	2.00	Poaceae	Antiseptic	Leaves		Harun et al., 2017
					Inflammation	Leaves		Harun et al., 2017
Cicer arietinum L.	3	3	1.00	Fabaceae	Piles	Seeds	Powder	Abbasi et al., 2013
					Weakness	Seeds	Raw	Khan et al., 2015a
					Weakness	Seeds		Ullah et al., 2017
Cichorium intybus L.	3	4	1.33	Asteraceae	Inflammation	Whole plant		Islam et al., 2012
					Stomach problems	Whole plant		Islam et al., 2012
					Fever	Roots	Powder	Sher et al., 2004
					Fever	Leaves, Roots	Decoction	Ullah et al., 2017
Cinnamomum camphora (L.) J.Presl	2	2	1.00	Lauraceae	Bloat	Gum	Powder	Deeba et al., 2009
					Genital prolapse	Gum	Powder	Dilshad et al., 2008

Cinnamomum verum J.Presl	3	3	1.00	Lauraceae	Diarrhea	Seeds	Powder	Deeba et al., 2009
					Genital prolapse	Seeds	Powder	Dilshad et al., 2008
					Weakness	Fruits	Powder	Khan et al., 2015a
Cissampelos pareira L.	1	1	1.00	Menispermacea e	Tonic	Leaves	Powder	Shah et al., 2012
Cistanche tubulosa (Schenk) Wight	1	1	1.00	Orobanchaceae	Cough	Whole plant	Decoction	Khattak et al., 2015
Citrullus colocynthis (L.) Schrad.	16	43	2.69	Cucurbitaceae	Constipation	Fruits	Powder	Abbasi et al., 2013
					Flatulance	Fruits	Juice	Abbasi et al., 2013
					Indigestion	Fruits	Juice	Abbasi et al., 2013
					Worms from intestine	Fruits	Juice	Abbasi et al., 2013
					Tympany	Leaves	Powder	Badar et al., 2017
					Mastitis	Fruits	Raw	Dilshad et al., 2010
					Helminthiasis	Fruits	Powder	Farooq et al., 2008
					Lice infestation	Fruits	Decoction	Farooq et al., 2008
					Helminthes	Fruits	Powder	Hussain et al., 2008
					Wound	Whole plant	Ash	Khan and Hanif, 2006
					Anthelmentic	Whole plant	Powder	Khan and Hanif, 2006
					Colic	Fruits	Juice	Khan et al., 2015a
					Anthelmentic	Whole plant	Paste	Khan, 2009
					Jaundice	Roots		Khan, 2009
					Purgative	Whole plant		Khan, 2009
					Rheumatism	Roots		Khan, 2009
					Rheumatism	Fruits	Powder	Khan, 2009

					Wound	Whole plant	Ash	Khan, 2009
					Carminative	Fruits, Seeds		Khattak et al., 2015
					Gastric ailments	Fruits, Seeds		Khattak et al., 2015
					Bloat	Fruits	Powder	Mirani et al., 2014
					Ectoparasites	Fruits	Decoction	Mirani et al., 2014
					Endoparasites	Fruits	Decoction	Mirani et al., 2014
					Tympany	Fruits	Powder	Mirani et al., 2014
					Bloat	Fruits	Powder	Mirani et al., 2016
					Ectoparasites	Fruits	Decoction	Mirani et al., 2016
					Endoparasites	Fruits	Decoction	Mirani et al., 2016
					Galactagogue	Fruits	Powder	Mirani et al., 2016
					Tympany	Fruits	Powder	Mirani et al., 2016
					Halitosis	Fruits		Muhammad et al., 2005
					Indigestion			Muhammad et al., 2005
					Mastitis	Fruits	Powder	Mussarat et al., 2014
					Skin diseases	Roots	Poultice	Mussarat et al., 2014
					Digestive disorders	Fruits	Powder	Raza et al., 2014
					Helminthiasis	Fruits	Powder	Raza et al., 2014
					Indigestion	Fruits	Powder	Raza et al., 2014
					Fly infestation	Fruits	Decoction	Sindhu et al., 2012
					Helminthiasis	Fruits	Powder	Sindhu et al., 2012
					Lice infestation	Fruits	Decoction	Sindhu et al., 2012
					Mange	Fruits	Paste	Sindhu et al., 2012

					Mange	Fruits	Paste	Sindhu et al., 2012
					Tick infestation	Fruits	Raw	Sindhu et al., 2012
					Skin infection	Roots	Juice	Tariq et al., 2014
					Abdominal pain	Fruits	Powder	Ul Hassan et al., 2014
Citrullus vulgaris Schrad.	13	3	0.23	Cucurbitaceae	Appetizer	Fruits	Raw	Abbasi et al., 2013
					Galactagogue	Fruits	Raw	Abbasi et al., 2013
					Hepatitis	Fruits	Juice	Khan et al., 2015a
Citrus limon (L.) Osbeck	11	17	1.55	Rutaceae	Mastitis	Fruits	Juice	Abbasi et al., 2013
					Mastitis	Fruits	Juice	Aziz et al., 2018
					Galactagogue	Fruits	Powder	Badar et al., 2017
					Jaundice	Fruits	Powder	Badar et al., 2017
					Mastitis	Fruits	Powder	Badar et al., 2017
					Diarrhea	Fruits	Juice	Deeba et al., 2009
					Mastitis	Fruits	Infusion	Dilshad et al., 2010
					Prolapse of uterus	Fruits	Juice	Khan and Hanif, 2006
					Mastitis	Fruits	Juice	Khan et al., 2015a
					Mastitis	Fruits	Juice	Mirani et al., 2014
					Ectoparasites	Fruits	Juice	Mirani et al., 2016
					Mastitis	Fruits	Juice	Mirani et al., 2016
					Mastitis	Fruits	Juice	Mussarat et al., 2014
					Stomach problems	Fruits	Juice	Mussarat et al., 2014
					Nerve tonic	Fruits	Juice	Raza et al., 2014
					Refrigerant	Fruits	Juice	Raza et al., 2014
					Inflammation	Fruits		Ullah et al., 2017
Citrus medica L.	2	2	1.00	Rutaceae	Bloat	Fruits	Raw	Khan et al., 2015a
					Bloat	Fruits		Ullah et al., 2017
Citrus reticulata Blanco	2	3	1.50	Rutaceae	Lochial	Leaves	Decoction	Dilshad et al., 2008

					discharge			
					Diarrhea	Leaves	Decoction	Mussarat et al., 2014
					Mastitis	Leaves	Poultice	Mussarat et al., 2014
Clematis grata Wall.	2	2	1.00	Ranunculaceae	Worms in wounds	Leaves	Raw	Abbasi et al., 2013
					Rinderpest effects	Leaves, Flowers	Powder	Khan and Hanif, 2006
Cleome brachycarpa (Forssk.) Vahl ex DC.	2	2	1.00	Cleomaceae	Maggots in nostrils	Leaves	Powder	Khan, 2009
					Maggots in nostrils	Leaves	Powder	Raza et al., 2014
Clerodendrum phlomidis L.f.	1	2	2.00	Lamiaceae	Edema	Roots	Decoction	Khan, 2009
					Indigestion	Roots	Decoction	Khan, 2009
Cocculus hirsutus (L.) W.Theob.	1	3	3.00	Menispermaceae	Laxative	Aerial parts	Decoction	Raza et al., 2014
					Nerve tonic	Aerial parts	Decoction	Raza et al., 2014
					Refrigerant	Leaves	Decoction	Raza et al., 2014
Cochlospermum religiosum (L.) Alston	1	1	1.00	Bixaceae	Genital prolapse	Leaves	Decoction	Dilshad et al., 2008
Cocos nucifera L.	3	3	1.00	Arecaceae	Helminthes	Fruits	Powder	Hussain et al., 2008
					Infertility	Fruits	Raw	Mirani et al., 2014
					Infertility	Fruits	Raw	Mirani et al., 2016
Colebrookea oppositifolia Sm.	3	3	1.00	Commelinaceae	Eye diseases	Leaves	Juice	Shah et al., 2012
					Eye diseases	Leaves	Powder	Shah et al., 2012
					Maggots from wound	Leaves	Powder	Shah et al., 2012
Commiphora wightii (Arn.) Bhandari	2	2	1.00	Burseraceae	Indigestion	Leaves	Raw	Badar et al., 2017
					Respiratory	Whole plant		Khan, 2009

Convolvulus arvensis L.	9	10	1.11	Convolvulaceae	Constipation	Whole plant	Raw	Abbasi et al., 2013
					Galactagogue	Whole plant	Raw	Aziz et al., 2018
					Helminthes	Aerial parts	Powder	Hussain et al., 2008
					Galactagogue	Whole plant	Powder	Khan et al., 2015a
					Skeletomuscular ailments	Roots	Powder	Khattak et al., 2015
					Wound	Roots	Powder	Khattak et al., 2015
					Helminthiasis	Aerial parts	Raw	Raza et al., 2014
					Helminthiasis	Plant	Powder	Sindhu et al., 2012
					Constipation	Whole plant	Powder	Tariq et al., 2014
					Galactagogue	Whole plant		Ullah et al., 2017
Corchorus depressus (L.) Stocks	1	1	1.00	Malvaceae	Diuretic	Aerial parts	Decoction	Raza et al., 2014
Corchorus olitorius L.	1	1	1.00	Malvaceae	Retention of placenta	Seeds	Decoction	Dilshad et al., 2008
Cordia dichotoma G.Forst.	4	4	1.00	Boraginaceae	Common cold	Fruits	Raw	Abbasi et al., 2013
					Respiratory	Fruits	Raw	Abbasi et al., 2013
					Stomach problems	Seeds	Decoction	Abbasi et al., 2013
					Analgesic	Barks		Ullah et al., 2017
Cordia macleodii Hook.f. & Thomson	2	2	1.00	Boraginaceae	Astringent	Stems		Ullah et al., 2017
					Relief from insanity	Stems		Ullah et al., 2017
Cordia myxa L.	1	2	2.00	Boraginaceae	Galactogague	Leaves		Khattak et al., 2015

					Irritation	Fruits		Khattak et al., 2015
Coriandrum sativum L.	6	8	1.33	Apiaceae	Pain of stomach	Seeds	Powder	Ali et al., 2017
					Genital prolapse	Seeds	Raw	Dilshad et al., 2008
					Helminthes	Seeds	Powder	Hussain et al., 2008
					Foot diseases	Fruits	Decoction	Khan et al., 2015a
					Diuretic	Leaves, Roots	Decoction	Tariq et al., 2014
					Foot diseases	Leaves		Ullah et al., 2017
					Mouth diseases	Leaves		Ullah et al., 2017
					Regulate body temp	Leaves		Ullah et al., 2017
Crocus sativus L.	1	1	1.00	Iridaceae	Wound	Flowers	Paste	Ali et al., 2017
Crotalaria burhia Benth.	1	2	2.00	Fabaceae	Itching	Aerial parts	Paste	Raza et al., 2014
					Skin problems	Aerial parts	Paste	Raza et al., 2014
Crotalaria juncea L.	1	1	1.00	Fabaceae	Galactagogue	Seeds	Powder	Shah et al., 2012
Cucumis melo L.	2	2	1.00	Cucurbitaceae	Indigestion	Leaves, Fruits	Raw	Abbasi et al., 2013
					Helminthes	Whole plant	Decoction	Hussain et al., 2008
Cucurbita maxima Duchesne	2	3	1.50	Cucurbitaceae	Prolapse	Seeds	Powder	Badar et al., 2017
					Worm infestation	Seeds	Powder	Badar et al., 2017
					Genital prolapse	Fruits	Raw	Dilshad et al., 2008
Cucurbita pepo L.	1	1	1.00	Cucurbitaceae	Vaginal prolapse	Fruits	Raw	Khan et al., 2015a
Cuminum cyminum L.	8	15	1.88	Apiaceae	Gastric ailments	Fruits	Decoction	Aziz et al., 2018
					Galactagogue	Seeds	Powder	Badar et al., 2017
					Heat stroke	Seeds	Powder	Badar et al., 2017

					Jaundice	Seeds	Powder	Badar et al., 2017
					Panting	Seeds	Powder	Badar et al., 2017
					Anestrus	Seeds	Raw	Dilshad et al., 2008
					Mastitis	Seeds	Raw	Dilshad et al., 2010
					Prolapse of uterus	Seeds	Raw	Khan and Hanif, 2006
					Galactagogue	Seeds	Decoction	Mirani et al., 2014
					Bloat	Seeds	Powder	Mirani et al., 2016
					Diarrhea	Seeds	Decoction	Mirani et al., 2016
					Dysentery	Seeds	Decoction	Mirani et al., 2016
					Galactagogue	Seeds	Decoction	Mirani et al., 2016
					Tympany	Seeds	Powder	Mirani et al., 2016
					Mastitis	Stems	Powder	Mussarat et al., 2014
Curcuma longa L.	13	21	1.62	Zingiberaceae	Wound	Rhizomes	Powder	Abbasi et al., 2013
					Jaundice	Rhizomes	Powder	Ahmad et al., 2015
					Skeletomuscular ailments	Rhizomes	Powder	Ahmad et al., 2015
					Internal body infection	Rhizomes	Powder	Aziz et al., 2018
					Jaundice	Rhizomes	Powder	Aziz et al., 2018
					Skeletomuscular ailments	Rhizomes	Powder	Aziz et al., 2018
					Haemorrhagic septicemia	Rhizomes	Decoction	Badar et al., 2017
					Bloat	Rhizomes	Powder	Deeba et al., 2009
					Mastitis	Roots	Powder	Dilshad et al., 2010
					Wound	Rhizomes	Powder	Khan et al., 2015a

					Vaginal prolapse	Rhizomes	Powder	Khan et al., 2015a
					Broken horns	Rhizomes	Powder	Mirani et al., 2016
					Mastitis	Rhizomes	Powder	Mussarat et al., 2014
					Skin infections	Seeds	Powder	Raza et al., 2014
					Wound	Seeds	Powder	Raza et al., 2014
					Bone fracture	Rhizomes	Powder	Shah et al., 2012
					Wound	Rhizomes	Powder	Shah et al., 2012
					Wound	Leaves	Decoction	Tariq et al., 2014
					Hair losing	Rhizomes		Ullah et al., 2017
					Wound	Rhizomes		Ullah et al., 2017
Curcuma picta Roxb. ex Škorničk.	1	1	1.00	Zingiberaceae	Mange	Bulbs	Powder	Sindhu et al., 2012
Cuscuta reflexa Roxb.	7	12	1.71	Convolvulaceae	Galactagogue	Whole plant	Powder	Abbasi et al., 2013
					Indigestion	Whole plant	Raw	Abbasi et al., 2013
					Constipation	Stems, Flowers	Raw	Ali et al., 2017
					Diarrhea	Stems, Flowers	Raw	Ali et al., 2017
					Placenta expulsion	Stems, Flowers	Raw	Ali et al., 2017
					Muscular problems	Whole plant	Paste	Aziz et al., 2018
					Helminthes	Whole plant	Decoction	Hussain et al., 2008

					Astringent	Whole plant	Powder	Khattak et al., 2015
					Diaphoretic	Whole plant	Powder	Khattak et al., 2015
					Fertility agent	Whole plant	Powder	Khattak et al., 2015
					Helminthiasis	Aerial parts	Raw	Raza et al., 2014
					Ectoparasites	Stems, Seeds	Decoction	Tariq et al., 2016
Cyamopsis tetragonoloba (L.) Taub.	2	2	1.00	Fabaceae	Galactagogue	Fruits	Raw	Mirani et al., 2014
					Galactagogue	Fruits	Raw	Mirani et al., 2016
Cymbopogon citratus (DC.) Stapf	1	1	1.00	Poaceae	Hepatitis	Leaves	Decoction	Khan et al., 2015a
Cymbopogon jwarancusa (Jones) Schult.	3	7	2.33	Poaceae	Diuretic	Whole plant		Harun et al., 2017
					Improve fertility	Whole plant		Harun et al., 2017
					Genital problems	Whole plant		Khattak et al., 2015
					Sex tonic	Whole plant		Khattak et al., 2015
					Urinary problems	Whole plant		Khattak et al., 2015
					Cough	Leaves, Flowers	Decoction	Raza et al., 2014
					Respiratory	Leaves, Flowers	Decoction	Raza et al., 2014
Cynanchum viminale (L.) L.	1	3	3.00	Apocynaceae	Dog bite	Whole plant	Infusion	Khan, 2009
					Snake bite	Whole plant	Powder	Khan, 2009

					Wound	Whole plant	Powder	Khan, 2009
Cynodon dactylon (L.) Pers.	5	12	2.40	Poaceae	Wound	Whole plant	Raw	Abbasi et al., 2013
					Dysentery	Whole plant	Paste	Harun et al., 2017
					Inflammation	Whole plant	Paste	Harun et al., 2017
					Wound	Whole plant	Paste	Harun et al., 2017
					Analgesic	Stems, Leaves		Islam et al., 2012
					Antiseptic	Stems, Leaves		Islam et al., 2012
					Wounds	Stems, Leaves		Islam et al., 2012
					Tonic	Stems, Leaves		Islam et al., 2012
					Dysentery	Whole plant	Paste	Khattak et al., 2015
					Wound	Whole plant	Paste	Khattak et al., 2015
					Wound	Whole plant	Paste	Khattak et al., 2015
					Analgesic	Whole plant	Concoction	Tariq et al., 2014
					Wound	Whole plant	Concoction	Tariq et al., 2014
Cynoglossum lanceolatum Forssk.	1	1	1.00	Boraginaceae	Common cold	Roots	Powder	Tariq et al., 2014
Cyperus niveus Retz.	1	3	3.00	Cyperaceae	Common cold	Whole plant	Powder	Tariq et al., 2014
					Pain of joints	Whole	Powder	Tariq et al., 2014

						plant		
					Stomach worms	Whole plant	Powder	Tariq et al., 2014
Cyperus rotundus L.	2	2	1.00	Cyperaceae	Helminthiasis	Roots	Powder	Farooq et al., 2008
					Diuretic	Roots	Decoction	Raza et al., 2014
Dactylis glomerata L.	1	1	1.00	Poaceae	Diuretic	Leaves		Harun et al., 2017
Dalbergia sissoo DC.	9	11	1.22	Fabaceae	Constipation	Leaves	Decoction	Abbasi et al., 2013
					Prolapse	Leaves	Decoction	Badar et al., 2017
					Genital prolapse	Barks	Powder	Dilshad et al., 2008
					Genital prolapse	Leaves	Decoction	Dilshad et al., 2008
					Refrigerant	Leaves		Islam et al., 2012
					Bilious disorders	Leaves	Decoction	Khan and Hanif, 2006
					Constipation	Leaves	Decoction	Khan et al., 2015a
					Hepatitis	Leaves	Decoction	Khan et al., 2015a
					Diarrhea	Leaves	Decoction	Khattak et al., 2015
					Refrigerant	Leaves	Raw	Khattak et al., 2015
					Retention of placenta	Whole plant	Powder	Tariq et al., 2016
					Abdominal pain	Barks	Powder	Ul Hassan et al., 2014
Daphne gnidium L.	2	3	1.50	Thymelaeaceae	Skin infection	Leaves	Smoke	Aziz et al., 2018
					Testicles swellings	Leaves	Powder, Paste	Aziz et al., 2018
					Wound	Leaves, Twigs	Powder	Raziq et al., 2010
Daphne mucronata Royle	4	5	1.25	Thymelaeaceae	Repell flies	Barks	Powder	Shah et al., 2012
					Antiparasitic	Leaves, Shoots	Decoction	Sher et al., 2004
					Gastrointestinal helminthiasis	Leaves	Powder	Sindhu et al., 2010

					Pediculosis	Leaves	Powder	Sindhu et al., 2010
					Anthalmentic	Whole plant	Powder	Yousfzai et al., 2010
Daphne oleoides Schreb.	1	1	1.00	Thymelaeaceae	Anthelmintic	Flowers	Powder	Aziz et al., 2018
Daphne papyracea Wall. ex G. Don	1	9	9.00	Thymelaeaceae	Appetizer	Stems, Barks	Decoction	Ahmad et al., 2015
					Contagious pleuropneumoni a	Stems, Barks	Decoction	Ahmad et al., 2015
					Cough	Stems, Barks	Powder	Ahmad et al., 2015
					Diarrhea	Stems, Barks	Decoction	Ahmad et al., 2015
					General ailments	Stems, Barks	Decoction	Ahmad et al., 2015
					Intestinal worm	Stems, Barks	Decoction	Ahmad et al., 2015
					Jaundice	Stems, Barks	Decoction	Ahmad et al., 2015
					Mange	Stems, Barks	Decoction	Ahmad et al., 2015
					Weakness	Stems, Barks	Decoction	Ahmad et al., 2015
Datura innoxia Mill.	3	4	1.33	Solanaceae	Lice infestation	Leaves	Extract	Abbasi et al., 2013
					Delayed puberty	Flowers	Powder	Dilshad et al., 2008
					Silent estrous	Flowers	Raw	Dilshad et al., 2008
					Lice infestation	Whole plant	Paste	Tariq et al., 2014
Datura metel L.	2	4	2.00	Solanaceae	Fertility agent	Fruits	Raw	Khan and Hanif, 2006
					Snake bite	Roots	Powder	Khan and Hanif, 2006

					Common cold	Fruits	Raw	Khattak et al., 2015
					Worms	Leaves	Juice	Khattak et al., 2015
					Cough	Fruits	Raw	Khattak et al., 2015
Datura stramonium L.	1	1	1.00	Solanaceae	Increase body temperature	Leaves	Raw	Ul Hassan et al., 2014
Daucus carota L.	2	2	1.00	Apiaceae	Placenta expulsion	Leaves	Raw	Ali et al., 2017
					Retention of placenta	Seeds	Decoction	Dilshad et al., 2008
Debregeasia saeneb (Forssk.) Hepper & J.R.I.Wood	1	1	1.00	Urticaceae	Diarrhea	Leaves	Raw	Khan et al., 2012
Delphinium brunonianum Royle	1	3	3.00	Ranunculaceae	Abdominal pain	Leaves, Flowers	Raw	Ali et al., 2017
					Ectoparasites	Leaves, Flowers	Raw	Ali et al., 2017
					Mange	Leaves, Flowers	Raw	Ali et al., 2017
Delphinium denudatum Wall. ex Hook.f. & Thomson	1	1	1.00	Ranunculaceae	Antihelminthes	Rhizome	Powder	Sher et al., 2004
Desmostachya bipinnata (L.) Stapf	1	2	2.00	Poaceae	Digestive disorders	Aerial parts		Harun et al., 2017
					Dysentery	Aerial parts		Harun et al., 2017
Dichanthium annulatum (Forssk.) Stapf	1	1	1.00	Poaceae	Digestive disorders	Whole plant		Harun et al., 2017
Digera muricata (L.) Mart.	1	1	1.00	Amaranthaceae	Helminthes	Whole plant	Powder	Hussain et al., 2008
Dioscorea deltoidea Wall. ex Griseb.	1	1	1.00	Dioscoreaceae	Myiasis	Aerial parts	Powder	Sindhu et al., 2010
Diospyros lotus L.	1	1	1.00	Ebenaceae	Diarrhea	Fruits		Yousfzai et al., 2010
Dipterygium glaucum Decne.	1	1	1.00	Capparaceae	Antibacterial	Aerial parts	Decoction	Raza et al., 2014

Dodonaea viscosa Jacq.	4	8	2.00	Sapindaceae	Sex tonic	Stems, Leaves		Khattak et al., 2015
					Start heat cycle	Stems, Leaves		Khattak et al., 2015
					Wound	Leaves	Powder	Shah et al., 2012
					Wound	Leaves	Powder	Ul Hassan et al., 2014
					Anthalmentic	Leaves	Powder	Yousfzai et al., 2010
					Burn	Leaves	Powder	Yousfzai et al., 2010
					Swelling	Leaves	Powder	Yousfzai et al., 2010
					Wound	Leaves	Powder	Yousfzai et al., 2010
Dracocephalum nuristanicum Rech.f. & Edelb.	1	7	7.00	Lamiaceae	Bloat	Leaves, Flowers	Raw	Ali et al., 2017
					Constipation	Leaves, Flowers	Raw	Ali et al., 2017
					Diarrhea	Leaves, Flowers	Raw	Ali et al., 2017
					Foot diseases	Leaves, Flowers	Raw	Ali et al., 2017
					Indigestion	Leaves, Flowers	Raw	Ali et al., 2017
					Pneumonia	Leaves, Flowers	Raw	Ali et al., 2017
					Wound	Leaves, Flowers	Raw	Ali et al., 2017
Echinochloa colona (L.) Link	1	1	1.00	Poaceae	Digestive disorders	Whole plant		Harun et al., 2017
Echinochloa crus-galli (L.) P.Beauv.	1	1	1.00	Poaceae	Digestive disorders	Whole plant		Harun et al., 2017
Echinops echinatus Roxb.	1	1	1.00	Asteraceae	Tonic	Whole plant		Khattak et al., 2015
Eclipta prostrata (L.) L.	1	1	1.00	Asteraceae	Pneumonia	Aerial	Decoction	Sindhu et al., 2010

						parts		
Elaeagnus rhamnoides (L.) A.Nelson	1	2	2.00	Elaeagnaceae	Arthritis	Leaves	Raw	Ali et al., 2017
					Cough	Leaves	Raw	Ali et al., 2017
Elettaria cardamomum (L.) Maton	1	2	2.00	Zingiberaceae	Babesioses	Fruits	Powder	Khan et al., 2015a
					Weakness	Fruits	Powder	Khan et al., 2015a
Eleusine indica (L.) Gaertn.	1	1	1.00	Poaceae	Digestive disorders	Aerial parts		Harun et al., 2017
Embelia ribes Burm.f.	1	1	1.00	Primulaceae	Worm infestation	Seeds	Powder	Badar et al., 2017
Enicostema hyssopifolium (Willd.) Verd	1	1	1.00	Gentianaceae	Snake bite	Whole plant	Powder	Khan, 2009
Ephedra gerardiana Wall. ex Stapf	1	1	1.00	Ephedraceae	Dermatological problems	Stems	Powder	Ahmad et al., 2015
Eragrostis minor Host	1	1	1.00	Poaceae	Digestive disorders	Whole plant	Raw	Harun et al., 2017
Eragrostis pilosa (L.) P.Beauv.	1	1	1.00	Poaceae	Constipation	Whole plant	Raw	Harun et al., 2017
Erigeron sp.	1	2	2.00	Asteraceae	Fever	Whole plant	Powder	Abbasi et al., 2013
					Stomach collapse	Whole plant	Powder	Abbasi et al., 2013
Erigeron canadensis L.	2	5	2.50	Asteraceae	Astringent	Whole plant	Extract	Khan and Hanif, 2006
					Diuretic	Whole plant	Extract	Khan and Hanif, 2006
					Stimulant	Whole plant	Extract	Khan and Hanif, 2006
					Stomach disorders	Whole plant	Extract	Khan and Hanif, 2006
					Urinary problems	Whole plant	Powder	Shah et al., 2012
Eruca vesicaria (L.) Cav.	7	22	3.14	Brassicaceae	Ectoparasites	Seeds	Oil	Deeba et al., 2009

					Lice infestation	Seeds	Oil	Farooq et al., 2008
					Lice infestation	Seeds	Oil	Farooq et al., 2008
					Lice infestation	Seeds	Oil	Farooq et al., 2008
					Mange	Seeds	Oil	Farooq et al., 2008
					Mange	Seeds	Oil	Farooq et al., 2008
					Mange	Seeds	Oil	Farooq et al., 2008
					Tick infestation	Seeds	Oil	Farooq et al., 2008
					Helminthes	Seeds	Oil	Hussain et al., 2008
					Off feeding	Seeds	Oil	Khan et al., 2015a
					Tympney	Oil	Oil	Khan et al., 2015a
					Halitosis	Fruits		Muhammad et al., 2005
					Indigestion	Fruits		Muhammad et al., 2005
					Skin problems	Seeds		Muhammad et al., 2005
					Scabies	Seeds	Oil	Raza et al., 2014
					Skin problems	Seeds	Oil	Raza et al., 2014
					Wound	Seeds	Oil	Raziq et al., 2010
					Fly infestation	Whole plant	Oil	Sindhu et al., 2012
					Mange	Seeds	Oil	Sindhu et al., 2012
					Mange	Seeds	Oil	Sindhu et al., 2012
					Tick infestation	Oil	Powder	Sindhu et al., 2012
Eryngium biehersteinianum (M. Bieb.) Nevski	1	1	1.00	Apiaceae	Liver problems	Leaves, Stems	Raw	Aziz et al., 2018
Eucalyptus lanceolatus Dehnh.	1	1	1.00	Myrtaceae	Delivery	Whole plant	Decoction	Tariq et al., 2016
Eucalyptus camaldulensis Dehnh.	2	3	1.50	Myrtaceae	Common cold	Leaves	Raw	Abbasi et al., 2013
					Appetizers	Leaves	Decoction	Aziz et al., 2018
					Gastric ailments	Leaves	Decoction	Aziz et al., 2018
Eucalyptus globulus Labill.	1	2	2.00	Myrtaceae	Cough	Leaves	Decoction	Deeba et al., 2009

					Fever	Leaves	Decoction	Deeba et al., 2009
Euphorbia caducifolia Haines	1	1	1.00	Euphorbiaceae	Snake bite	Leaves	Milky Juice	Khan, 2009
Euphorbia cashmeriana Royle	1	1	1.00	Euphorbiaceae	Scabies	Stems, Leaves	Extract, Paste	Khan et al., 2012
Euphorbia dracunculoides Lam.	1	1	1.00	Euphorbiaceae	Galactagogue	Whole plant		Khattak et al., 2015
Euphorbia granulata Forssk.	1	2	2.00	Euphorbiaceae	Scorpion bite	Leaves	Juice	Raza et al., 2014
					Snake bite	Leaves	Juice	Raza et al., 2014
Euphorbia helioscopia L.	1	1	1.00	Euphorbiaceae	Febrifuge	Leaves	Concoction	Tariq et al., 2016
Euphorbia neriifolia L.	1	1	1.00	Euphorbiaceae	Snake bite	Latex		Mirani et al., 2016
1.00	2	2		Euphorbiaceae	Worms killer	Leaves	Decoction	Khan and Hanif, 2006
					Urinary problems	Whole plant	Decoction	Khattak et al., 2015
Euphorbia wallichii Hook.f.	1	2	2.00	Euphorbiaceae	Rashes	Stems	Latex	Abbasi et al., 2013
					Wound	Stems	Latex	Abbasi et al., 2013
Fagonia arabica L.	1	2	2.00	Zygophyllaceae	Refrigerant	Whole plant	Decoction	Khattak et al., 2015
					Vermicides	Whole plant	Decoction	Khattak et al., 2015
Fagonia cretica L.	1	1	1.00	Zygophyllaceae	Liver problems	Whole plant	Decoction	Mussarat et al., 2014
Fagonia indica Burm.f.	1	2	2.00	Zygophylaceae	Appetizer	Leaves	Raw	Abbasi et al., 2013
					Indigestion	Leaves	Raw	Abbasi et al., 2013
Fagopyrum acutatum (Lehm.) Mansf. ex K.Hammer	1	3	3.00	Polygonaceae	Antimicrobial	Aerial parts	Fodder	Ahmed and Murtaza, 2015
					diuretic and	Aerial parts	Fodder	Ahmed and Murtaza, 2015
					bactericidal	Aerial parts	Fodder	Ahmed and Murtaza, 2015
Fagopyrum esculentum Moench	1	3	3.00	Polygonaceae	Antimicrobial	Aerial	Fodder	Ahmed and Murtaza,

						parts		2015
					Diuretic	Aerial parts	Fodder	Ahmed and Murtaza, 2015
					Bactericidal	Aerial parts	Fodder	Ahmed and Murtaza, 2015
Farsetia stylosa R.Br.	1	2	2.00	Brassicaceae	Nerve tonic	Aerial parts	Decoction	Raza et al., 2014
					Refrigerant	Aerial parts	Decoction	Raza et al., 2014
Ferula assa-foetida L.	5	6	1.20	Apiaceae	Delayed puberty	Roots, Flowers	Powder	Dilshad et al., 2008
					Silent estrous	Roots, Flowers	Powder	Dilshad et al., 2008
					Helminthiasis	Fruits	Powder	Farooq et al., 2008
					Helminthes	Stems, Roots	Extract	Hussain et al., 2008
					Endoparasites	Fruits	Decoction	Mirani et al., 2016
					Helminthiasis	Fruits	Powder	Raza et al., 2014
					Gastrointestinal helminthiasis	Resin	Powder	Sindhu et al., 2010
Ferula foetida (Bunge) Regel	1	2	2.00	Apiaceae	Helminthiasis	Oil	Powder	Sindhu et al., 2012
					Helminthiasis	Resin	Decoction	Sindhu et al., 2012
Ferula narthex Boiss.	1	7	7.00	Apiaceae	Abdominal pain	Roots, Stems		Khan et al., 2015b
					Blood pressure	Roots, Stems		Khan et al., 2015b
					Digestive disorders	Roots, Stems		Khan et al., 2015b
					Mouth diseases	Roots, Stems		Khan et al., 2015b
					Nerve tonic	Roots, Stems		Khan et al., 2015b

					Respiratory	Roots, Stems		Khan et al., 2015b
					Scorpion bite	Roots, Stems		Khan et al., 2015b
Ficus benghalensis L.	2	2	1.00	Moraceae	Genital prolapse	Roots, Leaves	Decoction	Dilshad et al., 2008
					Inflammation	Latex		Ullah et al., 2017
Ficus carica L.	5	7	1.40	Moraceae	Digestive disorders	Leaves, Fruits	Raw	Aziz et al., 2018
					Retention of placenta	Leaves, Fruits	Raw	Aziz et al., 2018
					Antiseptic	Latex, Leaves		Islam et al., 2012
					Wound	Latex, Leaves		Islam et al., 2012
					Retention of placenta	Barks	Powder	Khan et al., 2015a
					Skin infection	Fruits	Juice	Tariq et al., 2016
					Retention of placenta	Barks		Ullah et al., 2017
Ficus palmata Forssk.	3	4	1.33	Moraceae	Indigestion	Leaves, Fruits	Powder	Abbasi et al., 2013
					Anorexia	Leaves	Raw	Ahmed and Murtaza, 2015
					Easy delivery	Leaves	Powder	Shah et al., 2012
					Retention of placenta	Leaves	Powder	Shah et al., 2012
Ficus religiosa L.	2	2	1.00	Moraceae	Haemorrhagic Septicemia	Leaves	Decoction	Badar et al., 2017
					Anestrus	Leaves	Decoction	Dilshad et al., 2008
Flacourtia indica (Burm.f.) Merr.	2	6	3.00	Salicaceae	Paresis	Leaves	Powder	Khan, 2009
					Rheumatism	Leaves		Khan, 2009

					Snake bite	Leaves		Khan, 2009
					Paresis	Leaves		Ullah et al., 2017
					Rheumatism	Leaves		Ullah et al., 2017
					Snake bite	Leaves		Ullah et al., 2017
Flueggea leucopyrus Willd.	1	3	3.00	Phyllanthaceae	Myiasis	Leaves	Poultice	Khan, 2009
					Wound	Leaves	Poultice	Khan, 2009
Foeniculum vulgare Mill.	20	51	2.55	Apiaceae	Diarrhea	Aerial parts	Powder	Abbasi et al., 2013
					Indigestion	Aerial parts	Decoction	Abbasi et al., 2013
					Abdominal pain	Seeds	Decoction	Ali et al., 2017
					Bloat	Seeds	Decoction	Ali et al., 2017
					Colic	Seeds	Decoction	Ali et al., 2017
					Diarrhea	Seeds	Decoction	Ali et al., 2017
					Stomach pain	Seeds	Decoction	Ali et al., 2017
					Tympany	Seeds	Decoction	Ali et al., 2017
					Appetizer	Aerial parts	Decoction	Aziz et al., 2018
					Diarrhea	Aerial parts	Decoction	Aziz et al., 2018
					Indigestion	Leaves	Powder	Badar et al., 2017
					Anorexia	Seeds	Powder	Deeba et al., 2009
					Bloat	Seeds	Powder	Deeba et al., 2009
					Diarrhea	Seeds	Powder	Deeba et al., 2009
					Endoparasites	Seeds	Raw	Deeba et al., 2009
					Uterine prolapse	Seeds	Decoction	Deeba et al., 2009
					Anestrus	Seeds	Raw	Dilshad et al., 2008
					Mastitis	Seeds	Cooked	Dilshad et al., 2010
					Helminthes	Seeds	Powder	Hussain et al., 2008
					Carminative	Whole plant		Islam et al., 2012
					Diuretic	Whole		Islam et al., 2012

						plant		
					Fever	Whole plant		Islam et al., 2012
					Purgative	Whole plant		Islam et al., 2012
					Anthelmintic	Fruits	Raw	Khan and Hanif, 2006
					Appetizer	Fruits	Raw	Khan and Hanif, 2006
					Purgative	Fruits	Raw	Khan and Hanif, 2006
					Stomach disorders	Fruits	Raw	Khan and Hanif, 2006
					Worm infestation	Fruits	Raw	Khan and Hanif, 2006
					Constipation	Seeds	Powder	Khan et al., 2015a
					Hepatitis	Seeds	Powder	Khan et al., 2015a
					Mastitis	Seeds	Powder	Khan et al., 2015a
					Off feeding	Seeds	Powder	Khan et al., 2015a
					Bloat	Seeds	Decoction	Mirani et al., 2014
					Tympany	Seeds	Decoction	Mirani et al., 2014
					Bloat	Seeds	Decoction	Mirani et al., 2016
					Tympany	Seeds	Decoction	Mirani et al., 2016
					Digestive disorders	Seeds		Muhammad et al., 2005
					Halitosis	Fruits		Muhammad et al., 2005
					Indigestion	Fruits		Muhammad et al., 2005
					Indigestion	Seeds	Powder	Mussarat et al., 2014
					Mastitis	Seeds	Concoction	Mussarat et al., 2014
					Stomach	Seeds	Powder	Mussarat et al., 2014

					problems			
					Cough	Fruits	Powder	Raza et al., 2014
					Respiratory	Fruits	Powder	Raza et al., 2014
					Helminthiasis	Leaves	Extract	Sindhu et al., 2012
					Mastitis	Seeds	Powder	Tariq et al., 2016
					Carminative	Fruits		Ullah et al., 2017
					Galactagogue	Fruits		Ullah et al., 2017
					Galactagogue	Fruits	Raw	Yousfzai et al., 2010
					Haemorhagic septicaemia	Fruits	Raw	Yousfzai et al., 2010
					Pneumonia	Fruits	Raw	Yousfzai et al., 2010
Fumaria indica (Hausskn.) Pugsley	8	15	1.87	Papaveraceae	Diarrhea	Whole plant	Raw	Abbasi et al., 2013
					Constipation	Leaves	Raw	Badar et al., 2017
					Constipation	Whole plant		Islam et al., 2012
					Fever	Whole plant		Islam et al., 2012
					Urinary problems	Whole plant		Islam et al., 2012
					Mastitis	Whole plant	Decoction	Khan et al., 2015a
					Diarrhea	Whole plant		Sher et al., 2004
					Fever	Whole plant		Sher et al., 2004
					Blood purifier	Whole plant		Sher et al., 2004
					Cooling agent	Whole plant		Sher et al., 2004
					Antipyretic	Whole plant	Decoction	Tariq et al., 2016

					Wound	Whole plant	Decoction	Tariq et al., 2016
					Inflammation	Whole plant		Ullah et al., 2017
					Skin diseases	Whole plant		Ullah et al., 2017
					Refrigerant	Whole plant	Decoction	Yousfzai et al., 2010
Gentiana olivieri Griseb.	1	2	2.00	Gentianaceae	Bloat	Leaves, Flowers	Paste	Ali et al., 2017
					Hepatic problems	Leaves, Flowers	Paste	Ali et al., 2017
Geranium wallichianum D.Don ex Sweet	4	5	1.25	Geraniaceae	Abscess	Rhizome s	Decoction	Khuroo et al., 2007
					Inflammation	Rhizome s	Decoction	Khuroo et al., 2007
					Galactagogue	Rhizome	Powder	Sher et al., 2004
					Galactagogue	Rhizome s	Powder	Ul Hassan et al., 2014
					Galactagogue	Rhizome s	Powder	Yousfzai et al., 2010
Gisekia pharnaceoides L.	1	1	1.00	Gisekiaceae	Antipyretics	Aerial parts		Raza et al., 2014
Glycyrrhiza glabra L.	5	8	1.60	Fabaceae	Galactagogue	Roots	Raw	Aziz et al., 2018
					Sex tonic	Roots	Raw	Aziz et al., 2018
					Cough	Roots	Powder	Deeba et al., 2009
					Fever	Roots	Powder	Deeba et al., 2009
					Cough	Rhizome s	Powder	Khan et al., 2015a
					Off feeding	Rhizome s	Powder	Khan et al., 2015a
					Galactogague	Seeds	Powder	Khuroo et al., 2007

					Cough	Stems	Powder	Mussarat et al., 2014
Gossypium arboreum L.	4	1	0.25	Malvaceae	Galactagogue	Whole plant	Powder	Aziz et al., 2018
Gossypium hirsutum L.	3	4	1.33	Malvaceae	Delayed puberty	Seeds	Raw	Dilshad et al., 2008
					Silent estrous	Seeds	Raw	Dilshad et al., 2008
					Mastitis	Flowers	Decoction	Dilshad et al., 2010
					Diarrhea	Buds	Powder	Mussarat et al., 2014
Gossypium indicum Lam.	1	4	4.00	Malvaceae	Cough	Fruits, Roots	Powder	Khan et al., 2015a
					Fainting	Fruits, Roots	Powder	Khan et al., 2015a
					Pneumonia	Fruits, Roots	Powder	Khan et al., 2015a
					Retention of placenta	Fruits, Roots	Powder	Khan et al., 2015a
Grewia asiatica L.	1	3	3.00	Malvaceae	Bone fracture	Roots	Raw	Khan and Hanif, 2006
					After birth release	Stems	Decoction	Khan and Hanif, 2006
					Easy delivery	Leaves	Decoction	Khan and Hanif, 2006
					Wound	Roots	Raw	Khan and Hanif, 2006
					Gastrointestinal helminthiasis	Barks	Powder	Sindhu et al., 2010
Grewia optiva J.R.Drumm. ex Burret	3	3	1.00	Malvaceae	Wound	Whole plant	Powder	Aziz et al., 2018
					Galactagogue	Barks	Powder	Shah et al., 2012
					Tonic	Stems Barks	Decoction	Ul Hassan et al., 2014
Grewia villosa Willd.	1	2	2.00	Malvaceae	Diarrhea	Whole plant	Raw	Raza et al., 2014

					Dysentery	Whole plant	Raw	Raza et al., 2014
Gymnosporia royleana Wall. ex M.A.Lawson	1	1	1.00	Celasteraceae	Spleen pain	Roots	Decoction	Khan and Hanif, 2006
Haloxylon recurvum Bunge ex Boiss.	1	1	1.00	Amaranthaceae	Helminthiasis	Aerial parts		Raza et al., 2014
Haloxylon salicornicum (Moq.) Bunge ex Boiss.	2	7	3.50	Amaranthaceae	Fly infestation	Aerial parts	Smoke	Farooq et al., 2008
					Lice infestation	Aerial parts	Decoction	Farooq et al., 2008
					Lice infestation	Whole plant	Ash	Farooq et al., 2008
					Mange	Aerial parts	Powder	Farooq et al., 2008
					Myiasis	Aerial parts	Raw	Farooq et al., 2008
					Tick infestation	Whole plant	Ash	Farooq et al., 2008
					Helminthiasis	Aerial parts		Raza et al., 2014
Hedera nepalensis K.Koch	2	2	1.00	Araliaceae	Remove leeches	Leaves	Powder	Abbasi et al., 2013
					Remove leeches	Leaves	Infusion	Tariq et al., 2014
Heliotropium crispum Desf.	1	1	1.00	Boraginaceae	Otitis	Leaves	Raw	Raza et al., 2014
Heliotropium eichwaldii Steud.	1	1	1.00	Boraginaceae	Ear pain	Leaves	Raw	Khan, 2009
Helleborus niger L.	1	1	1.00	Ranunculaceae	Helminthes	Aerial parts	Powder	Hussain et al., 2008
Heracleum candicans Wall. ex DC.	1	1	1.00	Apiaceae	Sex tonic	Roots	Raw	Aziz et al., 2018
Heracleum pinnatum C.B. Clarke	1	2	2.00	Apiaceae	Abdominal worms	Leaves	Raw	Ali et al., 2017
					Endoparasites	Leaves	Raw	Ali et al., 2017

Heteropogon contortus (L.) P.Beauv. ex Roem. & Schult.	1	1	1.00	Poaceae	Digestive disorders	Aerial parts		Harun et al., 2017
Hibiscus cannabinus L.	1	3	3.00	Malvaceae	Antipyretic	Aerial parts	Decoction	Raza et al., 2014
					Itching	Aerial parts	Decoction	Raza et al., 2014
					Skin problems	Aerial parts	Paste	Raza et al., 2014
Hibiscus rosa-sinensis L.	1	1	1.00	Malvaceae	Galactagogue	Leaves	Raw	Khan et al., 2015a
Himalaiella heteromalla (D.Don) Raab-Straube	4	6	1.50	Asteraceae	Blood purification	Whole plant	Powder	Abbasi et al., 2013
					Edema	Seeds	Raw	Abbasi et al., 2013
					Carminative	Seeds	Raw	Khan and Hanif, 2006
					Horse bite	Seeds	Raw	Khan and Hanif, 2006
					Flatulence	Seeds	Raw	Khan et al., 2012
					Galactagogue	Rhizome s	Powder	Shah et al., 2012
Hordeum vulgare L.	6	10	1.67	Poaceae	Diarrhea	Seeds	Powder	Ali et al., 2017
					Genital prolapse	Seeds	Powder	Aziz et al., 2018
					Diarrhea	Seeds	Powder	Deeba et al., 2009
					Foot diseases	Seeds	Decoction	Deeba et al., 2009
					Mouth diseases	Seeds	Decoction	Deeba et al., 2009
					Uterine prolapse	Seeds	Decoction	Deeba et al., 2009
					Genital prolapse	Seeds	Powder	Dilshad et al., 2008
					Weakness	Seeds	Raw	Khan et al., 2015a
					Cough	Seeds	Powder	Mussarat et al., 2014
					Weakness	Seeds		Ullah et al., 2017
Hyoscyamus niger L.	2	8	4.00	Solanaceae	Abdominal pain	Leaves, Seeds		Khan et al., 2015b

					Anti-spasmodic	Leaves, Seeds		Khan et al., 2015b
					Mouth diseases	Leaves, Seeds		Khan et al., 2015b
					Respiratory	Leaves, Seeds		Khan et al., 2015b
					Narcotic	Leaves, Seeds		Khan et al., 2015b
					Sedative	Leaves, Seeds		Khan et al., 2015b
					Urinary problems	Leaves, Seeds		Khan et al., 2015b
					Wound	Leaves	Decoction	Yousfzai et al., 2010
Hypericum perforatum L.	3	3	1.00	Hypericaceae	Healing wounds	Roots	Powder	Sher et al., 2004
					Increase body temperature	Whole plant	Decoction	Ul Hassan et al., 2014
					Wound	Whole plant	Powder	Yousfzai et al., 2010
Inula royleana DC.	1	3	3.00	Asteraceae	Inflammation	Flowers	Decoction	Khuroo et al., 2007
					Throat sores	Flowers	Decoction	Khuroo et al., 2007
					Wound	Flowers	Decoction	Khuroo et al., 2007
Impatiens scabrida DC.	1	2	2.00	Balsaminaceae	Laxative			Ahmed and Murtaza, 2015
					Diuretic			Ahmed and Murtaza, 2015
Ipomoea carnea Jacq.	1	1	1.00	Convolvulaceae	Fever	Leaves	Powder	Mussarat et al., 2014
Isodon rugosus (Wall. ex Benth.) Codd	3	3	1.00	Lamiaceae	Flea Infestation	Aerial parts	Infusion	Ahmed and Murtaza, 2015
					Retention of placenta	Whole plant	Decoction	Dilshad et al., 2008
					Throat	Leaves	Powder	Khuroo et al., 2007

					infections			
Jasminum humile L.	1	2	2.00	Oleaceae	Helminthiasis	Leaves	Extract	Sindhu et al., 2012
					Helminthiasis	Leaves	Powder	Sindhu et al., 2012
Juglans regia L.	3	4	1.33	Juglandaceae	Placenta expulsion	Leaves	Raw	Aziz et al., 2018
					Fainting	Barks	Powder	Khan et al., 2015a
					Pneumonia	Barks	Powder	Khan et al., 2015a
					Placenta expulsion	Leaves	Raw	Ul Hassan et al., 2014
Justicia adhatoda L.	6	8	1.33	Acanthaceae	Stomach problems	Leaves	Infusion	Abbasi et al., 2013
					Fever	Leaves	Infusion	Abbasi et al., 2013
					Dehydration	Leaves	Infusion	Abbasi et al., 2013
					Dysentry	Leaves	Raw	Abbasi et al., 2013
					Febrifuge	Leaves, Flowers		Islam et al., 2012
					Rashes	Leaves, Flowers		Islam et al., 2012
					Worms	Roots, Leaves	Decoction	Khan and Hanif, 2006
					Antiseptic	Wood	Ash	Khan et al., 2012
					Antihelmintic	Roots, Leaves	Decoction	Khan et al., 2012
					Inflammation	Leaves	Powder	Shah et al., 2012
					Wound	Leaves	Powder	Shah et al., 2012
					Increase body temperature	Leaves		Yousfzai et al., 2010
Lactuca brunoniana (DC.) Wall. ex C.B.Clarke	1	1	1.00	Asteraceae	Pinworms	Whole plant	Fodder	Ahmed and Murtaza, 2015
Lagenaria siceraria (Molina) Standl.	3	3	1.00	Asteraceae	Helminthes	Leaves	Powder	Hussain et al., 2008
					Tonic	Leaves		Khattak et al., 2015
					Galactagogue	Whole	Powder	Tariq et al., 2014

						plant		
Lamium amplexicaule L.	1	1	1.00	Lamiaceae	Helminthiasis	Leaves	Powder	Raza et al., 2014
Launaea nudicaulis (L.) Hook.f.	1	1	1.00	Asteraceae	Helminthiasis	Aerial parts	Raw	Raza et al., 2014
Launaea procumbens (Roxb.) Ramayya & Rajagopal	4	5	1.25	Asteraceae	Skin infection	Leaves	Raw	Abbasi et al., 2013
					Galactagogue	Whole plant	Raw	Aziz et al., 2018
					Tonic	Whole plant	Raw	Aziz et al., 2018
					Tonic	Leaves		Khattak et al., 2015
					Galactagogue	Leaves	Powder	Yousfzai et al., 2010
Lawsonia inermis L.	4	6	1.50	Lythraceae	Genital prolapse	Leaves	Raw	Dilshad et al., 2008
					Mastitis	Leaves	Powder	Mirani et al., 2014
					Bone fracture	Leaves	Powder	Mirani et al., 2016
					Mastitis	Leaves	Powder	Mirani et al., 2016
					Tick infestation	Leaves	Infusion	Sindhu et al., 2010
					Tick infestation	Leaves	Powder	Sindhu et al., 2010
Lens culinaris Medik.	3	4	1.33	Fabaceae	Delayed puberty	Seeds	Raw	Dilshad et al., 2008
					Silent estrous	Seeds	Raw	Dilshad et al., 2008
					Placenta expulsion	Seeds	Powder	Khan et al., 2015a
					Broken horns	Seeds		Ullah et al., 2017
Leontopodium leontopodinum (DC.) Hand.-Mazz.	1	4	4.00	Asteraceae	Bloat	Seeds		Ali et al., 2017
					Constipation	Seeds		Ali et al., 2017
					Diarrhea	Seeds		Ali et al., 2017
					Tympany	Seeds		Ali et al., 2017
Lepidium didymum L.	1	1	1.00	Brassicaceae	Maggots from wound	Whole plant	Powder	Shah et al., 2012
Lepidium sativum L.	11	20	1.81	Brassicaceae	Intestinal	Leaves	Powder	Ali et al., 2017

					problems			
					Galactagogue	Seeds	Raw	Badar et al., 2017
					Haemorrhagic septicemia	Seeds	Raw	Badar et al., 2017
					Genital prolapse	Seeds	Raw	Dilshad et al., 2008
					Mastitis	Seeds	Decoction	Dilshad et al., 2010
					Fever	Seeds	Decoction	Khan et al., 2015a
					Fever	Seeds	Powder	Khan et al., 2015a
					Off feeding	Seeds	Powder	Khan et al., 2015a
					Tympany	Seeds	Decoction	Khan et al., 2015a
					Weakness	Seeds	Decoction	Khan et al., 2015a
					Halitosis	Fruits		Muhammad et al., 2005
					Indigestion	Fruits		Muhammad et al., 2005
					Fever	Seeds	Powder	Shah et al., 2012
					Flatulence	Seeds	Powder	Shah et al., 2012
					Gastric problems	Seeds	Decoction	Sher et al., 2004
					Lice infestation	Seeds	Powder	Sindhu et al., 2012
					Analgesic	Seeds		Ullah et al., 2017
					Galactagogue	Seeds		Ullah et al., 2017
					Flatulence	Seeds		Yousfzai et al., 2010
					Purgative	Seeds		Yousfzai et al., 2010
Leptadenia pyrotechnica (Forssk.) Decne.	1	1	1.00	Apocynaceae	Snake bite	Stems	Raw	Mirani et al., 2016
Leptopus cordifolius Decne.	2	2	1.00	Phyllanthaceae	Anthelmintic	Leaves, Root	Powder	Ahmed and Murtaza, 2015
					Diarrhea			Khan et al., 2012
Linum usitatissimum L.	5	10	2.00	Linaceae	Delayed puberty	Seeds	Raw	Dilshad et al., 2008
					Retention of	Seeds	Raw	Dilshad et al., 2008

					placenta			
					Silent estrous	Seeds	Raw	Dilshad et al., 2008
					Mastitis	Seeds, Fruits	Raw	Dilshad et al., 2010
					Galactagogue	Seeds	Raw	Khan and Hanif, 2006
					Cough	Seeds	Decoction	Khan et al., 2015a
					Weakness	Seeds	Powder	Khan et al., 2015a
					Fever	Seeds		Ullah et al., 2017
					Galactagogue	Seeds		Ullah et al., 2017
					Weakness	Seeds		Ullah et al., 2017
Litsea monopetala (Roxb.) Pers.	1	1	1.00	Lauraceae	Jaundice	Wood	Decoction	Ahmad et al., 2015
Lolium temulentum L.	1	1	1.00	Poaceae	Nervous disorders	Leaves		Harun et al., 2017
Lonicera asperifolia Hook. f. & Thomson	1	1	1.00	Caprifoliaceae	Indigestion	Leaves	Raw	Ali et al., 2017
Lotus corniculatus L.	1	1	1.00	Fabaceae	Genital problems	Leaves, Stems	Powder	Aziz et al., 2018
Lycium depressum Stocks	2	2	1.00	Solanaceae	Respiratory	Barks	Powder	Khan, 2009
					Expectorant	Fruits		Ullah et al., 2017
Mallotus philippensis (Lam.) Müll.Arg.	10	13	1.30	Euphorbiaceae	Intestinal worm	Fruits	Powder	Abbasi et al., 2013
					Worm infestation	Fruits		Badar et al., 2017
					Helminthiasis	Fruits	Powder	Farooq et al., 2008
					Helminthiasis	Flowers	Powder	Hussain et al., 2008
					Anthelmentic	Fruits, Leaves		Islam et al., 2012
					Laxative	Fruits, Leaves		Islam et al., 2012
					Worms	Fruits		Khan and Hanif, 2006

					Abdominal worms	Fruits	Powder	Khan et al., 2012
					Worm infestation	Fruits	Powder	Khan et al., 2015a
					Helminthiasis	Leaves	Powder	Sindhu et al., 2012
					Increase body temperature	Fruits	Powder	Ul Hassan et al., 2014
					Diarrhea	Fruits	Powder	Yousfzai et al., 2010
					Vermicides	Fruits	Powder	Yousfzai et al., 2010
Malus domestica Borkh.	1	1	1.00	Rosaceae	Diarrhea	Fruits	Powder	Ali et al., 2017
Malva neglecta Wallr.	3	4	1.33	Malvaceae	Flatulence	Roots	Extract	Aziz et al., 2018
					Digestive disorders	Leaves, Stems, Seeds		Islam et al., 2012
					Food poisoning	Leaves, Stems, Seeds		Islam et al., 2012
					Flatulence	Leaves	Decoction	Ul Hassan et al., 2014
Malva sylvestris L.	1	1	1.00	Malvaceae	Respiratory	Leaves	Decoctionn	Khuroo et al., 2007
Mangifera indica L.	6	7	1.17	Anacardiaceae	Mouth diseases	Fruits	Raw	Abbasi et al., 2013
					Gastrointestinal problems	Seeds	Raw	Aziz et al., 2018
					Retention of placenta	Leaves		Dilshad et al., 2008
					Helminthes	Leaves	Powder	Hussain et al., 2008
					Diarrhea	Seeds	Powder	Khan et al., 2015a
					Diarrhea	Seeds	Powder	Mussarat et al., 2014
					Pain in muscles	Seeds	Powder	Mussarat et al., 2014
Medicago laciniata (L.) Mill.	1	1	1.00	Zygophyllaceae	Galactagogue	Whole plant		Khattak et al., 2015

Medicago monantha (C.A.Mey.) Trautv.	1	1	1.00	Fabaceae	Galactagogue	Whole plant		Khattak et al., 2015
Medicago sativa L.	1	1	1.00	Fabaceae	Helminthes	Aerial parts	Powder	Hussain et al., 2008
Melia azedarach L.	19	37	1.94	Meliaceae	Flatulance	Leaves	Raw	Abbasi et al., 2013
					Foot diseases	Leaves	Raw	Abbasi et al., 2013
					Indigestion	Leaves	Raw	Abbasi et al., 2013
					Mouth diseases	Leaves	Raw	Abbasi et al., 2013
					Rashes	Fruit rinds	Infusion	Abbasi et al., 2013
					Skin infections	Leaves	Raw	Abbasi et al., 2013
					Anthelmintic	Seed	Powder	Ahmed and Murtaza, 2015
					Diarrhea	Leaves	Powder	Aziz et al., 2018
					Gastric ailments	Leaves	Powder	Aziz et al., 2018
					Diarrhea	Fruits	Powder	Deeba et al., 2009
					Genital prolapse	Seeds	Powder	Dilshad et al., 2008
					Anthelmintic	Leaves, Fruits		Islam et al., 2012
					Febrifuge	Leaves, Fruits		Islam et al., 2012
					Gastric ailments	Leaves, Fruits		Islam et al., 2012
					Appetizer	Seeds	Powder	Khan and Hanif, 2006
					Cough	Seeds	Powder	Khan and Hanif, 2006
					Fever	Leaves	Paste	Khan and Hanif, 2006
					Flatulence	Seeds	Powder	Khan and Hanif, 2006
					Flatulence	Seeds	Powder	Khan et al., 2012

					Bloat	Leaves	Powder	Khan et al., 2015a
					Constipation	Leaves	Powder	Khan et al., 2015a
					Respiratory	Fruits	Raw	Khattak et al., 2015
					Stop irritation	Fruits	Raw	Khattak et al., 2015
					Stomach problems	Leaves	Powder	Mussarat et al., 2014
					Galactagogue	Leaves, Seeds	Powder	Shah et al., 2012
					Gastric problems	Leaves	Powder	Sher et al., 2004
					Tick infestation	Fruits	Powder	Sindhu et al., 2012
					Flatulence	Leaves	Powder	Tariq et al., 2014
					Ectoparasites	Leaves, Fruits	Decoction	Tariq et al., 2016
					Fever	Leaves, Fruits	Decoction	Tariq et al., 2016
					Gastric ailments	Leaves, Fruits	Decoction	Tariq et al., 2016
					Snake bite	Leaves	Raw	Ul Hassan et al., 2014
					Flatulence	Barks	Powder	Ul Hassan et al., 2014
					Refrigerant	Fruits		Ullah et al., 2017
					Kill lices	Fruits	Powder	Yousfzai et al., 2010
					Sore throat	Fruits	Powder	Yousfzai et al., 2010
Mentha arvensis L.	6	11	1.83	Lamiaceae	Abdominal pain	Leaves	Raw	Ali et al., 2017
					Analgesic	Leaves	Raw	Ali et al., 2017
					Colic	Leaves	Raw	Ali et al., 2017
					Constipation	Leaves	Raw	Ali et al., 2017
					Stomach problems	Leaves	Raw	Ali et al., 2017
					Anorexia	Leaves	Raw	Badar et al., 2017

					Colic	Leaves	Raw	Badar et al., 2017
					Genital prolapse	Roots	Powder	Dilshad et al., 2008
					Ectoparasite	Leaves	Powder	Shah et al., 2012
					Ectoparasite	Leaves	Paste	Tariq et al., 2014
					Ectoparasite	Leaves	Decoction	Tariq et al., 2016
Mentha canadensis L.	1	4	4.00	Lamiaceae	Constipation	Leaves	Raw	Ali et al., 2017
					Placenta expulsion	Leaves	Raw	Ali et al., 2017
					Pneumonia	Leaves	Raw	Ali et al., 2017
					Wound	Leaves	Raw	Ali et al., 2017
Mentha longifolia (L.) L.	11	31	2.81	Lamiaceae	Abdominal pain	Leaves	Infusion	Ahmad et al., 2015
					Refrigerant	Leaves	Infusion	Ahmad et al., 2015
					Anti-cholera,	Aerial parts	Infusion	Ahmed and Murtaza, 2015
					Anti-dyspepsia,	Aerial parts	Infusion	Ahmed and Murtaza, 2015
					Anti-emetic	Aerial parts	Infusion	Ahmed and Murtaza, 2015
					Abdominal pain	Whole plant	Powder	Aziz et al., 2018
					Diarrhea	Whole plant	Powder	Aziz et al., 2018
					Placenta expulsion	Whole plant	Powder	Aziz et al., 2018
					Tonic	Whole plant	Powder	Aziz et al., 2018
					Bloat	Leaves	Powder	Deeba et al., 2009
					Diarrhea	Leaves	Powder	Deeba et al., 2009
					Febrifuge	Aerial parts		Islam et al., 2012
					Stomachic problems	Aerial parts		Islam et al., 2012

					Off feeding	Leaves	Powder	Khan et al., 2015a
					Galactagogue	Roots	Decoction	Shah et al., 2012
					Asthma	Leaves	Juice	Shedayi et al., 2014
					Astringent	Leaves	Juice	Shedayi et al., 2014
					Common cold	Leaves	Juice	Shedayi et al., 2014
					Cough	Leaves	Juice	Shedayi et al., 2014
					Fever	Leaves	Juice	Shedayi et al., 2014
					Headache	Leaves	Juice	Shedayi et al., 2014
					Indigestion	Leaves	Juice	Shedayi et al., 2014
					Migraines	Leaves	Juice	Shedayi et al., 2014
					Profuse mucus discharge	Leaves	Juice	Shedayi et al., 2014
					Respiratory	Leaves	Juice	Shedayi et al., 2014
					Respiratory	Leaves	Juice	Shedayi et al., 2014
					Rheumatism	Leaves	Juice	Shedayi et al., 2014
					Stomachic	Leaves	Juice	Shedayi et al., 2014
					Flatulence	Leaves	Powder	Sher et al., 2004
					Diarrhea	Roots	Decoction	Ul Hassan et al., 2014
					Off feeding	Leaves		Ullah et al., 2017
Mentha royleana Wall. ex Benth.	1	2	2.00	Lamiaceae	Constipation	Leaves	Raw	Ali et al., 2017
					Wound	Leaves	Raw	Ali et al., 2017
Mentha spicata L.	3	4	1.33	Lamiaceae	Gastric ailments	Leaves, Stems	Powder	Aziz et al., 2018
					Tympany	Leaves	Decoction	Mirani et al., 2014
					Bloat	Leaves	Decoction	Mirani et al., 2016
					Tympany	Leaves	Decoction	Mirani et al., 2016
Mollugo nudicaulis Lam.	1	1	1.00	Molluginaceae	Abscesses	Leaves	Paste	Khan, 2009
Momordica charantia L.	1	1	1.00	Cucurbitaceae	Fever	Whole plant	Infusion	Khan and Hanif, 2006
Momordica dioica Roxb. ex Willd.	1	3	3.00	Cucurbitaceae	Respiratory problems	Roots	Powder, Infusion	Khan and Hanif, 2006

					Wound	Roots	Powder	Khan and Hanif, 2006
Morus alba L.	4	5	1.25	Moraceae	Haemorrhagic Septicemia	Leaves	Decoction	Badar et al., 2017
					Laxative	Leaves	Raw	Islam et al., 2012
					Tonic	Leaves	Raw	Islam et al., 2012
					Constipation	Leaves	Raw	Khan et al., 2015a
					Laxative	Leaves	Powder	Tariq et al., 2014
Morus indica L.	1	1	1.00	Moraceae	Anestrus	Leaves	Raw	Dilshad et al., 2008
Morus nigra L.	6	10	1.67	Moraceae	Laxative	Leaves, Fruits	Raw	Islam et al., 2012
					Tonic	Leaves, Fruits	Raw	Islam et al., 2012
					Diarrhea	Leaves	Decoction	Khan and Hanif, 2006
					Fertility agent	Leaves	Powder	Khan and Hanif, 2006
					Cough	Fruits	Raw	Khan et al., 2015a
					Laxative	Leaves	Powder	Tariq et al., 2014
					Tonic	Leaves	Powder	Tariq et al., 2014
					Skin infections	Fruits	Juice	Tariq et al., 2016
					Fertility agent	Fruits		Ullah et al., 2017
					Scorpion bite	Fruits		Ullah et al., 2017
Musa × *paradisiaca* L.	5	8	1.60	Musaceae	Genital prolapse	Pulp of Stems	Powder	Dilshad et al., 2008
					Helminthes	Leaves	Powder	Hussain et al., 2008
					Fever	Leaves	Extract	Khan and Hanif, 2006
					Stabilize the pregnancy	Leaves	Extract	Khan and Hanif, 2006
					Tonic	Leaves	Extract	Khan and Hanif, 2006

					Retention of placenta	Stems	Juice	Khan et al., 2015a
					Diarrhea	Fruits	Raw	Mirani et al., 2014
					Dysentery	Fruits	Raw	Mirani et al., 2014
Myristica fragrans Houtt.	2	2	1.00	Myristicaceae	Dystokia	Seeds	Powder	Dilshad et al., 2008
					Colic	Fruits, Leaves	Decoction	Khan et al., 2015a
Myrsine africana L.	1	2	2.00	Primulaceae	Indigestion	Leaves	Raw	Abbasi et al., 2013
					Worms	Leaves	Raw	Abbasi et al., 2013
Myrtus communis L.	3	3	1.00	Myrtaceae	Digestive disorders	Leaves	Decoction	Aziz et al., 2018
					Blood in milk	Leaves	Decoction	Khan et al., 2015a
					Diarrhea	Leaves	Decoction	Ul Hassan et al., 2014
Nannorrhops ritchieana (Griff.) Aitch.	3	7	2.33	Arecaceae	Foot diseases	Leaves	Raw	Ahmad et al., 2015
					Mouth diseases	Leaves	Raw	Ahmad et al., 2015
					Multisystem	Leaves	Raw	Ahmad et al., 2015
					Foot diseases	Leaves	Raw	Aziz et al., 2018
					Mouth diseases	Leaves	Raw	Aziz et al., 2018
					Gastric ailments	Leaves	Decoction	Tariq et al., 2016
					Gastric ailments	Leaves	Decoction	Tariq et al., 2016
Narcissus tazetta L.	1	1	1.00	Amaryllidaceae	Retention of placenta	Leaves	Decoction	Aziz et al., 2018
Nepeta laevigata (D.Don) Hand.-Mazz.	1	2	2.00	Lamiaceae	Intestinal disorders	Inflorescence	Decoctionn	Khuroo et al., 2007
					Urinary problems	Inflorescence	Decoctionn	Khuroo et al., 2007
Nerium oleander L.	3	3	1.00	Apocynaceae	Skin problems	Leaves	Decoction	Aziz et al., 2018
					Helminthiasis	Leaves	Powder	Sindhu et al., 2012
					Pain of stomach	Whole plant	Concoction	Tariq et al., 2014
Nicotiana rustica L.	3	4	1.33	Solanaceae	Wound	Leaves	Powder	Khan et al., 2015a

					Ectoparasites	Leaves	Extract	Ul Hassan et al., 2014
					Remove the ticks	Leaves	Extract	Ul Hassan et al., 2014
					Wound	Leaves		Ullah et al., 2017
Nicotiana tabacum L.	9	25	2.78	Solanaceae	Antiparasitic	Twigs	Decoction	Aziz et al., 2018
					Anti-parasitic	Leaves	Decoction	Aziz et al., 2018
					Diarrhea	Leaves	Smoke	Badar et al., 2017
					Worm infestation	Leaves	Somke	Badar et al., 2017
					Helminthiasis	Leaves	Decoction	Farooq et al., 2008
					Lice infestation	Leaves	Decoction	Farooq et al., 2008
					Mange	Leaves	Raw	Farooq et al., 2008
					Mange	Leaves	Decoction	Farooq et al., 2008
					Myiasis	Leaves	Raw	Farooq et al., 2008
					Tick infestation	Leaves	Decoction	Farooq et al., 2008
					Helminthes	Leaves	Decoction	Hussain et al., 2008
					Colic	Leaves	Decoction	Khan et al., 2015a
					Wound	Leaves	Powder	Khan et al., 2015a
					Ectoparasites	Leaves	Decoction	Mirani et al., 2014
					Myiasis	Leaves	Powder	Mirani et al., 2014
					Ectoparasites	Leaves	Decoction	Mirani et al., 2016
					Myiasis	Leaves	Powder	Mirani et al., 2016
					Snake bite	Leaves	Powder	Mirani et al., 2016
					Skin problems	Leaves		Muhammad et al., 2005
					Helminthiasis	Leaves	Powder	Sindhu et al., 2012
					Lice infestation	Leaves	Decoction	Sindhu et al., 2012
					Mange	Leaves	Decoction	Sindhu et al., 2012
					Tick infestation	Leaves	Decoction	Sindhu et al., 2012
					Indigestion	Leaves		Ullah et al., 2017

					Wound	Leaves		Ullah et al., 2017
Nigella sativa L.	4	5	1.25	Ranunculaceae	Tonic	Seeds	Decoction	Aziz et al., 2018
					Delayed puberty	Seeds		Dilshad et al., 2008
					Silent estrous	Seeds		Dilshad et al., 2008
					Mastitis	Seeds	Decoction	Dilshad et al., 2010
					Mastitis	Seeds	Decoction	Mussarat et al., 2014
Ocimum basilicum L.	4	7	1.75	Lamiaceae	Skin problems	Seeds, Leaves	Decoction	Aziz et al., 2018
					Diarrhea	Leaves	Decoction	Mirani et al., 2016
					Dysentery	Leaves	Decoction	Mirani et al., 2016
					Helminthiasis	Leaves	Powder	Sindhu et al., 2012
					Helminthiasis	Leaves	Extract	Sindhu et al., 2012
					Helminthiasis	Leaves	Powder	Sindhu et al., 2012
					Gastric ailments	Leaves	Decoction	Tariq et al., 2014
Olea europaea L.	2	3	1.50	Oleaceae	Bone fracture	Fruits	Oil	Aziz et al., 2018
					Inflammation	Fruits	Oil	Aziz et al., 2018
					Colic	Fruits	Oil	Khan et al., 2015a
Olea europaea subsp. *cuspidata* (Wall. & G.Don) Cif.	4	10	2.50	Oleaceae	Indigestion	Fruits	Extract	Abbasi et al., 2013
					Bone fracture	Barks	Raw	Ahmad et al., 2015
					Gastric ailments	Leaves	Raw	Ahmad et al., 2015
					General ailments	Leaves	Decoction	Ahmad et al., 2015
					Medicinal food	Leaves	Raw	Ahmad et al., 2015
					Worms in abdomin	Leaves,	Raw	Ahmad et al., 2015
					Eye diseases	Twigs	Raw	Aziz et al., 2018
					Tonic	Fruits	Raw	Aziz et al., 2018
					Nerve tonic	Leaves	Raw	Khattak et al., 2015
Opuntia littoralis (Engelm.) Cockerell	1	2	2.00	Cactaceae	Inflammation	Whole plant	Powder	Aziz et al., 2018
					Skin problems	Whole plant	Powder	Aziz et al., 2018

Origanum vulgare L.	1	2	2.00	Lamiaceae	Galactagogue	Leaves	Powder	Yousfzai et al., 2010
Oryza sativa L.	9	16	1.78	Poaceae	Respiratory infections	Seeds	Decoction	Abbasi et al., 2013
					Weakness	Seeds	Decoction	Abbasi et al., 2013
					Galactagogue	Seeds	Decoction	Aziz et al., 2018
					Retention of placenta	Seeds	Raw	Dilshad et al., 2008
					Mastitis	Seeds	Decoction	Dilshad et al., 2010
					Diarrhea	Seeds	Decoction	Khan et al., 2015a
					Diarrhea	Seeds	Decoction	Mirani et al., 2014
					Dysentery	Seeds	Decoction	Mirani et al., 2014
					Diarrhea	Seeds	Decoction	Mirani et al., 2016
					Dysentery	Seeds	Decoction	Mirani et al., 2016
					Easy delivery	Seeds	Powder	Shah et al., 2012
					Prolapse	Seeds	Powder	Shah et al., 2012
					Placenta expulsion	Seeds	Powder	Shah et al., 2012
					Bone fracture	Seeds		Ullah et al., 2017
					Neck sore	Seeds		Ullah et al., 2017
					Skin diseases	Seeds		Ullah et al., 2017
Paeonia emodi Royle	6	9	1.50	Paeoniaceae	Cough	Rhizomes, Fruits	Powder	Aziz et al., 2018
					Tonic	Rhizomes, Fruits	Powder	Aziz et al., 2018
					Tonic	Tubers	Powder	Shah et al., 2012
					Lactation	Rhizome	Powder	Sher et al., 2004
					Analgesic	Roots	Decoction	Sindhu et al., 2010
					Internal injuries	Roots	Paste	Ul Hassan et al., 2014
					Analgesic	Rhizomes	Powder	Yousfzai et al., 2010

					Galactagogue	Rhizomes	Powder	Yousfzai et al., 2010
					Tonic	Rhizomes	Powder	Yousfzai et al., 2010
Panicum antidotale Retz.	1	1	1.00	Poaceae	Disinfectant	Whole plant		Harun et al., 2017
Papaver hybridum L.	1	1	1.00	Papaveraceae	Anestrus	Petals	Powder	Dilshad et al., 2008
Papaver somniferum L.	4	5	1.25	Papaveraceae	Analgesic	Latex		Aziz et al., 2018
					Wound	Latex		Aziz et al., 2018
					Worm infestation	Seeds		Badar et al., 2017
					Vaginal prolapse	Fruits	Powder	Khan et al., 2015a
					Prolapse	Fruits		Ullah et al., 2017
Pedalium murex L.	1	1	1.00	Pedaliaceae	Diuretic	Fruits		Khan, 2009
Peganum harmala L.	9	19	2.11	Nitrariaceae	Gastric ailments	Leaves	Raw	Abbasi et al., 2013
					Mastitis	Leaves	Smoke	Abbasi et al., 2013
					Tick infestation	Seeds	Smoke	Abbasi et al., 2013
					Dermatological problems	Leaves	Infusion, Decoction	Ahmad et al., 2015
					Ectoparasites	Leaves	Infusion, Decoction	Ahmad et al., 2015
					Mange	Leaves	Infusion, Decoction	Ahmad et al., 2015
					Multisystem	Leaves	Infusion, Decoction	Ahmad et al., 2015
					Refrigerant	Leaves	Infusion, Decoction	Ahmad et al., 2015
					Antiparasitic	Seeds	Infusion	Aziz et al., 2018
					Gastrointestinal problems	Whole plant	Raw	Aziz et al., 2018

					Skin diseases	Whole plant	Decoction	Aziz et al., 2018
					Mastitis	Fruits, Stems	Smoke	Dilshad et al., 2010
					Analgesic	Seeds, Whole plant	Smoke	Khattak et al., 2015
					Lice infestation	Seeds, Whole plant	Smoke	Khattak et al., 2015
					Wound	Seeds, Whole plant	Smoke	Khattak et al., 2015
					Mastitis	Whole plant	Smoke	Mussarat et al., 2014
					Lice infestation	Whole plant	Extract	Shah et al., 2012
					Mange	Seeds	Extract	Sindhu et al., 2012
					Fever	Seeds		Ullah et al., 2017
Pennisetum glaucum (L.) R.Br.	3	5	1.67	Poaceae	Placenta expulsion	Stems	Raw	Ali et al., 2017
					Genital prolapse	Seeds	Powder	Dilshad et al., 2008
					Foot Diseases	Seeds	Powder	Mirani et al., 2016
					Galactagogue	Seeds	Powder	Mirani et al., 2016
					Mouth diseases	Seeds	Powder	Mirani et al., 2016
Pennisetum orientale Rich.	1	1	1.00	Poaceae	Mouth diseases	Whole plant		Harun et al., 2017
Periploca aphylla Decne.	2	2	1.00	Apocynaceae	Skin infection	Latex		Abbasi et al., 2013
					Cough	Stems	Decoction	Khattak et al., 2015
Perovskia abrotanoides Kar.	1	1	1.00	Lamiaceae	Intestinal worm	Leaves	Raw	Ali et al., 2017
Persicaria amplexicaulis (D.Don) Ronse Decr.	3	5	1.66	Polygonaceae	Dehydration	Roots	Decoction	Abbasi et al., 2013
					Fever	Roots	Decoction	Abbasi et al., 2013

					Indigestion	Leaves	Raw	Abbasi et al., 2013
					Paralysis	Roots	Powder	Sher et al., 2004
					Paralysis	Rhizomes		Yousfzai et al., 2010
Persicaria bistorta (L.) Samp.	1	1	1.00	Polygonaceae	Mastitis	Barks	Decoction	Dilshad et al., 2010
Phalaris minor Retz.	1	1	1.00	Poaceae	Cough	Aerial parts		Harun et al., 2017
Phlomis bracteosa Royle ex Benth.	1	3	3.00	Lamiaceae	Cough	Leaves, Roots, Flowers		Khan et al., 2015b
					Fever	Leaves, Roots, Flowers		Khan et al., 2015b
					Skin diseases	Leaves, Roots, Flowers		Khan et al., 2015b
Phoenix dactylifera L.	4	5	1.25	Arecaceae	Delayed puberty	Fruits	Powder	Dilshad et al., 2008
					Silent estrous	Fruits	Powder	Dilshad et al., 2008
					Wound	Leaves	Ash	Khan and Hanif, 2006
					Infertility	Fruits	Raw	Mirani et al., 2014
					Infertility	Fruits	Raw	Mirani et al., 2016
Phragmites australis (Cav.) Trin. ex Steud.	1	1	1.00	Poaceae	Digestive disorders	Leaves		Harun et al., 2017
Phyllanthus acidus (L.) Skeels	1	1	1.00	Phyllanthaceae	Antidote	Leaves, Roots	Powder	Khan and Hanif, 2006
Phyllanthus niruri L.	2	6	3.00	Phyllanthaceae	Sores	Whole plant	Juice	Khan and Hanif, 2006
					Swollen palts	Leaves, Roots	Powder	Khan and Hanif, 2006
					Ulcers	Leaves,	Powder	Khan and Hanif,

						Roots		2006
					Indigestion	Roots	Powder	Khan, 2009
					Inflammation	Leaves	Juice	Khan, 2009
					Wound	Leaves	Juice	Khan, 2009
Phyllodium pulchellum (L.) Desv.	1	2	2.00	Fabaceae	Fever	Roots	Decoction	Abbasi et al., 2013
					Weakness	Roots	Decoction	Abbasi et al., 2013
Phytolacca latbenia (Moq.) H. Walter	1	1	1.00	Phytolaccaceae	Fever	Rhizome	Powder	Sher et al., 2004
Picrorhiza kurroa Royle ex Benth.	1	3	3.00	Plantaginaceae	Halitosis	Fruits		Muhammad et al., 2005
					Indigestion	Fruits		Muhammad et al., 2005
					Systemic disorders	Fruits		Muhammad et al., 2005
Pinus gerardiana Wall. ex D.Don	3	23	7.67	Pinaceae	Abdominal pain	Barks	Decoction	Ahmad et al., 2015
					Abdominal pain	Wood	Oil	Ahmad et al., 2015
					Bone fracture	Barks	Raw	Ahmad et al., 2015
					Contagious pleuropneumonia	Wood	Oil	Ahmad et al., 2015
					Cough	Wood	Oil	Ahmad et al., 2015
					Dermatological problems	Wood	Oil	Ahmad et al., 2015
					Digestion	Wood	Oil	Ahmad et al., 2015
					Foot diseases	Wood	Oil	Ahmad et al., 2015
					Gastric ailments	Wood	Oil	Ahmad et al., 2015
					Mange	Wood	Oil	Ahmad et al., 2015
					Medicinal food	Wood	Oil	Ahmad et al., 2015
					Mouth diseases	Wood	Oil	Ahmad et al., 2015
					Multisystem	Wood	Oil	Ahmad et al., 2015

					Respiratory	Wood	Oil	Ahmad et al., 2015
					Ritual	Wood	Oil	Ahmad et al., 2015
					Skeletomuscular ailments	Wood	Oil	Ahmad et al., 2015
					Worms in abdomin	Resin	Oil	Ahmad et al., 2015
					Wound	Resin		Ahmad et al., 2015
					Wound	Wood	Oil	Ahmad et al., 2015
					Skin diseases	Gums	Oil	Aziz et al., 2018
					Wound	Gums	Gum	Aziz et al., 2018
					Infection	Wood	Oil	Raziq et al., 2010
Pinus roxburghii Sarg.	4	7	1.75	Pinaceae	Skin problems	Latex	Latex	Aziz et al., 2018
					Mange	Wood	Oil	Farooq et al., 2008
					Abscess	Wood	Resin	Khan and Hanif, 2006
					Broken horns	Wood	Resin	Khan and Hanif, 2006
					Snake bite	Wood	Resin	Khan and Hanif, 2006
					Wound	Wood	Resin	Khan and Hanif, 2006
					Myiasis	Wood	Resin	Sindhu et al., 2010
Pinus wallichiana A.B.Jacks.	1	16	16.00	Pinaceae	Abdominal pain	Wood	Oil	Ahmad et al., 2015
					Contagious pleuropneumonia	Wood	Oil	Ahmad et al., 2015
					Dermatological Problems	Wood	Decoction, Oil, Infusion	Ahmad et al., 2015
					Gastric ailments	Wood	Oil	Ahmad et al., 2015
					Intestinal ailments	Wood	Decoction	Ahmad et al., 2015

					Mange	Wood	Oil	Ahmad et al., 2015
					Medicinal food	Wood	Oil	Ahmad et al., 2015
					Multisystem	Stems, Barks	Decoction	Ahmad et al., 2015
					Respiratory	Wood	Oil	Ahmad et al., 2015
					Ritual	Stems, Barks	Decoction	Ahmad et al., 2015
					Skeletomuscular ailments	Stems, Barks	Decoction	Ahmad et al., 2015
					Skeletomuscular ailments	Stems, Barks	Decoction, Oil, Infusion	Ahmad et al., 2015
					Tonic	Wood	Oil	Ahmad et al., 2015
					Worms in abdomin	Stems Barks	Decoction	Ahmad et al., 2015
					Wound	Wood	Oil	Ahmad et al., 2015
					Wound	Resin	Resin	Ahmad et al., 2015
Piper betle L.	1	1	1.00	Piperaceae	Cough	Leaves	Decoction	Khan et al., 2015a
Piper nigrum L.	9	17	1.89	Piperaceae	Skin infections	Fruits	Raw	Aziz et al., 2018
					Allergy	Fruits	Raw	Badar et al., 2017
					Indigestion	Fruits	Raw	Badar et al., 2017
					Anorexia	Fruits	Powder	Deeba et al., 2009
					Endoparasites	Fruits	Powder	Deeba et al., 2009
					Mastitis	Fruits	Powder	Deeba et al., 2009
					Genital prolapse	Fruits	Raw	Dilshad et al., 2008
					Cough	Fruits	Powder	Khan et al., 2015a
					Flatulance	Fruits	Powder	Khan et al., 2015a
					Mastitis	Fruits	Powder	Khan et al., 2015a
					Halitosis	Fruits		Muhammad et al., 2005
					Indigestion	Fruits		Muhammad et al., 2005

					Systemic disorders	Fruits		Muhammad et al., 2005
					Mastitis	Fruits	Raw	Mussarat et al., 2014
					Mange	Fruits	Powder	Sindhu et al., 2012
					Cough	Fruits		Ullah et al., 2017
					Fever	Fruits		Ullah et al., 2017
Plantago depressa Willd.	1	1	1.00	Plantaginaceae	Laxative	Seeds		Khattak et al., 2015
Plantago lanceolata L.	2	5	2.50	Plantaginaceae	Neck rashes	Leaves	Powder	Abbasi et al., 2013
					Diarrhea	Husk		Mirani et al., 2014
					Dysentery	Husk		Mirani et al., 2014
					Diarrhea	Husk		Mirani et al., 2016
					Dysentery	Husk		Mirani et al., 2016
Plantago major L.	2	4	2.00	Plantaginaceae	Infected hooves	Whole plant	Powder	Abbasi et al., 2013
					Foot diseases	Whole plant	Powder	Shah et al., 2012
					Infected hooves	Whole plant	Powder	Shah et al., 2012
					Mouth diseases	Whole plant	Powder	Shah et al., 2012
Plantago ovata Forssk.	1	1	1.00	Plantaginaceae	Mastitis	Seeds		Khan et al., 2015a
Platanus orientalis L.	2	2	1.00	Platanaceae	Digestive disorders	Barks	Powder	Aziz et al., 2018
					Cough	Barks	Decoction	Khan et al., 2015a
Pleurospermum candollei Benth. ex C.B.Clarke	2	2	1.00	Apiaceae	Diarrhea	Leaves, Roots		Khan et al., 2015a
Poa annua L.	1	1	1.00	Poaceae	Wound	Whole plant		Harun et al., 2017
Polygonatum multiflorum (L.) All.	1	1	1.00		Removal of placenta	Roots	Powder	Sher et al., 2004
Polygonatum verticillatum (L.)	2	2	1.00	Asparagaceae	Galactagogue	Rhizome	Powder	Shah et al., 2012

All.						s		
					Removal of placenta	Roots	Powder	Sher et al., 2004
Polygonum plebeium R.Br.	1	1	1.00	Polygonaceae	Scorpion bite	Whole plant	Raw	Abbasi et al., 2013
Populus caspica Bornm.	1	1	1.00	Salicaeae	Haemomhoids	Shoots	Powder	Khan and Hanif, 2006
Portulaca oleracea L.	1	1	1.00	Portulacaceae	Mastitis	Whole plant	Raw	Mussarat et al., 2014
Primula denticulata Sm.	3	5	1.66	Primulaceae	Fever	Whole plant	Decoction	Ahmed and Murtaza, 2015
					Dysuria	Whole plant	Decoction	Ahmed and Murtaza, 2015
					Hemoglobinuria	Whole plant	Decoction	Ahmed and Murtaza, 2015
					Eye diseases	Stems	Decoction	Aziz et al., 2018
					Eye diseases	Leaves	Extract	Yousfzai et al., 2010
Primula rosea Royle	1	1	1.00	Primulaceae	Ophthalmic diseases	Whole plant		Khan et al., 2015b
Prosopis cineraria (L.) Druce	4	7	1.75	Fabaceae	Rheumatism	Flowers	Poultice	Khan, 2009
					Diarrhea	Leaves	Decoction	Mirani et al., 2014
					Dysentery	Leaves	Decoction	Mirani et al., 2014
					Diarrhea	Leaves	Decoction	Mirani et al., 2016
					Dysentery	Leaves	Decoction	Mirani et al., 2016
					Myiasis	Barks	Powder	Mirani et al., 2016
					Rheumatism	Flowers		Ullah et al., 2017
Prosopis glandulosa Torr.	1	2	2.00	Fabaceae	Analgesic	Leaves	Poultice	Khan, 2009
					Bone fracture	Leaves	Poultice	Khan, 2009
Prunus armeniaca L.	2	7	3.50	Rosaceae	Constipation	Seeds	Oil	Ali et al., 2017
					Diarrhea	Seeds	Oil	Ali et al., 2017
					Ectoparasites	Seeds	Oil	Ali et al., 2017
					Foot, mouth	Seeds	Oil	Ali et al., 2017

					diseases			
					Mange	Seeds	Oil	Ali et al., 2017
					Abdominal pain	Gum	Gum	Aziz et al., 2018
					Appetizer	Gum	Gum	Aziz et al., 2018
Prunus dulcis (Mill.) D.A.Webb	1	1	1.00	Rosaceae	Bloat	Seeds	Oil	Ali et al., 2017
Prunus persica (L.) Batsch	8	11	1.38	Rosaceae	Dysentery	Leaves	Decoction	Abbasi et al., 2013
					Worms killer	Leaves	Juice	Abbasi et al., 2013
					Placenta expulsion	Fruits	Juice	Ali et al., 2017
					Wound	Fruits	Juice	Ali et al., 2017
					Helminthes	Leaves	Powder	Hussain et al., 2008
					Worms	Leaves	Powder	Khan and Hanif, 2006
					Wound	Leaves	Powder	Khan and Hanif, 2006
					Worm infestation	Leaves	Powder	Khan et al., 2015a
					Myiasis	Leaves	Raw	Sindhu et al., 2010
					Helminthiasis	Fruits	Powder	Sindhu et al., 2012
					Worm infestation	Leaves		Ullah et al., 2017
Psidium guajava L.	1	1	1.00	Myrtaceae	Worm infestation	Seeds	Powder	Badar et al., 2017
Punica granatum L.	10	22	2.20	Lythraceae	Dysentery	Fruit rinds	Powder	Abbasi et al., 2013
					Abdominal pain	Fruits	Decoction	Ahmad et al., 2015
					Cough	Fruits	Decoction	Ahmad et al., 2015
					Diarrhea	Fruits	Decoction	Ahmad et al., 2015
					Gastric ailments	Fruits	Decoction	Ahmad et al., 2015
					Intestinal worm	Fruits	Decoction	Ahmad et al., 2015
					Jaundice	Fruits	Decoction	Ahmad et al., 2015
					Medicinal food	Fruits	Decoction	Ahmad et al., 2015

					Respiratory	Fruits	Decoction	Ahmad et al., 2015
					Tonic	Fruits	Decoction	Ahmad et al., 2015
					Abdominal Pain	Exocarpe of Fruits	Powder	Aziz et al., 2018
					Diarrhea	Exocarpe of Fruits	Powder	Aziz et al., 2018
					Dysentery	Exocarpe of Fruits	Powder	Aziz et al., 2018
					Anorexia	Fruits peel	Decoction	Badar et al., 2017
					Worm infestation	Fruits peel	Decoction	Badar et al., 2017
					Diarrhea	Fruits	Powder	Deeba et al., 2009
					Intestinal worm	Periarp of Fruits		Islam et al., 2012
					Vermicides	Periarp of Fruits		Islam et al., 2012
					Diarrhea	Barks	Powder	Khan et al., 2015a
					Digestive disorders	Seeds		Muhammad et al., 2005
					Diarrhea	Fruit rinds	Powder	Mussarat et al., 2014
					Anthelmintic	Fruits, Leaves	Decoction	Tariq et al., 2014
Punica protopunica Balf. f.	1	1	1.00	Lythraceae	Worms	Periarp	Powder	Yousfzai et al., 2010
Putranjiva roxburghii Wall.	1	1	1.00	Putranjivaceae	Ritual	Fruits	Raw	Khan and Hanif, 2006
Pyrus pashia Buch.-Ham. ex D.Don	2	3	1.50	Rosaceae	Diarrhea	Fruits	Powder	Abbasi et al., 2013
					Dysentery	Fruits	Powder	Abbasi et al., 2013
					Inflammation	Fruits		Yousfzai et al., 2010
Quercus baloot Griff.	1	1	1.00	Fagaceae	Urinary problems	Fruits	Raw	Ul Hassan et al., 2014

Quercus floribunda Lindl. ex A.Camus	1	1	1.00	Fagaceae	Urinary problems	Fruits	Powder	Yousfzai et al., 2010
Quercus incana Bartram	1	1	1.00	Fagaceae	Galactagogue	Leaves, Seeds	Powder	Shah et al., 2012
Quercus spp	3	7	2.33	Fagaceae	Astringent,	Bark, Leaves	Decoction	Ahmed and Murtaza, 2015
					Antiasthma	Bark, Leaves	Decoction	Ahmed and Murtaza, 2015
					Diuretic	Bark, Leaves	Decoction	Ahmed and Murtaza, 2015
					Anorex	Bark, Leaves	Decoction	Ahmed and Murtaza, 2015
					Diarrhea	Barks	Decoction	Aziz et al., 2018
					Tonic	Fruits	Raw	Aziz et al., 2018
					Urinary tract diseases.	Fruits	Powder	Sher et al., 2004
Ranunculus muricatus L.	3	6	2.00	Ranunculaceae	Cardiac problems	Aerial parts	Decoctionn	Khuroo et al., 2007
					Fever	Aerial parts	Decoction	Khuroo et al., 2007
					Increase body temperature	Aerial parts	Decoction	Khuroo et al., 2007
					Nausea	Aerial parts	Decoction	Khuroo et al., 2007
					Purgative	Whole plant	Decoction	Shah et al., 2012
					Purgative	Whole plant	Decoction	Yousfzai et al., 2010
Raphanus raphanistrum subsp. *sa tivus* (L.) Domin	4	5	1.25	Brassicaceae	Foot, mouth diseases	Leaves	Raw	Ali et al., 2017
					Placenta expulsion	Leaves	Raw	Ali et al., 2017
					Flatulance	Seeds	Raw	Aziz et al., 2018

					Prolapse	Roots	Raw	Badar et al., 2017
					Liver Problems	Roots	Raw	Khan et al., 2015a
Rhamnus purpurea Edgew.	1	1	1.00	Rhamnaceae	Abdominal worms	Leaves, Fruits	Raw	Khan et al., 2012
Rhazya stricta Decne.	3	6	2.00	Apocynaceae	Blood purification	Leaves	Infusion	Abbasi et al., 2013
					Pain in abdominal	Leaves	Decoction	Abbasi et al., 2013
					Skin infection	Leaves	Infusion	Abbasi et al., 2013
					Allergy	Stems	Smoke	Khattak et al., 2015
					Astringent	Stems	Smoke	Khattak et al., 2015
					Wound	Roots	Ash	Raziq et al., 2010
Rheum australe D. Don	1	2	2.00	Polygonaceae	Constipation	Rhizomes	Powder	Shah et al., 2012
					Wound	Rhizomes	Powder	Shah et al., 2012
Rheum emodi Wall.	1	1	1.00	Polygonaceae	Tonic	Whole plant	Decoction	Ul Hassan et al., 2014
Rheum spiciforme Royle	2	5	2.50	Polygonaceae	Blood diseases	Leaves	Raw	Ali et al., 2017
					Hemorrhagic	Leaves	Raw	Ali et al., 2017
					Blood purification	Roots		Khan et al., 2015b
					Digestive disorders	Roots		Khan et al., 2015b
					Tonic	Roots		Khan et al., 2015b
Rheum webbianum Royle	1	3	3.00	Polygonaceae	Diarrhea	Roots, Stems, Flowers		Khan et al., 2015b
					Digestive disorders	Roots, Stems, Flowers		Khan et al., 2015b
					Laxative	Roots,		Khan et al., 2015b

						Stems, Flowers		
Ribes alpestre Wall. ex Decne.	1	2	2.00	Grossulariaceae	Jaundice	Fruits	Raw	Ahmad et al., 2015
					Skeletomuscular ailments	Fruits	Raw	Ahmad et al., 2015
Ricinus communis L.	15	28	1.87	Euphorbiaceae	Constipation	Seeds	Oil	Abbasi et al., 2013
					Constipation	Oil	Oil	Aziz et al., 2018
					Diarrhea	Oil	Oil	Aziz et al., 2018
					Uterine prolapse	Fruits	Oil	Deeba et al., 2009
					Delayed puberty	Seeds		Dilshad et al., 2008
					Silent estrous	Seeds		Dilshad et al., 2008
					Helminthes	Seeds	Powder	Hussain et al., 2008
					Promote sterility	Seeds	Oil	Islam et al., 2012
					Laxative	Seeds	Oil	Islam et al., 2012
					Appetizer	Seeds	Oil	Khan and Hanif, 2006
					Constipation	Seeds	Oil	Khan and Hanif, 2006
					Easy delivery	Leaves	Oil	Khan and Hanif, 2006
					Genital Prolapse	Leaves	Decoction	Khan and Hanif, 2006
					Infection in uterus	Leaves	Decoction	Khan and Hanif, 2006
					Mange	Leaves	Decoction	Khan and Hanif, 2006
					Prolapse of uterus	Seeds	Oil	Khan et al., 2012
					Constipation	Seeds	Oil	Khan, 2009
					Placenta expulsion	Leaves	Extract	Khan, 2009
					Purgative	Seeds	Oil	Khan, 2009

					Sterility	Seeds	Powder	Khattak et al., 2015
					Uterine prolapse	Leaves	Decoction	Mirania et al., 2016
					Gastrointestinal helminthiasis	Seeds	Powder	Sindhu et al., 2010
					Gastrointestinal helminthiasis	Seeds	Powder	Sindhu et al., 2010
					Myiasis	Seeds	Extract	Sindhu et al., 2010
					Common cold	Leaves	Powder	Tariq et al., 2014
					Gastric ailments	Seeds, Leaves	Extract	Tariq et al., 2016
					Constipation	Seeds	Oil	Ullah et al., 2017
					Retention of placenta	Seeds	Oil	Ullah et al., 2017
Rosa × damascena Herrm.	4	4	1.00	Rosaceae	Anorexia	Flowers	Decoction	Deeba et al., 2009
					Vaginal prolapse	Flowers	Raw	Khan et al., 2015a
					Placenta expulsion	Flowers	Raw	Mirani et al., 2014
					Prolapse of vagina	Flowers		Ullah et al., 2017
Rosa cymosa Tratt.	1	1	1.00	Rosaceae	Uterine prolapse	Flowers	Infusion	Abbasi et al., 2013
Rosa indica L.	3	5	1.67	Rosaceae	Anestrus	Flowers	Decoction	Dilshad et al., 2008
					Mastitis	Flowers	Decoction	Dilshad et al., 2010
					Abdominal pain	Leaves	Extract	Mussarat et al., 2014
					Constipation	Leaves	Extract	Mussarat et al., 2014
					Mastitis	Flowers	Decoction	Mussarat et al., 2014
Rosa moschata Herrm.	1	2	2.00	Rosaceae	Anti-congestion effects	Flowers	Raw	Aziz et al., 2018
					Inflammation	Flowers	Raw	Aziz et al., 2018
Rubus fruticosus L.	2	4	2.00	Rosaceae	Anemia	Stems, Leaves, Fruits		Islam et al., 2012

					Tonic	Stems, Leaves, Fruits		Islam et al., 2012
					Carminative	Leaves, Fruits		Yousfzai et al., 2010
					Diuretic	Leaves, Fruits		Yousfzai et al., 2010
Rumex dentatus L.	4	8	2.00	Polygonaceae	Foot diseases	Roots	Decoction	Abbasi et al., 2013
					Mouth diseases	Roots	Decoction	Abbasi et al., 2013
					Gastric ailments	Roots	Raw	Ahmad et al., 2015
					Intestinal worm	Roots	Raw	Ahmad et al., 2015
					Skeletomuscular ailments	Roots	Raw	Ahmad et al., 2015
					Skeletomuscular ailments	Roots	Raw	Ahmad et al., 2015
					Gastric problems	Leaves	Fodder	Sher et al., 2004
					Constipation	Leaves		Yousfzai et al., 2010
Rumex hastatus D. Don	7	15	2.14	Polygonaceae	Cough	Roots	Decoction	Abbasi et al., 2013
					Fever	Roots	Decoction	Abbasi et al., 2013
					Scabies	Aerial parts	Raw	Abbasi et al., 2013
					Weakness	Roots	Decoction	Abbasi et al., 2013
					Gastric ailments	Whole plant	Decoction	Aziz et al., 2018
					Urinary problems	Whole plant	Decoction	Aziz et al., 2018
					Wounds	Whole plant	Decoction	Aziz et al., 2018
					Wounds	Whole plant		Islam et al., 2012
					Ectoparasitess	Whole	Powder	Shah et al., 2012

						plant		
					Internal injuries	Whole plant	Powder	Shah et al., 2012
					Myiasis	Roots	Raw	Sindhu et al., 2010
					Wound	Roots	Extract	Sindhu et al., 2010
					Wound	Roots, Leaves	Powder	Tariq et al., 2014
					Appetizer	Whole plant	Powder	Ul Hassan et al., 2014
Rumex nepalensis Spreng.	4	14	3.5	Polygonaceae	Antiseptic	Leaves	Extract	Abbasi et al., 2013
					Diarrhea	Roots	Powder	Abbasi et al., 2013
					Dysentery	Roots	Powder	Abbasi et al., 2013
					Hemostatic	Leaves	Powder	Abbasi et al., 2013
					Inflammation	Leaves	Extract	Abbasi et al., 2013
					Intestinal worm	Roots	Powder	Abbasi et al., 2013
					Hepatitis,	Whole plant	Decoction	Ahmed and Murtaza, 2015
					Malaria	Whole plant	Decoction	Ahmed and Murtaza, 2015
					Dysuria	Whole plant	Decoction	Ahmed and Murtaza, 2015
					Urinary problems	Whole plant	Decoction	Ahmed and Murtaza, 2015
					Anthelmintic	Roots	Powder	Khuroo et al., 2007
					Digestive disorders	Roots	Powder	Khuroo et al., 2007
					Infections	Roots Barks	Decoction	Khuroo et al., 2007
					Tonic	Roots	Powder	Shah et al., 2012
Ruta graveolens L.	2	2	1.00	Rutaceae	Retention of placenta	Seeds	Decoction	Dilshad et al., 2008

					Helminthiasis	Bulbs	Powder	Sindhu et al., 2012
Rydingia limbata (Benth.) Scheen & V.A.Albert	1	1	1.00	Lamiaceae	Wound	Leaves	Powder	Shah et al., 2012
Saccharum bengalense Retz.	1	2	2.00	Poaceae	Appetizer	Roots	Decoction	Abbasi et al., 2013
					Worms from intestine	Roots	Decoction	Abbasi et al., 2013
Saccharum officinarum L.	5	4	0.80	Poaceae	Mastitis	Stems	Extract	Dilshad et al., 2010
					Babesioses	Stems	Extract	Khan et al., 2015a
					Hepatitis	Stems	Extract	Khan et al., 2015a
					Mastitis	Stems	Extract	Mussarat et al., 2014
Saccharum spontaneum L.	2	3	1.50	Poaceae	Inflammation	Leaves		Harun et al., 2017
					Urinary problems	Leaves		Harun et al., 2017
					Retention of placenta	Rhizome s	Powder	Khan et al., 2015a
Salix babylonica L.	1	1	1.00	Salicaceae	Skin infections	Leaves	Decoction	Aziz et al., 2018
Salix tetrasperma Roxb.	2	7	3.50	Salicaceae	Contagious pleuropneumoni a	Leaves	Decoction	Ahmad et al., 2015
					Foot diseases	Leaves	Decoction	Ahmad et al., 2015
					Mouth diseases	Leaves	Decoction	Ahmad et al., 2015
					Multisystem	Leaves	Decoction	Ahmad et al., 2015
					Respiratory	Leaves	Decoction	Ahmad et al., 2015
					Cough	Barks	Powder	Aziz et al., 2018
					Infection	Barks	Powder	Aziz et al., 2018
Salix viminalis L.	1	2	2.00	Salicaceae	Febrifuge	Leaves		Islam et al., 2012
					Analgesic	Leaves		Islam et al., 2012
Salsola imbricata Forssk.	1	1	1.00	Amaranthaceae	Helminthiasis	Aerial parts	Infusion	Farooq et al., 2008
Salvadora oleoides Decne.	3	3	1.00	Salvadoraceae	Genital prolapse	Leaves		Dilshad et al., 2008
					Rheumatism	Fruits	Raw	Khan, 2009

					Expulsion of lochia	Fruits	Raw	Khan, 2009
					Placenta expulsion	Roots	Juice	Khattak et al., 2015
Salvadora persica L.	1	2	2.00	Salvadoraceae	Broken horns	Leaves	Ash	Mirani et al., 2016
					Ectoparasites	Roots	Powder	Mirani et al., 2016
Salvia moorcroftiana Wall. ex Benth.	5	7	1.4	Lamiaceae	Gastric ailments	Leaves	Decoction	Aziz et al., 2018
					Fever	Rhizome s	Powder	Shah et al., 2012
					Galactagogue	Rhizome s	Powder	Shah et al., 2012
					Fever	Leaves	Fodder	Sher et al., 2004
					Removal of placenta	Leaves	Fodder	Sher et al., 2004
					Internal injuries	Roots	Decoction	Ul Hassan et al., 2014
					Placenta expulsion	Leaves		Yousfzai et al., 2010
Saussurea costus (Falc.) Lipsch.	3	3	1.00	Asteraceae	Gastrointestinal helminthiasis	Roots	Powder	Sindhu et al., 2010
					Tonic	Rhizome s	Powder	Shah et al., 2012
					Mange	Rhizome s	Powder	Sindhu et al., 2012
Sedum roseum (L.) Scop.	1	2	2.00	Crassulaceae	Analgesic	Stems	Powder	Khuroo et al., 2007
					Wound	Stems	Powder	Khuroo et al., 2007
Semecarpus anacardium L.f.	1	2	2.00	Anacardiaceae	Delayed puberty	Fruits		Dilshad et al., 2008
					Silent estrous	Fruits		Dilshad et al., 2008
Senecio laetus Edgew.	1	2	2.00	Astaraceae	Arthritis	Roots	Decoction	Abbasi et al., 2013
					Sore joints	Whole plant	Raw	Abbasi et al., 2013

Senecio chrysanthemoides DC.	1	3	3.00	Asteraceae	Anthelmintic,	Aerial parts	Decoction	Ahmed and Murtaza, 2015
					Antiscorbutic	Aerial parts	Decoction	Ahmed and Murtaza, 2015
					Diaphoretic	Aerial parts	Decoction	Ahmed and Murtaza, 2015
Senna alexandrina Mill.	2	6	3.00	Fabaceae	Cathartic	Leaves, Pods	Powder	Khan, 2009
					Intestinal worm	Leaves, Pods	Powder	Khan, 2009
					Rheumatism	Leaves, Pods	Powder	Khan, 2009
					Skin diseases	Leaves, Pods	Powder	Khan, 2009
					Stomach problems	Whole plant, Seeds	Decoction	Khattak et al., 2015
					Vermicides	Whole plant, Seeds	Decoction	Khattak et al., 2015
Seriphidium brevifolium (Wall. ex DC.) Ling , Y.R.Ling	4	8	2.00	Asteraceae	Bloat	Leaves	Raw	Ali et al., 2017
					Ectoparasites	Leaves	Raw	Ali et al., 2017
					Intestinal worm	Leaves	Raw	Ali et al., 2017
					Wound	Leaves	Raw	Ali et al., 2017
					Digestion	Leaves	Powder	Sher et al., 2004
					Retention of placenta	Leaves	Decoction	Tariq et al., 2014
					Digestion	Leaves, Flowers		Yousfzai et al., 2010
					Vermicides	Leaves, Flowers		Yousfzai et al., 2010
Sesamum indicum L.	4	7	1.75	Pedaliaceae	Infection of	Seeds	Oil	Aziz et al., 2018

					Vagina			
					Delayed puberty	Seeds	Decoction	Dilshad et al., 2008
					Dystokia	Seeds	Oil	Dilshad et al., 2008
					Genital prolapse	Seeds	Decoction	Dilshad et al., 2008
					Silent estrous	Seeds	Decoction	Dilshad et al., 2008
					Mastitis	Seeds	Oil	Dilshad et al., 2010
					Mastitis	Seeds	Oil	Mussarat et al., 2014
Setaria pumila (Poir.) Roem. & Schult.	1	1	1.00	Poaceae	Infections	Aerial parts		Harun et al., 2017
Setaria verticillata (L.) P.Beauv.	1	1	1.00	Poaceae	Flatulence	Leaves		Harun et al., 2017
Setaria viridis (L.) P.Beauv.	1	1	1.00	Poaceae	Diuretic	Whole plant		Harun et al., 2017
Sibbaldianthe bifurca (L.) Kurtto & T.Erikss.	1	8	8.00	Rosaceae	Abdominal pain	Leaves, Flowers	Raw	Ali et al., 2017
					Analgesic	Leaves, Flowers	Raw	Ali et al., 2017
					Bloat	Leaves, Flowers	Raw	Ali et al., 2017
					Colic	Leaves, Flowers	Raw	Ali et al., 2017
					Constipation	Leaves, Flowers	Raw	Ali et al., 2017
					Diarrhea	Leaves, Flowers	Raw	Ali et al., 2017
					Stomach problems	Leaves, Flowers	Raw	Ali et al., 2017
					Tympany	Leaves, Flowers	Raw	Ali et al., 2017
Sideroxylon mascatense (A.DC.) T.D.Penn.	2	2	1.00	Sapotaceae	Tonic	Fruits	Raw	Aziz et al., 2018
					Abnormal taste of milk	Leaves	Raw	Ul Hassan et al., 2014

Silene villosa Forssk.	1	1	1.00	Caryophyllaceae	Galactagogue	Whole plant		Khattak et al., 2015
Silene vulgaris (Moench) Garcke	1	1	1.00	Caryophyllaceae	Galactagogue	Rhizome s	Powder	Khuroo et al., 2007
Silybum marianum (L.) Gaertn.	1	1	1.00	Asteraceae	Galactagogue	Seeds, Leaves, Flowers		Khan et al., 2015b
Sinopodophyllum hexandrum (Ro yle) T.S.Ying	1	1	1.00	Berberidaceae	Myiasis	Aerial parts	Powder	Sindhu et al., 2010
Sisymbrium irio L.	1	2	2.00	Brassicaceae	Hepatitis	Seeds		Khan et al., 2015a
					Weakness	Seeds		Khan et al., 2015a
Skimmia laureola Franch.	2	3	1.5	Rutaceae	Cold	Leaves	Powder, Decoction	Ahmed and Murtaza, 2015
					Gastric problems	Leaves	Powder, Decoction	Ahmed and Murtaza, 2015
					Gastric problems	Leaves	Powder	Sher et al., 2004
Solanum incanum L.	1	1	1.00	Solanaceae	Mastitis	Roots, Leaves	Powder	Tariq et al., 2016
Solanum melongena L.	1	2	2.00	Solanaceae	Anestrus	Fruits	Raw	Badar et al., 2017
					Constipation	Fruits	Raw	Badar et al., 2017
Solanum surattense Burm. f.	7	17	2.43	Solanaceae	Cough	Aerial parts	Decoction	Abbasi et al., 2013
					Fever	Aerial parts	Decoction	Abbasi et al., 2013
					Indigestion	Aerial parts	Decoction	Abbasi et al., 2013
					Tonic	Fruits	Paste	Abbasi et al., 2013
					Wound	Fruits	Paste	Abbasi et al., 2013
					Genital prolapse	Leaves	Powder	Dilshad et al., 2008
					Myiasis	Fruits	Raw	Farooq et al., 2008

					Appetizer	Whole plant		Islam et al., 2012
					Cough	Whole plant		Islam et al., 2012
					Fever	Whole plant		Islam et al., 2012
					Intestinal infection	Whole plant		Islam et al., 2012
					Colic	Fruits	Decoction	Khan et al., 2015a
					Worm infestation	Fruits	Paste	Khan et al., 2015a
					Cough	Whole plant	Powder	Tariq et al., 2014
					Fever	Whole plant	Powder	Tariq et al., 2014
					Intestinal infections	Whole plant	Powder	Tariq et al., 2014
					Fever	Fruits		Ullah et al., 2017
Solanum tuberosum L.	1	1	1.00	Solanaceae	Bloat	Fruits	Extract	Deeba et al., 2009
Solanum virginianum L.	3	4	1.33	Solanaceae	Cough	Roots, Leaves, Fruits	Decoction	Aziz et al., 2018
					Fever	Roots, Leaves, Fruits	Decoction	Aziz et al., 2018
					Helminthes	Fruits	Powder	Hussain et al., 2008
					Pneumonia	Aerial parts	Ash	Sindhu et al., 2010
Sonchus asper (L.) Hill	1	1	1.00	Asteraceae	Galactagogue	Whole plant	Decoction	Tariq et al., 2014
Sophora mollis (Royle) Baker	2	2	1.00	Fabaceae	Bleeding	Leaves	Raw	Ali et al., 2017
					Internal body	Leaves	Somke	Aziz et al., 2018

					infection			
Sorbaria sorbifolia (L.) A.Braun	1	1	1.00	Rosaceae	Stimulant	Aerial parts	Fodder	Ahmed and Murtaza, 2015
Sorghum bicolor (L.) Moench	3	5	1.67	Poaceae	Retention of placenta	Seeds		Dilshad et al., 2008
					Anaemia	Aerial parts		Harun et al., 2017
					Constipation	Aerial parts		Harun et al., 2017
					Wound	Aerial parts		Harun et al., 2017
					Helminthiasis	Seeds	Powder	Sindhu et al., 2012
Sorghum halepense (L.) Pers.	7	11	1.57	Poaceae	Indigestion	Roots	Decoction	Abbasi et al., 2013
					Inflammation	Roots	Decoction	Harun et al., 2017
					Analgesic	Stems, Leaves, Seeds		Islam et al., 2012
					Antiseptic	Stems, Leaves, Seeds		Islam et al., 2012
					Antiseptic	Stems, Leaves, Seeds		Islam et al., 2012
					Tonic	Stems, Leaves, Seeds		Islam et al., 2012
					Wound	Stems, Leaves, Seeds		Islam et al., 2012
					Mastitis	Roots	Decoction	Khan and Hanif, 2006
					Mastitis	Roots	Decoction	Khan et al., 2012

					Ectoparasites	Rhizome s	Powder	Khan et al., 2015a
					Mastitis	Roots	Decoction	Khattak et al., 2015
Spirea altaica Pall.	1	4	4.00	Rosaceae	Cough	Stems Barks	Decoction	Ahmad et al., 2015
					Diarrhea	Stems Barks	Decoction	Ahmad et al., 2015
					Gastric ailments	Stems Barks	Decoction	Ahmad et al., 2015
					Respiratory	Stems Barks	Decoction	Ahmad et al., 2015
Stachys tibetica Vatke	1	4	4.00	Lamiaceae	Abdominal pain	Leaves	Raw	Ali et al., 2017
					Colic	Leaves	Raw	Ali et al., 2017
					Ectoparasites	Leaves	Raw	Ali et al., 2017
					Stomach pain	Leaves	Raw	Ali et al., 2017
Stellaria media (L.) Vill.	2	2	1.00	Caryophylaceae	Appetizer	Whole plant	Fodder	Sher et al., 2004
					Appetizer	Whole plant		Yousfzai et al., 2010
Streblus asper Lour.	1	2	2.00	Moraceae	Galactagogue	Fruits	Juice	Aziz et al., 2018
					Refrigerant	Fruits	Juice	Aziz et al., 2018
Swertia paniculata Wall.	1	2	2.00	Gentianaceae	Cold	Whole plant	Decoction Powder	Ahmed and Murtaza, 2015
					Indigestion	Whole plant	Decoction Powder	Ahmed and Murtaza, 2015
Swertia petiolata Royle ex D.Don	1	1	1.00	Gentianaceae	Wound	Leaves	Powder	Khuroo et al., 2007
Syzygium aromaticum (L.) Merr. & L.M.Perry	1	1	1.00	Myrtaceae	Dystokia	Fruits	Powder	Dilshad et al., 2008
Syzygium cumini (L.) Skeels	5	5	1.00	Myrtaceae	Diarrhea	Leaves	Raw	Abbasi et al., 2013
					Diarrhea	Leaves	Raw	Badar et al., 2017
					Diarrhea	Leaves	Powder	Deeba et al., 2009
					Genital prolapse	Fruits	Decoction	Dilshad et al., 2008

					Helminthes	Leaves	Powder	Hussain et al., 2008
Tagetes minuta L.	2	2	1.00	Asteraceae	Earache	Leaves	Infusion	Abbasi et al., 2013
					Skin infections	Leaves	Juice	Tariq et al., 2014
Tamarindus indica L.	1	6	6.00	Fabaceae	Galactagogue	Fruits	Raw	Badar et al., 2017
					Genital prolapse	Fruits	Raw	Badar et al., 2017
					Heat stroke	Fruits	Raw	Badar et al., 2017
					Jaundice	Fruits	Raw	Badar et al., 2017
					Mastitis	Fruits	Raw	Badar et al., 2017
					Panting	Fruits	Raw	Badar et al., 2017
Tamarix aphylla (L.) H.Karst.	8	12	1.50	Tamaricaceae	Worms in Wounds	Leaves	Infusion	Abbasi et al., 2013
					Helminthes	Flowers	Powder	Hussain et al., 2008
					Diarrhea	Leaves, Barks	Powder	Khan et al., 2015a
					Fever	Leaves, Barks	Powder	Khan et al., 2015a
					Analgesic	Leaves	Smoke	Khattak et al., 2015
					Foot Diseases	Barks	Smoke	Mirani et al., 2016
					Mouth diseases	Barks	Smoke	Mirani et al., 2016
					Worms in skin	Leaves	Poultice	Mussarat et al., 2014
					Wounds	Leaves	Poultice	Mussarat et al., 2014
					Kill worm	Leaves	Paste	Tariq et al., 2014
					Diarrhea	Barks		Ullah et al., 2017
					Malaise	Barks		Ullah et al., 2017
Tanacetum falconeri Hook.f.	1	2	2.00	Asteraceae	Constipation	Leaves	Raw	Ali et al., 2017
					Wound	Leaves	Raw	Ali et al., 2017
Tanacetum gracile Hook.f. & Thomson	1	2	2.00	Asteraceae	Indigestion	Leaves	Raw	Ali et al., 2017
					Intestinal worms	Leaves	Raw	Ali et al., 2017
Taraxacum campylodes G.E.Hagl und	4	4	1.00	Asteraceae	Galactagogue	Whole plant	Raw	Aziz et al., 2018
					Galactagogue	Whole plant	Raw	Khan and Hanif, 2006

					Galactagogue	Whole plant	Raw	Khan et al., 2012
					Weakness	Aerial parts	Extract	Khuroo et al., 2007
Tecomella undulata (Sm.) Seem.	1	1	1.00	Bignoniaceae	Urinary problems	Whole plant		Khattak et al., 2015
					Skin problems	Whole plant		Khattak et al., 2015
Thalictrum foetidum L.	1	1	1.00	Ranunculaceae	Abdominal pain	Leaves	Raw	Ali et al., 2017
Thymus linearis Benth.	3	4	1.33	Lamiaceae	Digestive disorders	Leaves		Khan et al., 2015b
					Galactagogue	Whole plant	Powder	Shah et al., 2012
					Colic	Fruits	Raw	Yousfzai et al., 2010
					Flatulence	Fruits	Raw	Yousfzai et al., 2010
Thymus mongolicus (Ronniger) Ronniger	2	4	2.00	Lamiaceae	Common cold	Seeds	Powder	Khuroo et al., 2007
					Fever	Seeds	Powder	Khuroo et al., 2007
					Respiratory	Seeds	Powder	Khuroo et al., 2007
					Mastitis	Leaves	Poultice	Mussarat et al., 2014
Tinospora sinensis (Lour.) Merr.	1	1	1.00	Menispermaceae	Skin infections	Whole plant	Poultice	Tariq et al., 2014
Toona ciliata M.Roem.	3	4	1.33	Meliaceae	Dysentery	Barks	Powder	Khan and Hanif, 2006
					Diarrhea	Barks	Powder	Khan et al., 2012
					Dysentery	Barks	Powder	Khan et al., 2012
					Purgative	Leaves	Powder	Shah et al., 2012
Trachyspermum ammi (L.) Sprague	14	50	3.57	Apiaceae	Appetizer	Seeds	Raw	Abbasi et al., 2013
					Galactagogue	Seeds	Raw	Abbasi et al., 2013
					Appetizer	Seeds	Powder	Aziz et al., 2018
					Galactagogue	Seeds	Powder	Aziz et al., 2018
					Placenta expulsion	Seeds	Powder	Aziz et al., 2018

					Anestrus	Seeds	Powder	Badar et al., 2017
					Colic	Seeds	Powder	Badar et al., 2017
					Constipation	Seeds	Powder	Badar et al., 2017
					Fever	Seeds	Powder	Badar et al., 2017
					Tympany	Seeds	Powder	Badar et al., 2017
					Worm infestation	Seeds	Powder	Badar et al., 2017
					Anorexia	Seeds	Powder	Deeba et al., 2009
					Bloat	Seeds	Powder	Deeba et al., 2009
					Diarrhea	Seeds	Powder	Deeba et al., 2009
					Ectoparasites	Seeds	Oil	Deeba et al., 2009
					Endoparasites	Seeds	Decoction	Deeba et al., 2009
					Fever	Seeds	Powder	Deeba et al., 2009
					Genital prolapse	Seeds	Powder	Dilshad et al., 2008
					Retained foetal membrane	Seeds	Decoction	Dilshad et al., 2008
					Cough	Fruits		Islam et al., 2012
					Colic	Fruits	Powder	Khan et al., 2015a
					Constipation	Fruits	Powder	Khan et al., 2015a
					Fever	Fruits	Powder	Khan et al., 2015a
					Flatulence	Fruits	Powder	Khan et al., 2015a
					Off feeding	Fruits	Powder	Khan et al., 2015a
					Pneumonia	Fruits	Powder	Khan et al., 2015a
					Tympney	Fruits	Powder	Khan et al., 2015a
					Bloat	Seeds	Decoction	Mirani et al., 2014
					Galactagogue	Seeds	Powder	Mirani et al., 2014
					Mastitis	Seeds	Powder	Mirani et al., 2014
					Placenta expulsion	Seeds	Powder	Mirani et al., 2014
					Tympany	Seeds	Powder	Mirani et al., 2014
					Bloat	Seeds	Powder	Mirani et al., 2016

					Broken horns	Seeds	Powder	Mirani et al., 2016
					Galactagogue	Seeds	Powder	Mirani et al., 2016
					Mastitis	Seeds	Powder	Mirani et al., 2016
					Placenta expulsion	Seeds	Powder	Mirani et al., 2016
					Respiratory	Seeds	Powder	Mirani et al., 2016
					Tympany	Seeds	Powder	Mirani et al., 2016
					Halitosis	Seeds		Muhammad et al., 2005
					Indigestion	Seeds		Muhammad et al., 2005
					Systemic disorders	Seeds		Muhammad et al., 2005
					Abdominal pain	Seeds	Powder	Mussarat et al., 2014
					Allergy	Seeds	Powder	Mussarat et al., 2014
					Mastitis	Seeds	Powder	Mussarat et al., 2014
					Stomach problems	Seeds	Powder	Mussarat et al., 2014
					Pneumonia	Seeds	Raw	Sindhu et al., 2010
					Mange	Seeds	Extract	Sindhu et al., 2012
					Mange	Seeds	Powder	Sindhu et al., 2012
					Analgesic	Fruits		Ullah et al., 2017
Trianthema portulacastrum L.	1	1	1.00	Aizoaceae	Helminthes	Whole plant	Powder	Hussain et al., 2008
Tribulus terrestris L.	6	9	1.50	Zygophylaceae	Appetizer	Leaves	Powder	Abbasi et al., 2013
					Gastric ailments	Leaves	Powder	Abbasi et al., 2013
					Pain of joint	Leaves	Powder	Abbasi et al., 2013
					Urinary problems	Whole plant	Raw	Ali et al., 2017
					Diarrhea	Whole plant	Decoction	Khan et al., 2015a
					Colic	Leaves	Juice	Khan, 2009

					Cough	Leaves	Juice	Khan, 2009
					Cough	Whole plant	Powder	Tariq et al., 2014
					Diarrhea	Whole plant		Ullah et al., 2017
Trichodesma indicum (L.) Lehm.	4	10	2.50	Boreginaceae	Intestinal worm	Whole plant	Paste	Abbasi et al., 2013
					Stomach disorders	Whole plant	Paste	Abbasi et al., 2013
					Analgesic	Whole plant		Islam et al., 2012
					Inflammation	Whole plant		Islam et al., 2012
					Inflammation	Leaves	Poultice	Khan and Hanif, 2006
					Mastitis	Leaves	Poultice	Khan and Hanif, 2006
					Snake bite	Roots	Decoction	Khan and Hanif, 2006
					Mastitis	Roots	Decoction	Khan et al., 2012
					Inflammation	Leaves	Paste	Khan et al., 2012
					Snake bite	Roots	Decoction	Khan et al., 2012
					Swelling	Leaves	Paste	Khan et al., 2012
Trifolium repens L.	1	2	2.00	Fabaceae	Laxative	Roots	Powder	Tariq et al., 2014
					Tonic	Roots	Powder	Tariq et al., 2014
Trigonella foenum-graecum L.	10	18	1.80	Fabaceae	Diarrhea	Seeds	Raw	Abbasi et al., 2013
					Prolapse in urethra	Seeds	Powder	Abbasi et al., 2013
					Diarrhea	Leaves	Raw	Ali et al., 2017
					Gastric ailments	Seeds	Powder	Aziz et al., 2018
					Delayed puberty	Seeds	Powder	Dilshad et al., 2008
					Gental prolapse	Seeds	Powder	Dilshad et al., 2008

					Silent estrous	Seeds	Powder	Dilshad et al., 2008
					Mastitis	Seeds	Powder	Dilshad et al., 2010
					Diarrhea	Seeds	Powder	Khan et al., 2015a
					Off feeding	Seeds	Powder	Khan et al., 2015a
					Tympany	Seeds	Powder	Khan et al., 2015a
					Bone fracture	Seeds	Powder	Mirani et al., 2016
					Galactagogue	Seeds	Powder	Mirani et al., 2016
					Halitosis	Fruits		Muhammad et al., 2005
					Indigestion	Fruits		Muhammad et al., 2005
					Tick infestation	Seeds	Powder	Sindhu et al., 2012
					Diarrhea	Leaves		Ullah et al., 2017
					Gastric ailments	Leaves		Ullah et al., 2017
Triticum aegilopoides Forssk	1	2	2.00	Poaceae	Bloat	Aerial parts	Decoction	Raza et al., 2014
					Tympany	Aerial parts	Decoction	Raza et al., 2014
Triticum aestivum L.	9	15	1.67	Poaceae	Dysentery	Seeds	Raw	Abbasi et al., 2013
					Galactagogue	Seeds	Raw	Abbasi et al., 2013
					Mouth diseases	Seeds	Powder	Abbasi et al., 2013
					Sex tonic	Seeds	Decoction	Aziz et al., 2018
					Delayed puberty	Seeds	Raw	Dilshad et al., 2008
					Silent estrous	Seeds	Raw	Dilshad et al., 2008
					Weakness	Seeds	Raw	Khan et al., 2015a
					Foot diseases	Seeds	Powder	Mirani et al., 2014
					Mouth diseases	Seeds	Powder	Mirani et al., 2014
					Foot diseases	Seeds	Powder	Mirani et al., 2016
					Diarrhea	Seeds	Powder	Mussarat et al., 2014
					Common cold	Seeds	Powder	Tariq et al., 2014
					Dysentery	Seeds	Powder	Tariq et al., 2014
					Diarrhea	Whole		Ullah et al., 2017

						plant		
					Skin problems	Whole plant		Ullah et al., 2017
Tulipa clusiana DC.	1	1	1.00	Liliaceae	Galactagogue	Flowers	Raw	Aziz et al., 2018
Urtica dioica L.	1	1	1.00	Urticaceae	Galactagogue	Whole plant	Fodder	Sher et al., 2004
Valeriana jatamansi Jones	2	4	2.00	Caprifoliaceae	Diarrhea	Leaves	Extract	Ahmad et al., 2015
					Analgesic	Rhizome s	Powder	Khuroo et al., 2007
					Eye diseases	Rhizome s	Powder	Khuroo et al., 2007
					Eye diseases	Rhizome s	Powder	Khuroo et al., 2007
Verbascum thapsus L.	5	7	1.40	Scrophulariacea e	Diarrhea	Whole plant	Paste	Abbasi et al., 2013
					Analgesic	Aerial parts		Islam et al., 2012
					Stomachic problems	Aerial parts		Islam et al., 2012
					Flatulence	Aerial parts	Powder	Khuroo et al., 2007
					Diarrhea	Leaves	Powder	Shah et al., 2012
					Diarrhea	Whole plant		Yousfzai et al., 2010
					Dysentery	Whole plant		Yousfzai et al., 2010
Verbena officinalis L.	1	1	1.00	Verbenaceae	Wound	Stems,Le aves	Decoction	Tariq et al., 2014
Viburnum cotinifolium D. Don	1	1	1.00	Caprifoliaceae	Retention of placenta	Fruits	Raw	Shah et al., 2012
Viburnum nervosum D. Don	1	1	1.00	Caprifoliaceae	Removal of placenta.	Leaves	powder	Sher et al., 2004

Vigna mungo (L.) Hepper	1	2	2.00	Fabaceae	Analgesic	Seeds	Decoction	Khuroo et al., 2007
					Easy delivery	Seeds	Decoction	Khuroo et al., 2007
Viola biflora L.	1	1	1.00	Violaceae	Fever	Leaves	Decoction	Khan et al., 2015a
Viola kashmiriana W. Becker	1	7	7.00	Violaceae	Bronchitis	Whole plant	Decoction	Khan and Hanif, 2006
					Foot diseases	Whole plant	Decoction	Khan and Hanif, 2006
					Mouth diseases	Whole plant	Decoction	Khan and Hanif, 2006
					Sores	Whole plant		Khan and Hanif, 2006
					Swollen	Whole plant	Decoction	Khan and Hanif, 2006
					Ulcers	Whole plant		Khan and Hanif, 2006
					Wound	Whole plant	Decoction	Khan and Hanif, 2006
Viola stocksii Boiss.	1	1	1.00	Violaceae	Dysentery	Whole plant		Khattak et al., 2015
Vitex negundo L.	7	16	2.29	Lamiaceae	Worm infestation	Seeds	Powder	Badar et al., 2017
					Stomach problems	Leaves		Islam et al., 2012
					Arthritis	Seeds	Infusion	Khan, 2009
					Rheumatism	Seeds	Infusion	Khan, 2009
					Vermicides	Leaves	Powder	Khan, 2009
					Flatulence	Whole plant	Juice	Khattak et al., 2015
					Fever	Leaves	Powder	Shah et al., 2012
					Foetid discharge	Leaves	Juice	Shah et al., 2012
					Stomach problems	Leaves	Powder	Shah et al., 2012

					Ulcers	Leaves	Juice	Shah et al., 2012
					Worms	Leaves	Juice	Shah et al., 2012
					Fever	Stems	Powder, Decoction	Tariq et al., 2014
					Mange	Stems	Powder, Decoction	Tariq et al., 2014
					Stomach problems	Stems	Powder, Decoction	Tariq et al., 2014
					Rheumatism	Seeds, Leaves	Infusion, Paste	Tariq et al., 2016
					Wound	Seeds, Leaves	Infusion, Paste	Tariq et al., 2016
Vitis vinifera L.	3	3	1.00	Vitaceae	Hemoglobinuria	Wood, Leaves	Ash	Abbasi et al., 2013
					Wound	Fruits	Powder	Khan et al., 2015a
					Helminthiasis	Seeds	Powder	Sindhu et al., 2012
Withania coagulans (Stocks) Dunal	9	23	2.56	Solanaceae	Dermatological problems	Fruits, Leaves	Decoction	Ahmad et al., 2015
					Digestive disorders	Fruits, Leaves	Decoction	Ahmad et al., 2015
					Gastric ailments	Fruits, Leaves	Decoction	Ahmad et al., 2015
					Jaundice	Fruits, Leaves	Decoction	Ahmad et al., 2015
					Multisystem	Fruits, Leaves	Decoction	Ahmad et al., 2015
					Pain in abdominal	Fruits, Leaves	Decoction	Ahmad et al., 2015
					Refrigerant	Fruits, Leaves	Decoction	Ahmad et al., 2015
					Respiratory	Fruits, Leaves	Decoction	Ahmad et al., 2015

					Skeletomuscular ailments	Fruits, Leaves	Decoction	Ahmad et al., 2015
					Skeleto-muscular ailments	Fruits, Leaves	Decoction	Ahmad et al., 2015
					Sun stroke	Fruits, Leaves	Decoction	Ahmad et al., 2015
					Wound	Fruits, Leaves	Decoction	Ahmad et al., 2015
					Diarrhea	Seeds	Powder	Aziz et al., 2018
					Prolapse	Aerial parts	Powder	Badar et al., 2017
					Worm infestation	Aerial parts	Powder	Badar et al., 2017
					Anorexia	Fruits	Powder	Deeba et al., 2009
					Diarrhea	Fruits	Raw	Deeba et al., 2009
					Carminative	Fruits		Khattak et al., 2015
					Gastric ailments	Fruits		Khattak et al., 2015
					Digestive disorders	Leaves		Muhammad et al., 2005
					Diarrhea	Leaves, Fruits	Powder	Mussarat et al., 2014
					Trypanosomiasis	Fruits	Infusion	Raziq et al., 2010
					Mange	Leaves	Powder	Sindhu et al., 2012
Withania somnifera (L.) Dunal	7	8	1.14	Solanaceae	Mastitis	Roots	Paste	Abbasi et al., 2013
					Galactagogue	Leaves	vegetable	Aziz et al., 2018
					Tonic	Leaves	vegetable	Aziz et al., 2018
					Off feeding	Fruits	Powder	Khan et al., 2015a
					Diarrhea	Roots	Powder	Khattak et al., 2015
					Gastric ailments	Whole plant	Decoction	Tariq et al., 2016

					Off feeding	Fruits		Ullah et al., 2017
					Wound	Seeds	Powder	Yousfzai et al., 2010
Xanthium strumarium L.	2	2	1.00	Asteraceae	Maggots from wound	Leaves	Juice	Shah et al., 2012
					Analgesic	Whole plant		Yousfzai et al., 2010
Zanthoxylum armatum DC.	7	16	2.29	Rutaceae	Indigestion	Leaves	Powder	Abbasi et al., 2013
					Vomiting	Leaves	Powder	Abbasi et al., 2013
					Fever	Fruits	Powder	Aziz et al., 2018
				.	Tonic	Fruits	Raw	Aziz et al., 2018
					Febrifuge	Leaves, Fruits		Islam et al., 2012
					Fever	Leaves, Fruits		Islam et al., 2012
					Stomachic problems	Leaves, Fruits		Islam et al., 2012
					Diarrhea	Seeds	Raw	Khan et al., 2012
					Gastric ailments	Fruits	Powder	Khan et al., 2015a
					Fever	Seeds	Powder	Shah et al., 2012
					Foot diseases	Seeds	Paste	Shah et al., 2012
					Mouth diseases	Seeds	Paste	Shah et al., 2012
					Induce early pregnancy	Fruits	Paste	Ul Hassan et al., 2014
					Anthalmentic	Fruits, Seeds, Barks		Yousfzai et al., 2010
					Carminative	Fruits, Seeds, Barks		Yousfzai et al., 2010
					Galactagogue	Fruits, Seeds, Barks		Yousfzai et al., 2010

Zea mays L.	6	11	1.83	Poaceae	Placenta expulsion	Seeds	Powder	Aziz et al., 2018
					Tonic	Seeds	Powder	Aziz et al., 2018
					Genital prolapse	Seeds	Powder	Dilshad et al., 2008
					Skin problems	Leaves		Harun et al., 2017
					Sores	Leaves		Harun et al., 2017
					Diarrhea	Seeds	Powder	Khan et al., 2015a
					Pneumonia	Seeds	Raw	Khan et al., 2015a
					Weakness	Seeds	Raw	Khan et al., 2015a
					Urinary problems	Seeds	Raw	Shah et al., 2012
					Diarrhea	Seeds		Ullah et al., 2017
					Ectoparasites	Seeds		Ullah et al., 2017
Zingiber officinale Roscoe	9	20	2.22	Zingiberaceae	Gastric ailments	Roots	Powder	Aziz et al., 2018
					Anorexia	Roots	Powder	Badar et al., 2017
					Cough	Rhizome s	Powder	Deeba et al., 2009
					Fever	Rhizome s	Powder	Deeba et al., 2009
					Lochial discharge	Rhizome s	Raw	Dilshad et al., 2008
					Mastitis	Rhizome s	Powder	Dilshad et al., 2010
					Babesioses	Rhizome s	Powder	Khan et al., 2015a
					Cough	Rhizome s	Powder	Khan et al., 2015a
					Fever	Rhizome s	Powder	Khan et al., 2015a
					Mastitis	Rhizome s	Powder	Khan et al., 2015a

					Off feeding	Rhizome s	Powder	Khan et al., 2015a
					Tympany	Rhizome s	Powder	Khan et al., 2015a
					Digestive disorders	Bulbs		Muhammad et al., 2005
					Halitosis	Fruits		Muhammad et al., 2005
					Indigestion	Fruits		Muhammad et al., 2005
					Diarrhea	Rhizome s	Powder	Mussarat et al., 2014
					Mastitis	Rhizome s	Powder	Mussarat et al., 2014
					Cough	Rhizome s		Ullah et al., 2017
					Fever	Rhizome s		Ullah et al., 2017
					Weakness	Rhizome s		Ullah et al., 2017
					Helminthiasis	Rhizome s	Raw	Farooq et al., 2008
Ziziphus jujuba Mill.	5	12	2.40	Rhamnaceae	Anthelmintic	Fruits, Leaves	Decoction	Aziz et al., 2018
					Diuretic	Fruits, Leaves	Decoction	Aziz et al., 2018
					Gastric ailments	Fruits, Leaves	Decoction	Aziz et al., 2018
					Prolapse	Leaves	Decoction	Badar et al., 2017
					Wound	Leaves	Decoction	Badar et al., 2017
					Helminthes	Leaves	Powder	Hussain et al., 2008
					Dysentery	Leaves	Decoction	Khattak et al., 2015

					Galactagogue	Leaves	Raw	Khattak et al., 2015
					Laxative	Fruits	Raw	Khattak et al., 2015
					Cough	Fruits, Leaves	Powder	Tariq et al., 2016
					Fever	Fruits, Leaves	Powder	Tariq et al., 2016
					Wound	Fruits, Leaves	Powder	Tariq et al., 2016
Ziziphus nummularia (Burm.f.) Wight & Arn.	7	10	1.43	Rhamnaceae	Placenta expulsion	Leaves	Decoction	Abbasi et al., 2013
					Anestrus	Leaves	Raw	Dilshad et al., 2008
					Itching	Leaves	Paste	Khan, 2009
					Ulcers	Leaves	Raw	Khan, 2009
					Wound	Leaves	Paste	Khan, 2009
					Wound	Leaves	Decoction	Tariq et al., 2014
					Wound	Leaves	Paste	Tariq et al., 2016
					Wound	Leaves		Ullah et al., 2017
					Wound	Leaves		Ullah et al., 2017
					Galactagogue	Leaves		Yousfzai et al., 2010
Ziziphus oxyphylla Edgew.	1	1	1.00	Rhamnaceae	Infection of liver	Roots	Decoction	Aziz et al., 2018
